# Autonomous submersible multiport water sampler

**DOI:** 10.1016/j.ohx.2021.e00197

**Published:** 2021-04-22

**Authors:** David A. Mucciarone, Hans B. DeJong, Robert B. Dunbar, Yui Takeshita, Rebecca Albright, Keaton Mertz

**Affiliations:** a Stanford University, Earth System Science, Stanford, CA 94305, United States; b Monterey Bay Aquarium Research Institute, Moss Landing, CA 95039, United States; c California Academy of Sciences, San Francisco, CA 94118, United States

**Keywords:** Aquaculture, Ecology, Underwater, Autonomous, Pumping, Sampling

## Abstract

Oceanography and limnology projects often require the collection of water samples for chemical analysis. Manual water sample collection is labor-intensive and often difficult, especially in remote locations or during nighttime hours. Here we describe a compact and inexpensive autonomous submersible multiport water sampler (AutoSampler) that is largely fabricated with off-the-shelf parts making it easier to build and maintain. The system can collect up to 12 discrete samples at user controllable times or intervals and is operated using open source Arduino hardware and software that can be user modified to meet deployment requirements. While the underwater pressure housing presented here is custom built from readily available materials, there are many commercially available pressure case options that can be used as a substitute. The electronic mounting plates and battery pack are designed so that they can easily be adapted to fit into other pressure case housings. Samples can be collected into bags or syringes and sample volume is set by adjusting how long the peristaltic pump is actuated. This AutoSampler allows research that would otherwise be too labor-intensive or logistically difficult to conduct, especially in remote locations.

## Graphical abstract

**Figure:**
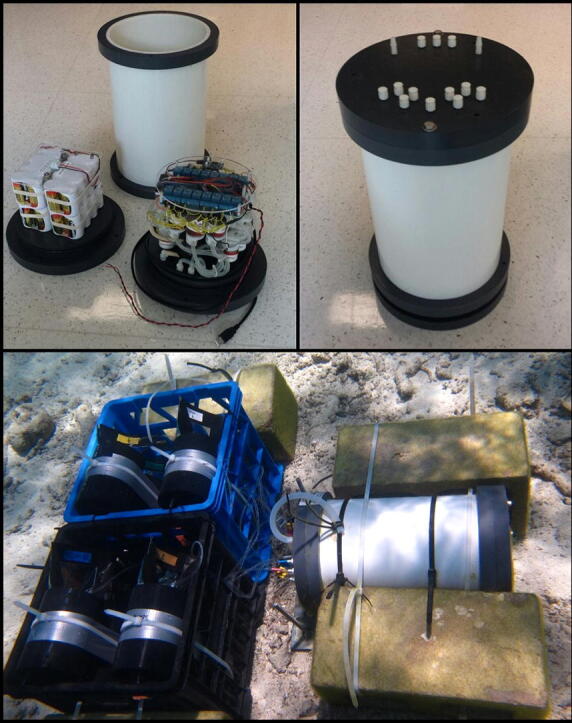

**Specifications table:** 

Hardware name	Autonomous submersible multiport water sampler (AutoSampler)
Subject area	• Oceanography and Limnology • Chemistry and Biochemistry • Biological Sciences and Ecology • Environmental and Aquaculture Sciences
Hardware type	• Measuring water physical and chemical properties • Field measurements and sensors • Autonomous submersible water sampling
Open Source License	GNU General Public License (GPL) 3.0
Cost of Hardware	$2,786
Source File Repository	https://doi.org/10.17605/OSF.IO/E7NJM

## Hardware in context

Oceanography, limnology, and water quality projects often require the collection of water samples for chemical analysis. However, in many cases the collection of water samples is labor-intensive and inconvenient. For example, samples may require collection at regular intervals over a sustained period of time (e.g., daily water samples over weeks) or may need to be collected at higher frequencies throughout a 24-hour period, including nighttime. Water may also need to be collected at different water depths over a period of time. The logistical challenges become especially demanding in remote locations, particularly during the night. Here we present a lightweight, cost-effective AutoSampler that is designed to function in many coastal settings, including coral reefs, seagrass beds, lakes, ponds, and estuaries as well as in aquaculture and aquarium tanks. The AutoSampler is capable of collecting up to 12 samples at preprogrammed intervals or times, and is built using off-the-shelf components except for the pressure case housing and end caps.

Early attempts to develop autonomous submersible water samplers for example [1], [2], [3], [4], [5], [6] were met with technology limitations of the time and were often bulky and heavy limiting their application especially for coastal systems and shallow water deployments. Since then, several commercially available water samplers have been designed for use in deep and shallow environments. McLane Research Laboratories [7] offer a Remote Access Sampler (RAS) that is a multiport water sampling system that can take up to 48 water samples (100–500 ml each) and it can be deployed at up to 5500 m water depth. At ~$40 K the dimensions are 128 cm (H) × 73 cm (W) × 73 cm (L) and it weighs 110 kg in air empty (148 kg when full of water samples) and 57 kg in water. Green Eyes Environmental Observing Systems [8] sells a system called the Aqua Monitor that is a syringe pump system that can collect up to 47 samples up to 1000 ml each and can be deployed at depths up to 4000 m depending on the system configuration. The Aqua Monitor is ~$35 K measuring 62.3 cm (L) × 14.6 cm (dia.), 12.5 kg in air and neutrally buoyant in water without the battery pack and approximately 25 kg with the battery pack. Fluidion [9] sells a coastal sampler for ~$36 K that can be deployed to 100 m depth and take up to 14 500 ml samples using a vacuum piston design and can be programmed to take samples at set time intervals. The coastal sampler is 106.6 cm × 45.7 cm × 30.4 cm and weighs 60 kg in air and close to neutral in water.

The price of these commercially available systems is significantly higher (~$35 K to ~$40 K) than the system we describe here, but a design for deployment in deep water and with high sample/volume capacity comes with increased cost in materials and complexity. There are multiple ways to build a more compact, lighter, and less expensive system than those commercially available. One of the easiest ways to reduce cost is to use less expensive materials for the pressure case housing. Deep water systems require pressure cases fabricated from metals such as stainless steel, aluminum, and titanium, whereas shallow water cases can be made from plastics such as polyvinyl chloride (PVC), Acrylic, Acetyl, and Delrin. Plastics are easier to machine, lighter, and less expensive. A second way to decrease cost is by using readily available off-the-shelf components that can be adapted for a particular design.

Researchers have taken different approaches to develop automatic water samplers. Martin et al. [10] designed a compact water sampler using a small custom built PVC pressure housing 34 cm (H) × 22 cm diameter that uses syringes with springs that are controlled by off-the-shelf electrical components and a microprocessor. Assembly of the various electrical components is required, contributing to the low-cost of the water sampler. While compact and relatively inexpensive, this system does not have flexibility in determining the volume of water collected. The main limitation is that the maximum syringe volume is only 60 ml, which is limited by the size of the pressure housing. To use larger volume syringes, a larger more expensive pressure case housing would be required as well as different springs to actuate the syringes.

Another interesting system is the Sub-surface Automated Sampler (SAS) develop by NOAA [11]. The appeal of the SAS system is the very compact PVC pressure case housing and low cost of materials. Sample size is less of an issue since it uses a peristaltic pump to fill sample bags of various volumes. The small size of the “T” shaped 25 cm (L) × 15 cm (W) pressure housing makes it small, light-weight and easy to deploy, and the sample water has no contact with metal so sample quality and corrosion are not a concern. There are a few limitations with the SAS design. First, only two samples can be taken at a time, after which the system must either be redeployed, or the sample bags must be switched out. In addition, some parts require 3D printing. While the use of two peristaltic pumps is an advantage, 3D printed parts are required to mount these pumps. Finally, while the use of simple electrical components and microprocessor makes the system affordable, the main circuit board requires component assembly.

Albright et al. [12] present an automatic water sampler design that also uses a peristaltic pump and can collect up to 12 samples per deployment with adjustable sample volume based on pump run-time. Similar to the other systems, it uses custom built PVC pressure case housings, although this system requires 3 pressure cases that are linked together with underwater connectors, adding to the complexity and cost of the system. Two of the three PVC pressure housing contain a peristaltic pump, 7 solenoids, and a manifold. The third PVC pressure housing contains the controller and batteries. The major limitation of this system is the size, approximately 80 cm (L) × 50 cm (W) × 45 cm (H) and weight which is approximately 70 kg. Since water comes in contact with stainless steel parts, corrosion and water quality may be problematic.

**Fig. 1:**
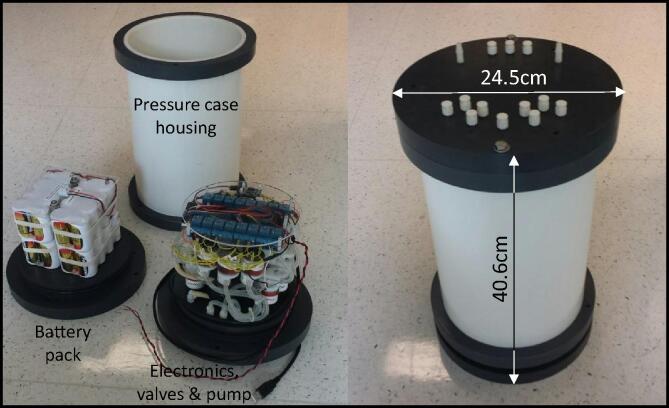
AutoSampler components on the left and assembled system on the right. The AutoSampler is 40.6 cm H × 24.5 cm in diameter, and weighs 10 kg in air and −1 Kg in water.

We designed and built our own AutoSampler because no available systems met our needs. The objective for our design was to incorporate the best features from previously designed systems and make a water sampler that is lightweight, compact, easy to deploy and maintain, is corrosion free, and cost effective to build (Fig. 1). In addition, we wanted our system to be able to fill sample bags of various volumes by controlling pump run-time. We use a single peristaltic pump with pinch valves, silicone tubing, nylon fittings, and a nylon manifold to avoid sample contact with metal and to eliminate corrosion problems. Electronics such as the relay boards, real-time clock, and controller are off-the-shelf components that are inexpensive, readily available, and only require wire jumpers for connections between boards. The Arduino Pro Mini controller is a popular interface that includes free and easy to use software supported by online forums and a large and connected user community. While soldering is required, it is limited to a few wire connections and the installations of a few rectifier diodes to prevent solenoid valve feedback.

We present two versions of open source software to control the AutoSampler. One version is designed for a single deployment (using program: AutoSampler_Discrete_RTC.ino) that can be programmed to begin at a user specified time and discretely sample on a specific date and time for up to 12 samples. The second version is for longer continuous deployments (using program: AutoSampler_Continuous_RTC.ino) where the user will change out sample containers on a set time cycle. For example, the instrument could be programmed to take a sample every 2 h where it will complete the sampling from all 12 ports in 24 h. The user has 2 h from the last sample taken to change out all the containers before the next 24-hour cycle begins. The user can adjust sample volume by changing the amount of time the peristaltic pump is turned on for each sample. The Thomas SR10/50 series peristaltic pump used in this design flows at 220 ml min^−1^. If lower flow rates are needed, the same Thomas SR10/50 series peristaltic pump comes in a 100 ml min^−1^ and 170 ml min^−1^ version that has the same mounting geometry and tubing. Since the software is open source, the user can modify these two programs or completely design their own program to suit their sampling needs.

All submersible equipment requires a pressure case housing to protect electric components. Pressure case housings are often the biggest challenge and expense when designing an underwater system. We opted to custom build our own pressure housing from PVC using machining equipment to mill the various components. The case is rated to 30 m water depth however the peristaltic pump and pinch valves are only rated to 20 m water depth. We provide the drawings for the housing so the reader can have a machine shop mill the components. We designed the electronics, valves, and pumps to be mounted on to a series of stacked acrylic discs that attach to the end cap with 4 aluminum standoffs. This design makes it easy to adapt our system to work in a different pressure case housing from companies such as A.G.O Environmental Electronics, Prevco, Robotic Ocean, and Blue Robotics. Since we use threaded rods for the battery pack, it can also be easily adapted for use in a different pressure case housing.

**Fig. 2:**
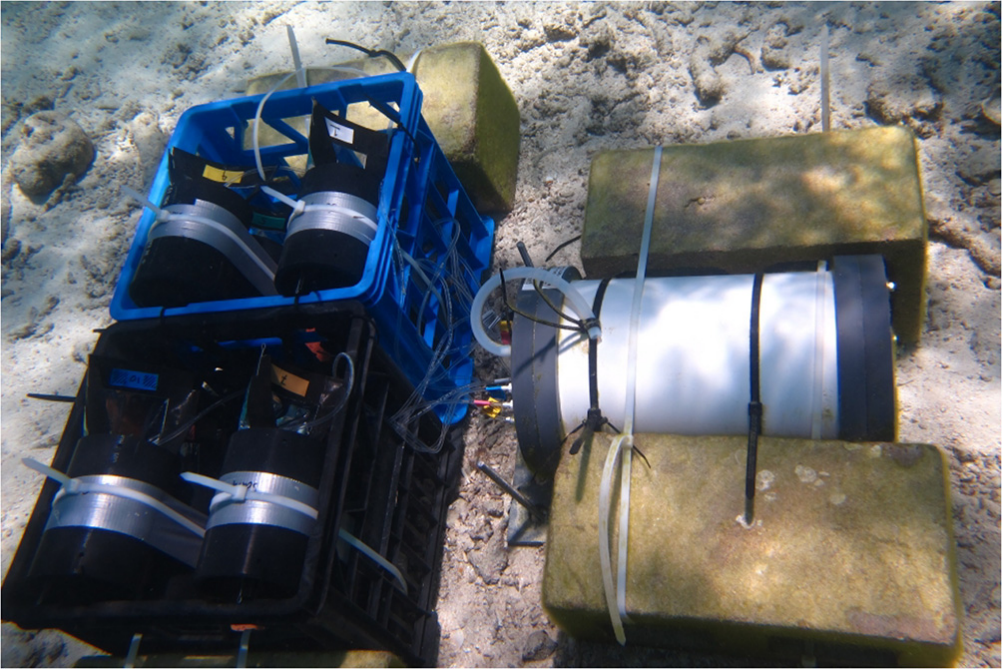
AutoSampler deployed using 500 ml Tedlar™ bags cased in custom PVC cases with 6 bags in each basket to prevent fish from damaging the bags.

The AutoSampler is lightweight (10 kg in air, −1 kg in water) and is compact (40.6 cm H × 24.5 cm diameter), making it easy to deploy off a small boat via SCUBA. The external sample ¼-28 threaded ports use Luer-Lok type quick twist fitting, allowing any size volume syringe to be attached. The same Luer-Lok quick twist barbed fittings can be attached to other sample containers of any size such as bottles or bags (Fig. 2). Depending on the sampling containers used, the footprint of the combined system will vary. As with most custom-built systems, some fabrication and assembly are required. Compared to previously described systems, the AutoSampler is easier to fabricate and maintain because we avoided custom circuit boards and the entire system can be made from off-the-shelf components.

## Hardware description

The AutoSampler is a single underwater pressure housing containing one peristaltic pump, a 6-port nylon manifold, 13 pinch valves, one Arduino Pro Mini controller interfaced with a real-time clock, two 8-channel relay boards, and a 12 VDC battery pack (Fig. 1). With the exception of the custom-built PVC pressure housing, all of the components are off-the-shelf and can be easily fabricated with hand tools.

### Pressure case housing

**Fig. 3:**
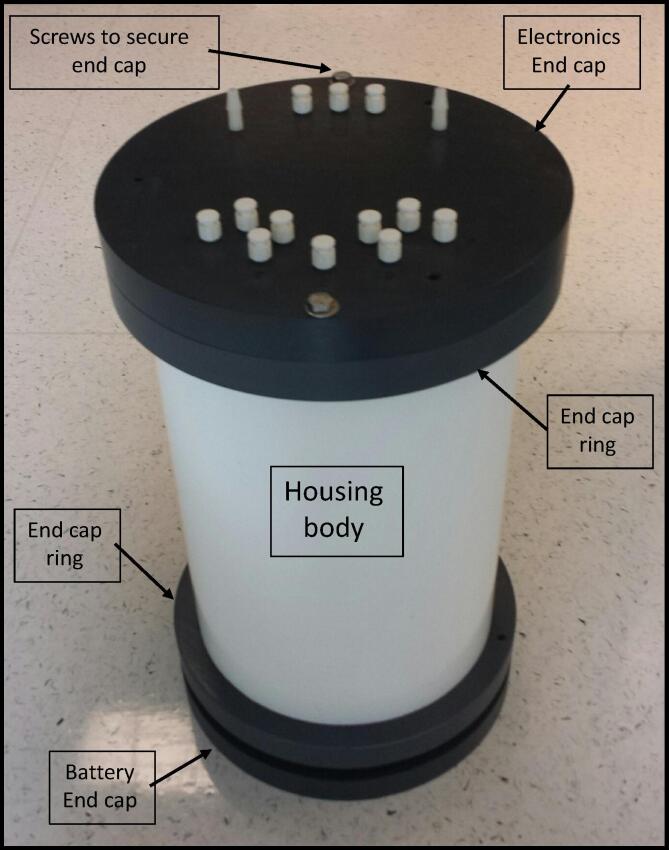
PVC pressure case housing used for the AutoSampler. The housing is comprised of 5 pieces, end caps (n = 2), end cap rings (n = 2), and body.

The pressure case housing is fabricated from PVC although other materials can be used to make a case including acrylic, acetyl, Delrin, titanium, aluminum, and stainless steel. We opted to make our own housing design as described above. The pressure case housing has four components, the body, end cap ring, electronics end cap, and battery pack end cap (Fig. 3).

1. *Body:* The body of the pressure housing is standard wall thickness 8″ PVC pipe. The pipe was cut to 14″ length and milled to true up the ends and chamfered on the inside to make it easier for the o-ring to slide into the housing. The end caps are a piston design where the o-ring on the end cap seals against the inside wall of the body.
2. *End Cap Ring:* This design uses two end cap rings are needed with this design. The purpose of the end cap rings is to provide protection between the end caps and the body of the pressure case housing and to keep the end caps secured to the body. The end cap rings were fabricated from 12″ x 12″ x 1″ thick PVC flat stock and both are milled to make 10″ OD × 8.655″ ID × 1.0″ thick disks. Both cap rings are identical in design. The two 0.2031″ diameter holes in the end cap ring are tapped for ¼-20 threads. PVC cement is used to fix the end cap ring to the body.
3. *Electronics End Cap:* This end cap is machined from 12″ x 12″ x 3″ thick PVC flat stock and milled to make 10″ OD × 2.125″ thick disks with the faces and sides parallel and perpendicular. The piston portion of the end cap is machined to 7.981″ OD × 1.125″ thick to fit into the ID of the pressure case housing body. An o-ring groove is cut into the side of the piston to accept a Viton o-ring. Two 0.2656″ (17/64″ drill bit) diameter holes are drilled to match the tapped holes in the end cap ring. Three 0.2031″ (13/64″ drill bit) diameter holes are drilled and then tapped for ¼-20 threads for jack bolts (Fig. 4). The jack bolts, which are the same bolts used to secure the end cap to the end cap ring, are used to separate the end cap from the body as the jack bolts holes line up with the center of the rim on the pressure case housing. Fourteen additional 0.21875″ (7/32″ drill bit) diameter holes are required for the ¼-28 UNF threaded Luer-Lok quick twist fittings. Two of these ¼-28UNF threaded ports receive barbed fittings for the intake and out take fittings. The other 12 ports are for connecting sample containers. All 14 of these holes extend from the outer face of the end cap through to the face of the piston. On the face of the piston, four 0.159″ (5/32″ drill bit) diameter holes are drilled and then tapped for 10–32 threads to accept aluminum standoffs to support the electronics mounting plate.

**Fig. 4:**
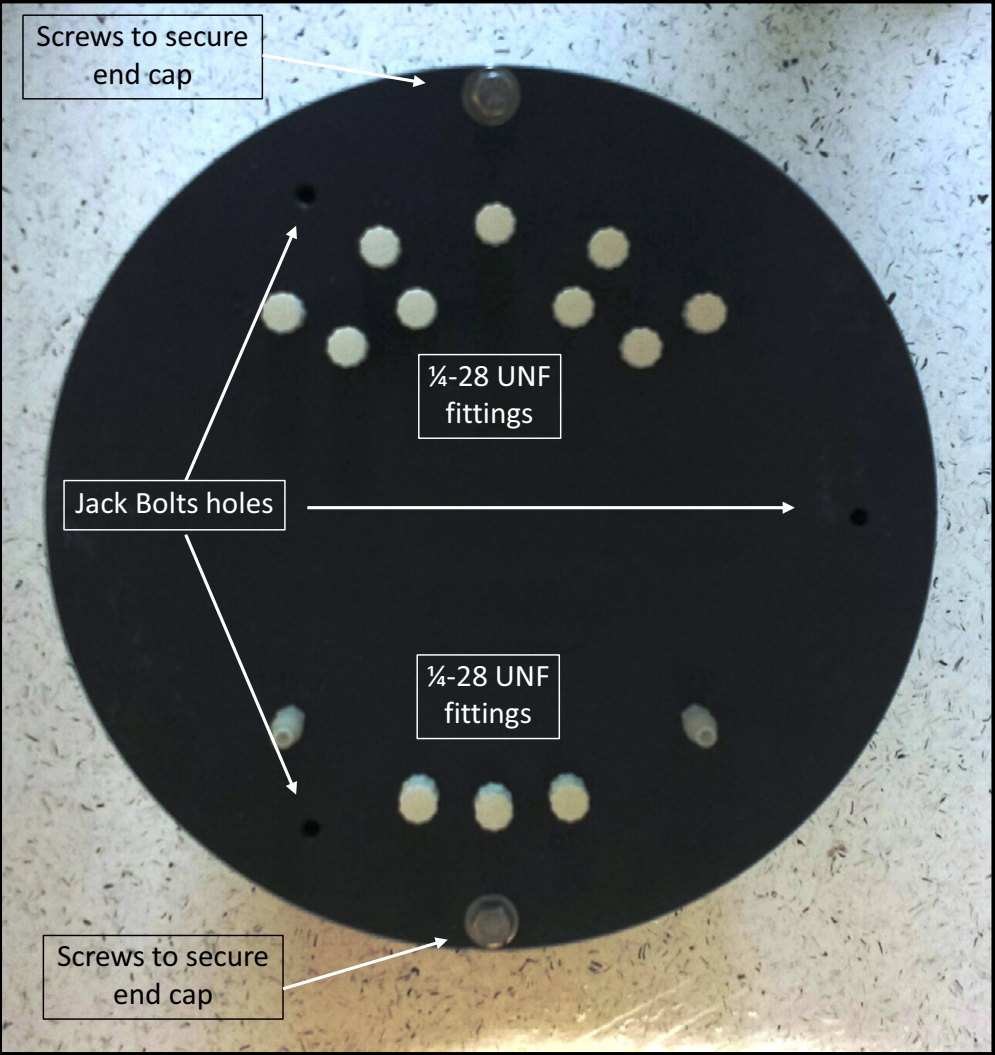
Electronics end cap for the AutoSampler. The end cap is drilled and tapped for three ¼-20 jack screws and for fourteen ¼-28UNF fittings.
4. *Battery Pack End Cap:* This end cap is very similar to the Electronics End Cap with three ¼-20 jack bolts threaded holes, and two securing bolts holes. The end cap also has one 0.422″ (27/64″ drill bit) diameter hole tapped for ½-20 threads for a pressure release plug (McMaster PN: 51205 K288). On the face of the piston, four 0.2031″ (13/64″ drill bit) diameter holes are drilled and tapped for ¼-20 threads to receive stainless steel threaded rods to secure the battery pack (Fig. 5).

**Fig. 5:**
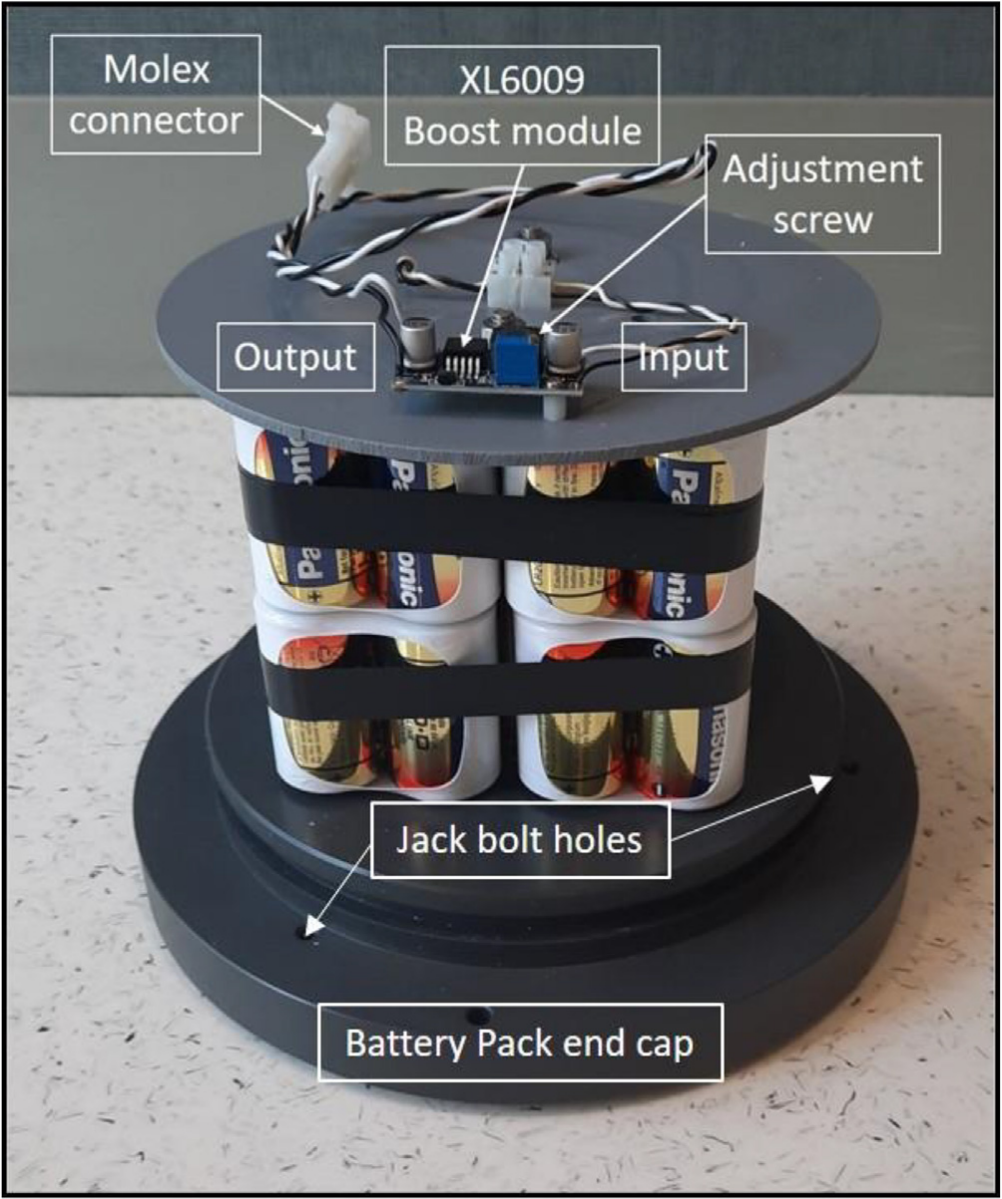
Battery Pack end cap for the AutoSampler. The end cap is drilled and tapped for three ¼-20 jack screws, two securing bolt holes, one ½-20 threaded port for a pressure release plug, and four ¼-20 threaded holes for battery pack support rods. The top plate shows the location of the XL6009 boost module and the adjustment screw.

### Electronics mounting plate

**Fig. 6:**
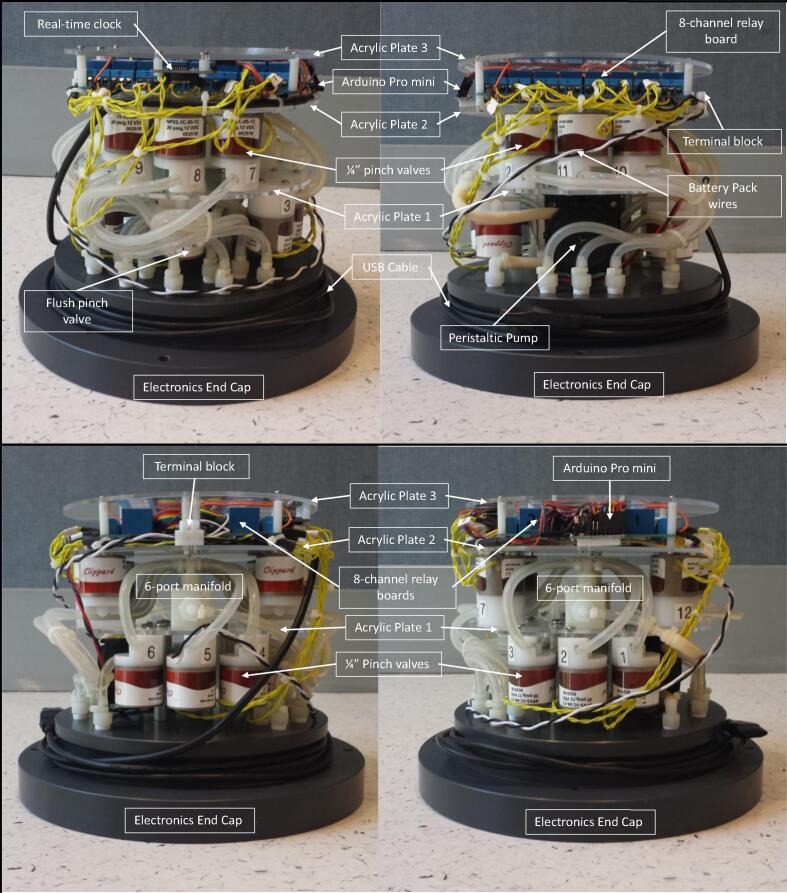
Electronics mounting plates (90° side views) for the AutoSampler illustrating the locations of the Arduino Pro Mini, real-time clock, two 8-channel relay boards, terminal block, and the USB cable for communication with the Arduino Pro Mini controller. Note that the side view images are 90° counterclockwise rotations beginning in sequence from the top left to bottom left to top right to bottom right.

**Fig. 7:**
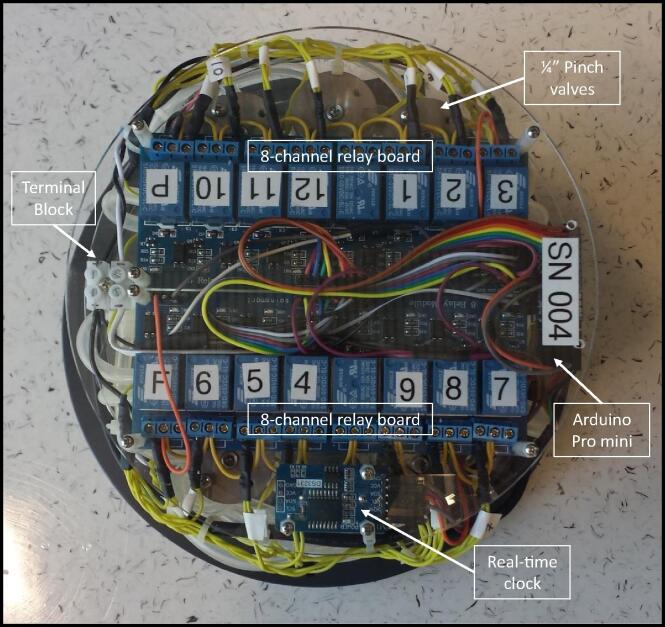
Electronics mounting plates (top view of stack) for the AutoSampler showing the Arduino Pro Mini, real-time clock, two 8-channel relay boards, terminal block, and a USB cable for communication with the Arduino Pro Mini controller.

The electronic mounting plates are made from three 7″ OD × 1/8″ thick clear laser cut acrylic from Delvies Plastics. Acrylic disc 1 supports the 220 ml min^−1^ peristaltic pump, ¼″ flush pinch valve, twelve ¼″ sample pinch valves, and a 6-port nylon manifold (Fig. 6). Acrylic disc 2 supports the two 8-channel relay boards, and Arduio Pro Mini controller. Acrylic disc 3 supports the real-time clock and acts as a cover plate for the electronics package (Fig. 7).

### Battery pack

The battery pack consists of four individually shrink-wrapped 12 VDC battery packs comprised of 8 alkaline D-cell batteries with approximately 15 Ah/pack (Fig. 5). The four individual battery packs are linked together in parallel to provide 12 VDC and 60 Ah. These power specifications can be achieved by using of-the-shelf D-cell battery holders that are linked together in parallel that accept individual D-cell batteries. Other battery combinations and types can be used to provide power to the internal electronics and pumps to suit the user’s power requirements. The Arduino Pro Mini accepts 5 VDC to 12 VDC input and regulates 5 VDC to the logic portion of the two 8-channel relay boards and the real-time clock. The full 12 VDC powers the 8-channel relays, the 13 pinch valves and the peristaltic pump. The estimated power consumption of the electronics (Arduino Pro Mini, real-time clock, and relay boards) is ~0.110 A, each Clippard pinch valve has a factory specification of 0.400 A, and the Thomas 10/50 peristaltic pump has a factory specification of 0.540 A for a total of 1.040 A. This amounts to ~0.017 Ah min^−1^ of operation. To calculate the total deployment Ah, the following equation can be used as an estimate.

**Table:** 

Logic and electronics (controller, real-time clock, relays)	0.110 A
Clippard pinch valve	0.400 A
Thomas 10/50 peristaltic pump	0.540 A
Total energy consumption	1.040 A (0.017 Ah min^−1^)

Deployment Ah = ((0.017 * sampling time in minutes) * (number of samples * number of cycles).

*Deployment Example*: A deployment using 2 min pumping time, taking 12 samples day^−1^, deployed for 15 days will have a total of 180 sample cycles consuming ~ 6 Ah or about 10% of the total 60 Ah battery pack capacity. This would amount to ~1.2 VDC in battery voltage. A DC to DC boost converter/regulator module (XL6009) can be used on the battery pack to provide a steady 12 VDC output to the electronics and pump as the battery pack power decreases. With this module the input voltage can drop to ~5 VDC and still maintain the set 12 VDC output allowing for 58% (~35 Ah of the total 60 Ah) of the battery pack available to power the AutoSampler.

Example: Deployment Ah = ((0.017 * 2 min) * (12 samples * 15 cycles).
Deployment Ah = 6.12

The Thomas 10/50 peristaltic pump specifications states that the Novoprene tubing has 500 hr. lifetime and the DC motor 1000 hr. This amounts to approximately 20 days of continuous operation before the pump tubing needs to be replaced but should be taken into account prior to deployment.

## Design and software files

Design and software files for the AutoSampler are available for download from the Open Science Framework. The file **AutoSampler Build Instruction Manual.pdf** (https://doi.org/10.17605/OSF.IO/E7NJM) contains build instructions and all of the drawings and schematic diagrams to build the AutoSampler. The file **AutoSampler Drawings and Schematics.pdf** (https://doi.org/10.17605/OSF.IO/E7NJM) contains a summary of the all the drawings and schematics required to build the AutoSampler.

The AutoSampler software was coded using the Arduino IDE platform that is available via download from https://www.arduino.cc/en/Main/Software. Direct links to download the AutoSampler code are provided in the table below.

**Table:** 

**Design and program file names**	**File type**	**Open source license**	**Location of the file**
AutoSampler Build of Materials	pdf	GNU General Public License (GPL) 3.0	(https://doi.org/10.17605/OSF.IO/E7NJM)
AutoSampler Build Instruction Manual	pdf	GNU General Public License (GPL) 3.0	(https://doi.org/10.17605/OSF.IO/E7NJM)
AutoSampler User Materials	pdf	GNU General Public License (GPL) 3.0	(https://doi.org/10.17605/OSF.IO/E7NJM)
AutoSampler Drawings and Schematics	pdf	GNU General Public License (GPL) 3.0	(https://doi.org/10.17605/OSF.IO/E7NJM)
time_set_manually (A)	ino	GNU General Public License (GPL) 3.0	(https://doi.org/10.17605/OSF.IO/E7NJM)
current_time_LCD (B)	ino	GNU General Public License (GPL) 3.0	(https://doi.org/10.17605/OSF.IO/E7NJM)
AutoSampler_Continuous_RTC (C)	ino	GNU General Public License (GPL) 3.0	(https://doi.org/10.17605/OSF.IO/E7NJM)
AutoSampler_Discrete_RTC (D)	ino	GNU General Public License (GPL) 3.0	(https://doi.org/10.17605/OSF.IO/E7NJM)

Program descriptions:

(A). **time_set_manually** – Used to set the real-time clock.
(B). **current_time** – Used to display the time from the real-time clock on computer and LCD.
(C). **AutoSampler_Continuous_RTC** – Used to run the autonomous underwater multiport water sampling system after the real-time clock is set to run in continuous sampling mode.
(D). **AutoSampler_Discrete_RTC** – Used to run the autonomous underwater multiport water sampling system after the real-time clock is set to run in single deployment discrete sampling mode.

## Bill of materials

A bill of materials list for the AutoSampler is available for download from the Open Science Framework in pdf format under file filename **AutoSampler Build of Materials.pdf** (https://doi.org/10.17605/OSF.IO/E7NJM). Most of the items in the bill of materials list can be acquired from multiple suppliers. In some cases, it was less expensive to buy some components in bulk (e.g. stainless-steel fasteners, PVC pipe) than the exact number of items from a local hardware store.

## Build instructions

A separate Build Instruction document for the AutoSampler is available for download from the Open Science Framework in pdf format under file filename **AutoSampler Instruction Manual.pdf** (https://doi.org/10.17605/OSF.IO/E7NJM). This manual includes the step-by-step assembly and wiring instructions for all components and the tools needed to assemble the system.

## Operation instructions

The user manual document for the AutoSampler is available for download from the Open Science Framework in pdf format under file filename **AutoSampler Users Manual.pdf** (https://doi.org/10.17605/OSF.IO/E7NJM). This manual provides the step-by-step operations of the entire system.

## Validation and characterization

**Fig. 8:**
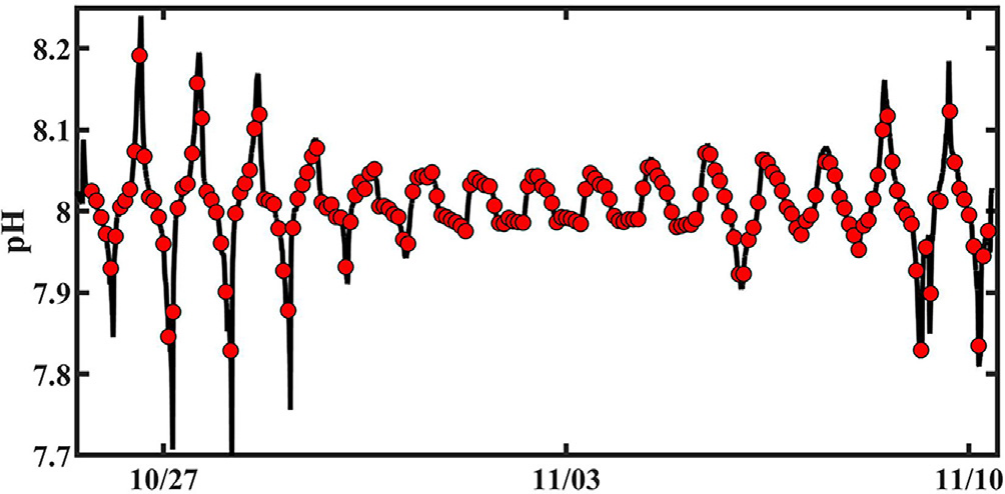
pH from the SeapHOx sensor (black line) taken every 10 s and discrete samples (red circles) collected by the AutoSampler at 2 h intervals in 2019. This is a subset of the SeapHOx pH data presented in Bresnahan et al. [13] (For interpretation of the references to colour in this figure legend, the reader is referred to the web version of this article.)

AutoSamplers were deployed on the south coral reef flat adjacent to Lizard Island, located in the northern part of the Great Barrier Reef on two field campaigns in support of the work published by Bresnahan et al. [13]. Two AutoSamplers were deployed from Oct 17 to Oct 31, 2018 and one unit was deployed Oct 26 to Nov10, 2019, but for validation purposes we will only use the results from the 2019 deployment. For both years, the AutoSamplers were programmed to collect discrete samples every 2 h throughout the deployment. Mean water depth ranged from 1.0 to 1.8 m, depending on the site. An example of the AutoSampler deployment configuration at Lizard Island is shown in Fig. 2. Approximately 500 ml of seawater was pumped into Tedlar™ bags (Zefon, EG-ZP-05) for each sample. The bags were pre-poisoned using mercuric chloride to prevent biological activity from altering seawater chemistry after collection [14]. Sample bags were replaced daily to obtain samples every 2 h throughout the deployment and analyzed for pH [15], [16]. For validation, an in-situ pH sensor, a SeapHOx [13], [17], was deployed next to the AutoSampler to test for any potential biases from the collected samples. The AutoSampler successfully collected discrete samples throughout both deployments as scheduled. When the time stamp from the pH data obtained from the AutoSampler was merged with the continuous pH data from the SeapHOx, there is excellent agreement (Fig. 8). In this example, the discrete pH data (red dots) is plotted with the same time stamp as the pH data from the SeapHOx (black line). Here the two pH measurements agreed well over a large diel cycle that ranged from 7.7 to 8.2, which was driven by reef metabolism (production/respiration, and calcification/dissolution). Obviously the SeapHOx high resolution record is more accurate in representing the pH at this site however, what this data also demonstrates is the Autosampler’s capability for accurately collecting and storing seawater samples for at least 24 h, making it a useful tool to measure, for example, nearshore metabolic processes in a fashion that resolves diel cycles over many weeks.

During the two field experiments the AutoSampler was deployed for 15 and 16 days on one battery pack with minimal (~1 VDC) drop in voltage. On longer deployments decreasing battery voltage will impact pumping speed and thereby affect sample container fill-time. To address this issue, we added a voltage regulator/booster module XL6009 as outlined in #1 below and incorporated it into this design. In addition, the AutoSampler also encountered a few problems during these two field experiments that were resolved and are outlined in 2 through 5 below. Item 6 provides some insight for expansion and modification.

1. The AutoSampler in the experiment above was deployed without a DC to DC voltage regulator/boost converter module XL6009 however, it has been incorporated into this design and it has been laboratory tested. The benefit of this module is that it will accept 5 to 30 VDC input and can be set to provide a consistent regulated 12 VDC output within this voltage input range. We recommend the addition of this module to the battery pack so that the pumping specifications can be maintained and extend deployment duration by maximizing the battery pack voltage. We provide the link information on how to install and use the XL6009 regulator/boost module in the **Build** **instructions** and **Operation** **instructions** sections sections above.
2. The pump ingested some carbonate particles that clogged the lines leading to a leak inside the housing. To prevent particles from entering the pumping system a mesh screen (e.g. 250 µm) should be placed over the AutoSampler intake port (Fig. 9). This will filter large particles that can easily clog the plumbing system. Fine particles can also be problematic making deployment in high energy environments difficult. The use of in-line filters, using a finer mesh is, not recommended as they can easily clog and impact pumping performance, but as a tradeoff it will prevent the lines from clogging.

**Fig. 9:**
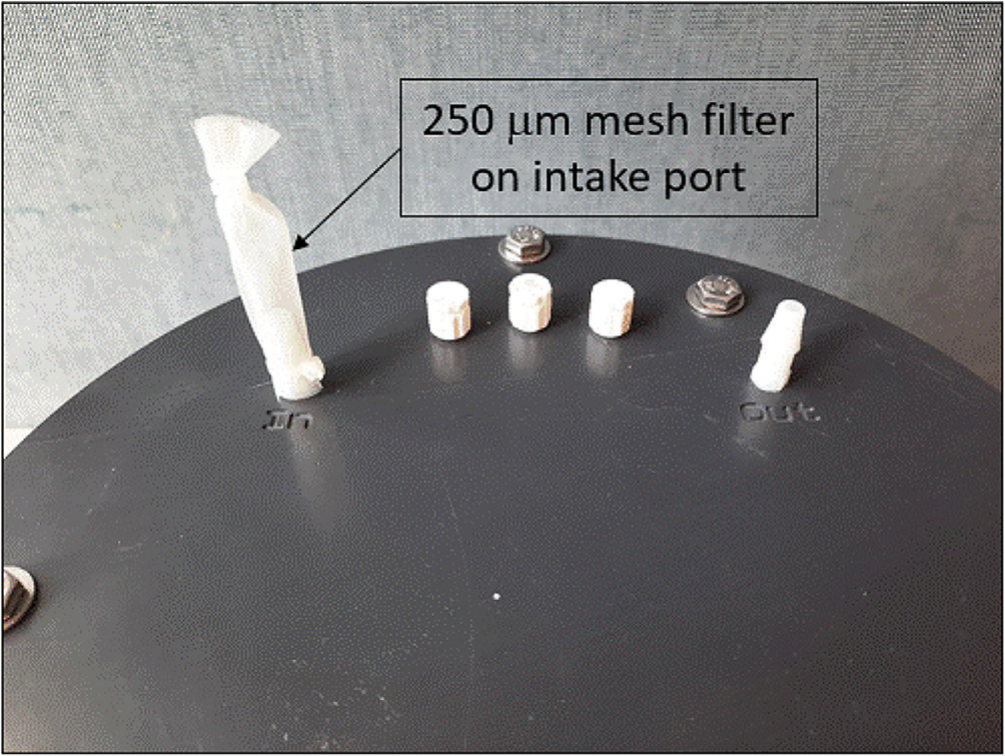
Shows the installation of a 250 μm mesh filter on the AutoSampler intake port to minimized clogging.
3. Internal leaks from clogging can cause damage to the electronics inside the pressure case housing. For safety, the end cap is equipped with a pressure release plug to release any excess pressure in the housing in the event of flooding.
4. As mentioned, the AutoSampler can use different water collection devices (e.g. syringes, bags, bottles). The use of bags adds a challenge because fish like to graze on the bags and can make holes. Also wave motion can cause bags to chafe and produce holes or become entangle. During the deployment we used milk crates and PVC tubes as shown in Fig. 2 to protect the bags.
5. Care must be taken if deploying the AutoSampler in a high energy environment as the sample containers and tubes may be entangled or damaged and the intake and lines could become clogged.
6. For long-term deployments it would be useful to record each sample sequence. To accommodate the addition of a mini SD card reader more GPIOs are required or less valves/ports used to make 4 additional GPIOs available. Mucciarone et al. [18] incorporated a mini SD card reader into their AutoPump design that used the same Arduino Pro Mini controller, similar real-time clock, and relay board which can be used as an example. GPIO pins 10, 11, 12, and 13 are reserved for the mini SD reader. The code and hardware we provide here can be modified to accommodate this feature, but only 8 of the 12 ports could be used. When we developed the AutoSampler, our goal was to maximize the number of ports for our project which required the use of all 18 GPIOs on the Arduino Pro Mini controller. As a result, this left no room for expansion or the possibility of adding a mini SD card reader. We are now working with the Arduino Nano and Nano Every which will provide 4 extra GPIOs to accommodate a mini SD card reader. We have also experimented using small flow rate peristaltic pumps like the Thomas 10/30 pump series. The peristaltic tubing inside diameter is smaller so it requires different tubing adapters, but it is an easy modification. With the smaller peristaltic tubing, clogging is a greater concern, so care must be taken to deploy the system in a suitable environment.

We have designed and tested a compact and inexpensive AutoSampler that is largely fabricated using off-the-shelf parts making it easier to build and maintain. The AutoSampler represents an advance over many existing commercial and custom designs by being lightweight, compact, easy to deploy, maintain, it is corrosion free, and cost-effective to build. The AutoSampler was field-tested in a shallow coral reef environment and is capable of reliably collecting seawater for chemical analyses. The development of this AutoSampler should help facilitate research that would otherwise be too labor-intensive or logistically difficult to conduct, especially in remote locations.

## Declaration of Competing Interest

The authors declare that they have no known competing financial interests or personal relationships that could have appeared to influence the work reported in this paper.
