# Supplementary material for: Autonomous submersible multiport water sampler
Source: HardwareX. 2021 Apr 22;9:e00197. doi: 10.1016/j.ohx.2021.e00197 (PMC9041238; doi:10.1016/j.ohx.2021.e00197)
Supplement: Supplementary data 1 [file mmc1.pdf]

```
// Date and time functions using a RX8025 RTC connected via I2C and Wire lib
// To set time, click on the upload arrow 10 seconds before set time below

#include <Wire.h>
#include "Sodaq_DS3231.h"

char weekDay[][4] = {"Sun", "Mon", "Tue", "Wed", "Thu", "Fri", "Sat" };

//year, month, date, hour, min, sec and week-day
//(starts from 0 and goes to 6, Sunday=0, Saturday=6)
//writing any non-existent time-data may interfere with normal
//operation of the RTC.
//Take care of week-day also

DateTime dt(2020, 2, 9, 14, 10, 0, 0);


void setup () {
	Serial.begin(9600);
	Wire.begin();
	rtc.begin();
	rtc.setDateTime(dt); //Adjust date-time as defined 'dt' above
}

void loop () {
}
```
