# Supplementary material for: Autonomous submersible multiport water sampler
Source: HardwareX. 2021 Apr 22;9:e00197. doi: 10.1016/j.ohx.2021.e00197 (PMC9041238; doi:10.1016/j.ohx.2021.e00197)
Supplement: Supplementary data 2 [file mmc2.pdf]

STANFORD UNDERWATER LABORATORY

MG1-V2

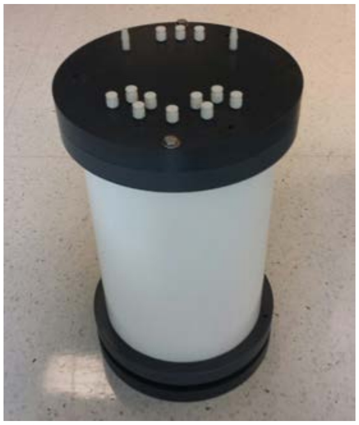

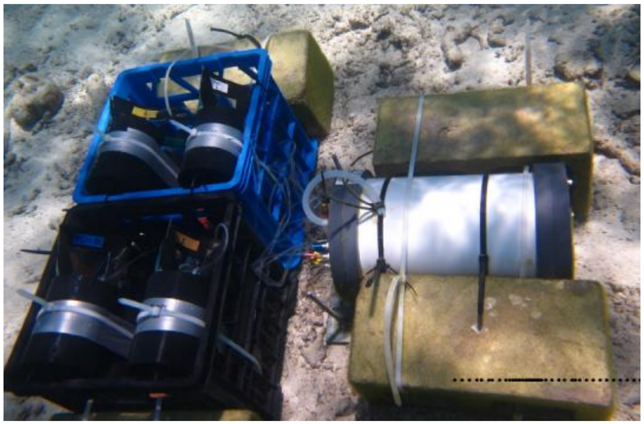

Autonomous Multiport Water Sampler (AutoSampler) User Manual

David A. Mucciarone, Hans B. DeJong, and Robert B. Dunbar (Stanford University), Yui Takeshita (Monterey Bay Aquarium Institute), Rebecca Albright (California Academy of Science), Keaton Mertz (Monterey Bay Aquarium Institute)

Refer to the Bill of Materials spreadsheet as part numbers are used to explain how the components are connected. Basic soldering, wire stripping, and basic mechanical techniques are required to assemble this system. The system is composed of mostly “off-the-shelf” components. The wires can be neatly bundled and controlled using cable ties (available at many hardware store locations) along with cable tie and ribbon cable adhesive mounts.

Several tools will be required to fabricate this system.

1. Drill press or hand drill
2. Drill bit 3/32, 7/64”, 1/8”, 5/32, 13/64”, 7/32’, 1/4”, 17/64”, 27/64”
3. Rectangular 6” fine file or similar
4. Wire crimper/stripper/cutter multi purpose tool or similar
5. Small and medium sized Philips screwdriver #0, #1, #2
6. Wrench 1/4”, 5/16”, 7/16”

7. Heat gun for heat shrink tubing
8. Soldering iron and solder
9. Digital multi-meter
10. Personal protection equipment (PPE)– gloves, eye protection, N95 mask

Caution: When working with plastics it is important to wear the proper PPE to avoid personal harm and harm to the environment. Make sure to follow all Environmental Health and Safety regulations when handling and disposing of materials.

Autonomous Multiport Water Sampler System (AutoSampler): This system is comprised of an underwater pressure case housing, a peristaltic pump, ¼" solenoid pinch valves, Arduino Pro Mini controller interfaced with a real-time clock, two 8-channel relay boards, and a battery pack. With the exception of the custom-built underwater housing all of the remaining components are off-the-shelf and can be easily constructed with simple hand tools.

A. Pressure Case Housing: The pressure housing is fabricated from PVC using machining equipment. This section will only describe the pressure case housing components not how to actually machine those components. These components include the end caps, end cap rings, and pressure case housing body. Both of our end caps were Computer numerical control (CNC) machined.

1. *End Caps:* Both end caps are machined from 12" x 12" x 3" thick PVC flat stock (McMaster PN: 8747K401) and both are milled to make 10" OD x 2.125" thick discs. Both end caps are very similar in design. The top end cap is for the electronics and the bottom end cap is for mounting the battery pack. Steps 1a-c are identical for both the top and bottom end caps. The two end caps have a few differences in the number and location of tapped holes. Step 1d will focus on the top end cap and Step 1e will focus on the bottom end cap. In addition, Section 1B will focus on the electronics top end cap and the placement of the tapped holes for the sample ports.

   a. Each end cap (top and bottom) is machined to make a 7.981" diameter x 1.125" deep piston shape with a 0.230" deep x 0.643" wide groove cut into the piston for the sealing o-ring (Figure 1).

   b. Two 0.2656" (17/64") diameter holes are drilled 180° from each other on both end caps. These holes are 0.34375" (based on the center of the holes) from the outer edge of the rim. These holes will be used to secure the end cap to the cap rings (Figure 1 blue rectangles, Figure 2, blue circles).

   c. Three 0.2031" (13/64") diameter holes are drilled 120° from each other on center (Figure 2, blue dashed lines) and in 0.865 from the outer edge of the end rim on both end caps. These holes are tapped for ¼-20 bolts that well be used as "jack" bolts and are lined up with the center of the housing body rim (Figure 1 green rectangles, Figure 2 green circles).

   d. On the top end cap only, four 0.1590" (5/32", Figure 3 left, green circles) diameter holes are drilled and tapped for 10-32 threads on the top end cap piston for the installation of

aluminum standoffs.

e. On the bottom end cap only, one 0.422” (27/64”) diameter hole is drilled and tapped for ½-20 threads. This single hole is for the pressure release plug (Figure 3, right). Four 0.2031” (13/64”, Figure 3 right, green circles) are drilled and tapped for ¼-20 threads on the bottom end cap piston for stainless steel threaded rods used to secure the battery pack.

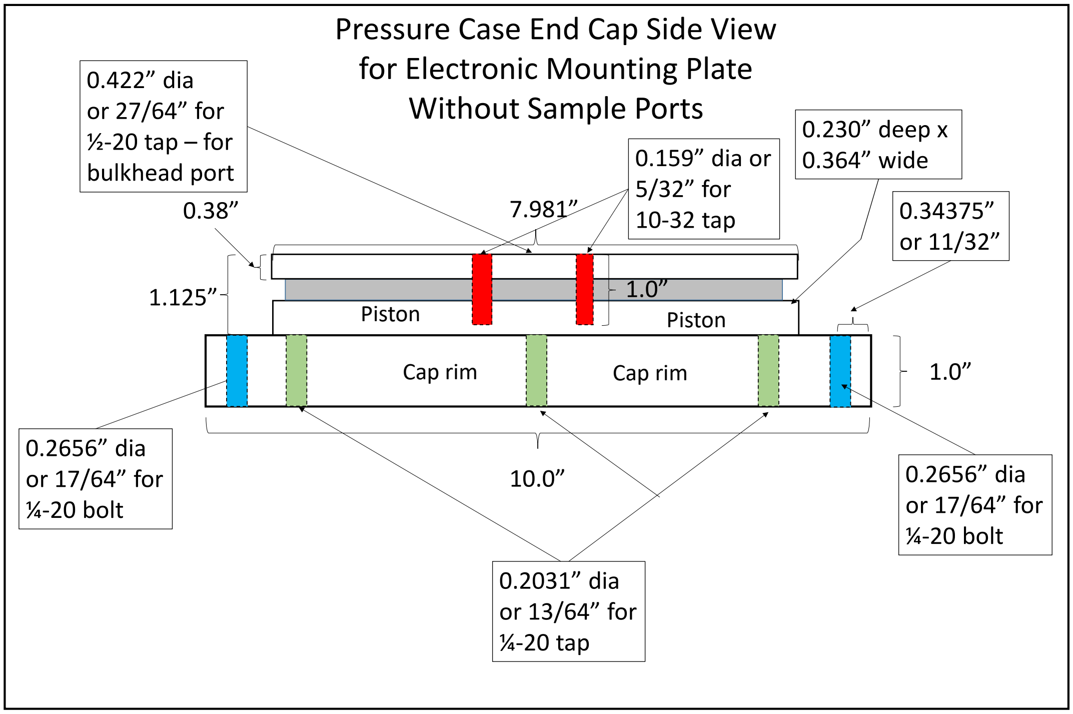

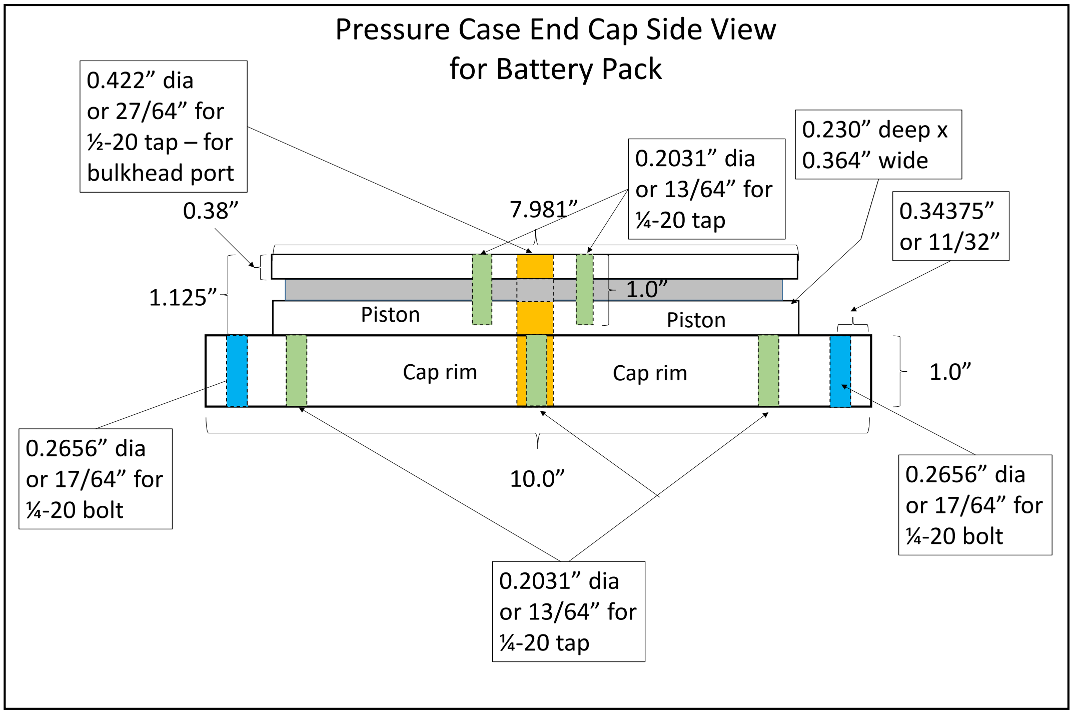

Figure 1: Electronics end cap side view (top image) on underwater pressure case housing not showing the sample ports. Blue rectangles represent 0.2656” diameter drilled (not tapped) holes for ¼-20 bolts, green rectangles are 0.2031” (13/64”) diameter drilled holes that are tapped for ¼-20 bolts, red rectangles are drilled 0.159” (5/32”) diameter and tapped for 10-32 standoffs. On the battery end cap (bottom image), the blue and green rectangles have the same specifications as on the top image. Note the addition of the yellow triangle is 0.422” (27/64”) diameter drilled hole tapped for ½-20 bulkhead connectors for the purge plug. The Battery Pack end caps (bottom) is slightly different and has four ¼-20 tapped holes on the piston instead of 10-32 tapped holes.

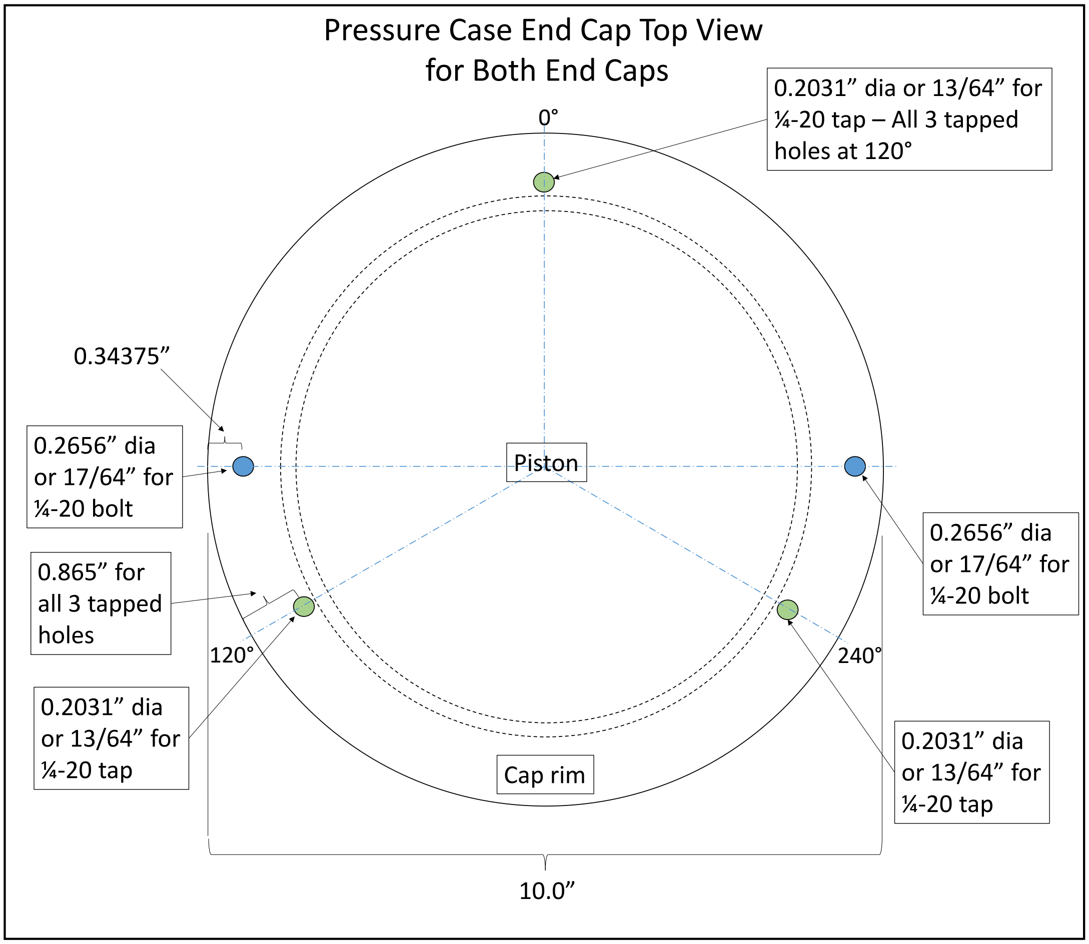

Figure 2: End cap top view on underwater pressure case housing for the electronics mounting plate not showing sample ports or purge port locations. Blue circles represent 0.2656” (17/64”) diameter drilled holes are for ¼-20 bolts and green circles are 0.2031” (13/64”) diameter drilled holes and are tapped for ¼-20 bolts.

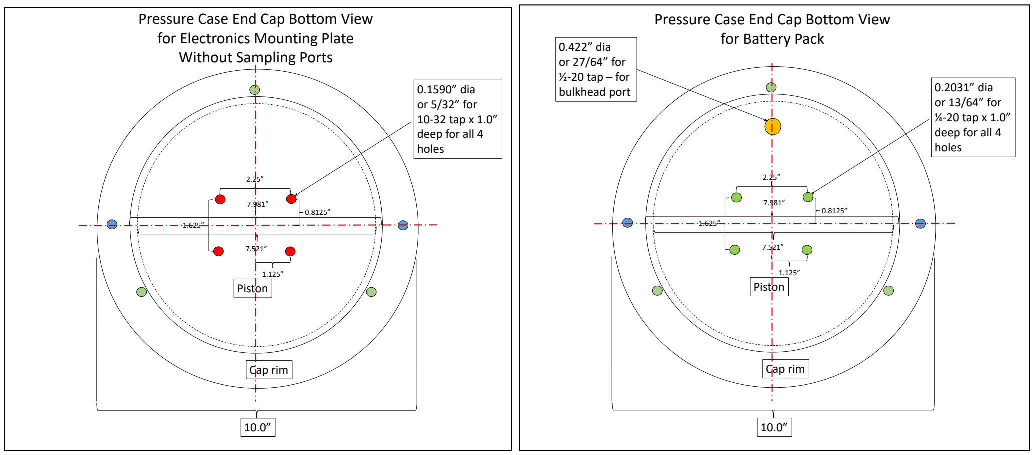

Figure 3: End cap bottom views on underwater pressure case housing for the electronics mounting plate (left) and the battery pack (right). Red circles are 0.159” (5/32”) diameter drilled and tapped to 10-32 for aluminum standoffs (left). Green circles are 0.2031” (13/64”) diameter drilled holes and are tapped for ¼-20 threads for ¼-20 stainless steel threaded rods to support the battery pack (right). The yellow circles are 0.422” (27/64”) diameter holes tapped for ½-20 threads for the two bulkhead connectors (left) and the pressure release plug (right). The red dashed lines indicate the center lines on the end cap.

2. *End Cap Ring:* Both cap rings are machined from 12” x 12” x 1” thick PVC flat stock (McMaster PN: 8747K118) and both are milled to make 10” OD x 1.0” thick disks with the faces and sides parallel and perpendicular with an 8.655” ID. The ID (8.655”) of the end cap ring is slightly larger than the OD (8.625”) of the pressure case housing body to make room for the adhesive and for fitment. Both cap rings are identical in design (Figure4, blue rectangles and circles, respectively). The two 0.2031” (13/64”) diameter holes in the cap ring are tapped to for ¼-20 threads and match up with the blue rectangles in Figure 1 and the blue circles in Figure 2 and 3.

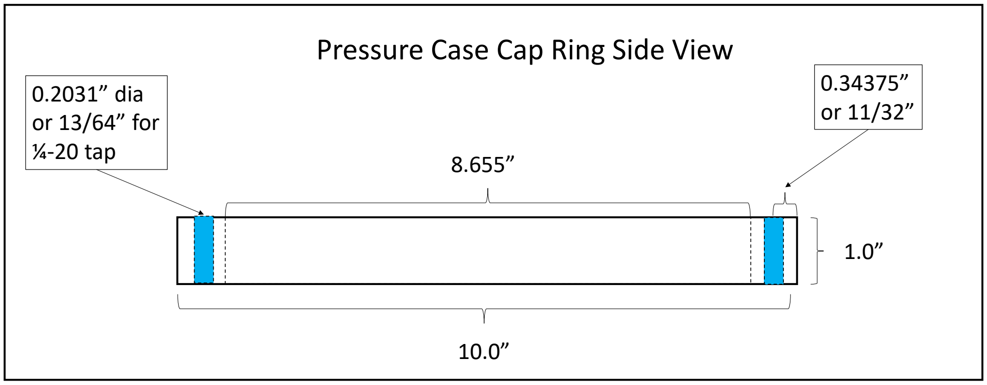

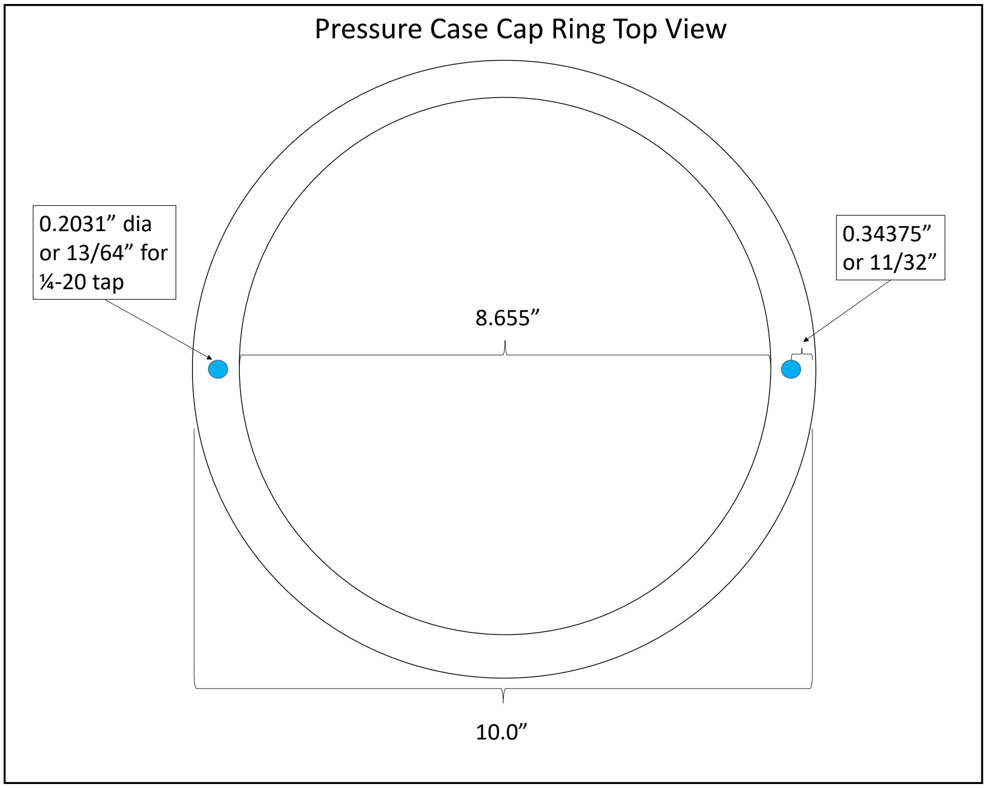

Figure 4: Side view of the pressure case housing cap ring (top image) and top view (bottom image) show the locations of the 0.2031” (13/64”) diameter holes tapped for ¼-20 threads.

3. *Body:* The body of the pressure housing is standard wall 8” PVC pipe (McMaster PN: 48925K26). The pipe was cut to 14” length and milled to true up the ends and chamfered on the inside to make it easier for the o-ring to slide into the housing (Figure 5).

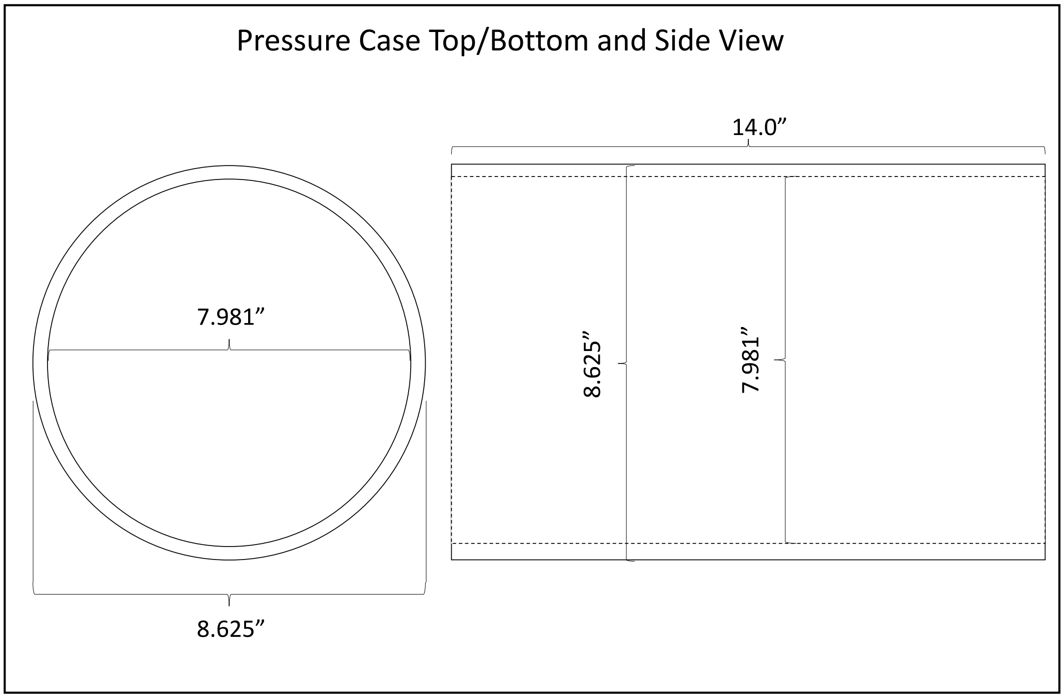

Figure 5: Body of 8” PVC pressure case housing. Top and bottom view on the left and side view on the right.

4. *Assembly:* The end cap rings need to be attached to the pressure case housing body. It is best to do this one end at a time.

   a. Clean the outer end of the pressure case housing body (approximately 1” down from the rim) and the inside of the end cap ring using PVC clear cleaner (e.g. McMaster PN: 74605A44). Make sure to follow instructions on the container for preparation/drying time.

   b. Prime the outer end of the pressure case housing body (approximately 1” down from the rim) and the inside of the end cap ring using PVC clear primer (e.g. McMaster PN: 18815K51) similar to Step 4a above. Make sure to follow instructions on the container.

   c. Apply PVC cement (e.g. McMaster PN: 74605A15) to the outer end of the pressure case housing body approximately 1” down from the rim and also to the inside of the end cap ring. Slide the end cap ring onto the end of the pressure case housing until the top of the ring is flush with the rim of the pressure case housing body. Make sure to follow instructions on the container for drying time.

   d. Repeat Steps 4a-c for attaching the other end cap ring.

B. Electronics End Cap and Sample Port Locations: As discussed above in Section 1A and displayed in Figure 1 (Top) and Figure 3 (Left), the end cap drawings for the electronics mounting plates did not show the location of the sample ports. This section will discuss the locations of the fourteen feed-through samples ports. All sample ports are drilled and tapped for 1/8”-27 NPT threads on the piston (1.125” deep) and for ¼-28 UNF on the cap rim (1.0” deep) to accommodate the different fittings on both ends (Figure 6, yellow and black rectangles).

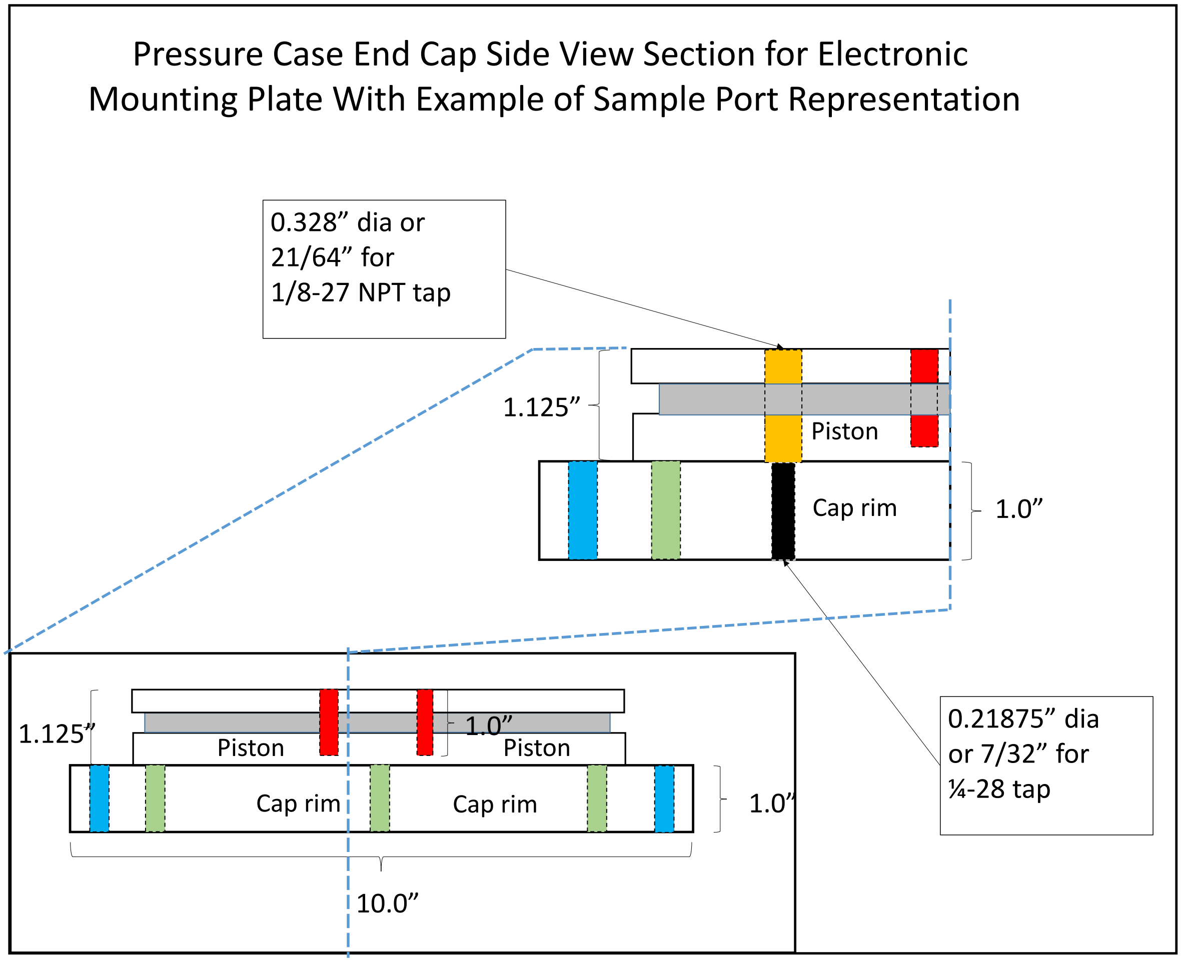

Figure 6: Representation of the end cap feed-through sample ports on the electronics mounting plate end cap. The blue dashed lines show an expanded view. Note that only one example (yellow and black rectangles) out of all 14 is provided to illustrate how the inner and outer holes are connected.

1. Locate the center of the end cap on the top side and make a horizontal and perpendicular vertical reference line through the center point. The horizontal line will be the 0° and 180° reference and the vertical will be the 90° and 270° reference.

2. Using the center point, draw 2 circles (with a radius of 2.5” and a 3.25”, respectively). These two reference circles will set the arcs for the 14 ports (Figure 7).

3. Using the information in Figure 7, make reference lines at angles indicated in the drawing. The intersections between the arcs and the reference lines will mark the locations of each port.

4. Use 0.21875” (7/32”) drill bit to drill completely through the end cap at all 14 locations as illustrated in Figure 7 (black circles).

5. Use a ¼-28 tap and thread down from the top of the cap rim (piston facing down) only 1.0” for each of the 14 holes.

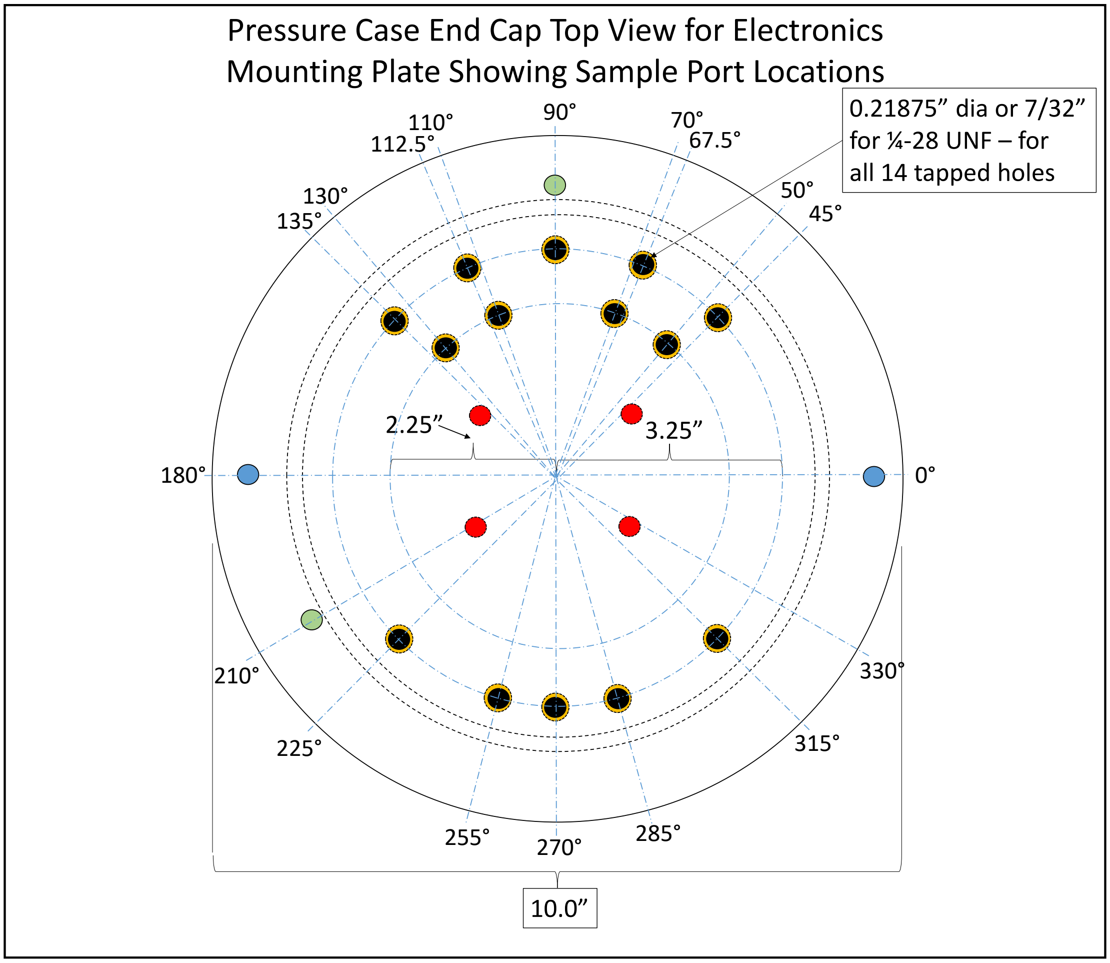

Figure 7: Top view of the electronics mounting plate end cap. The feed-through holes are shown in black. The reference lines and circles are indicated with blue dashed lines.

6. Turn the end cap over so that the piston is facing up. The holes drilled in Step 4 above will mark the location where you will use a 0.328" (21/64") drill bit to drill down **only** 1.125" into the piston at each of the 14 locations as shown in Figure 8 (yellow circles).

7. Use a 1/8-27 tap to thread all 14 holes.

8. This will complete the fabrication on the electronics mounting plate end cap.

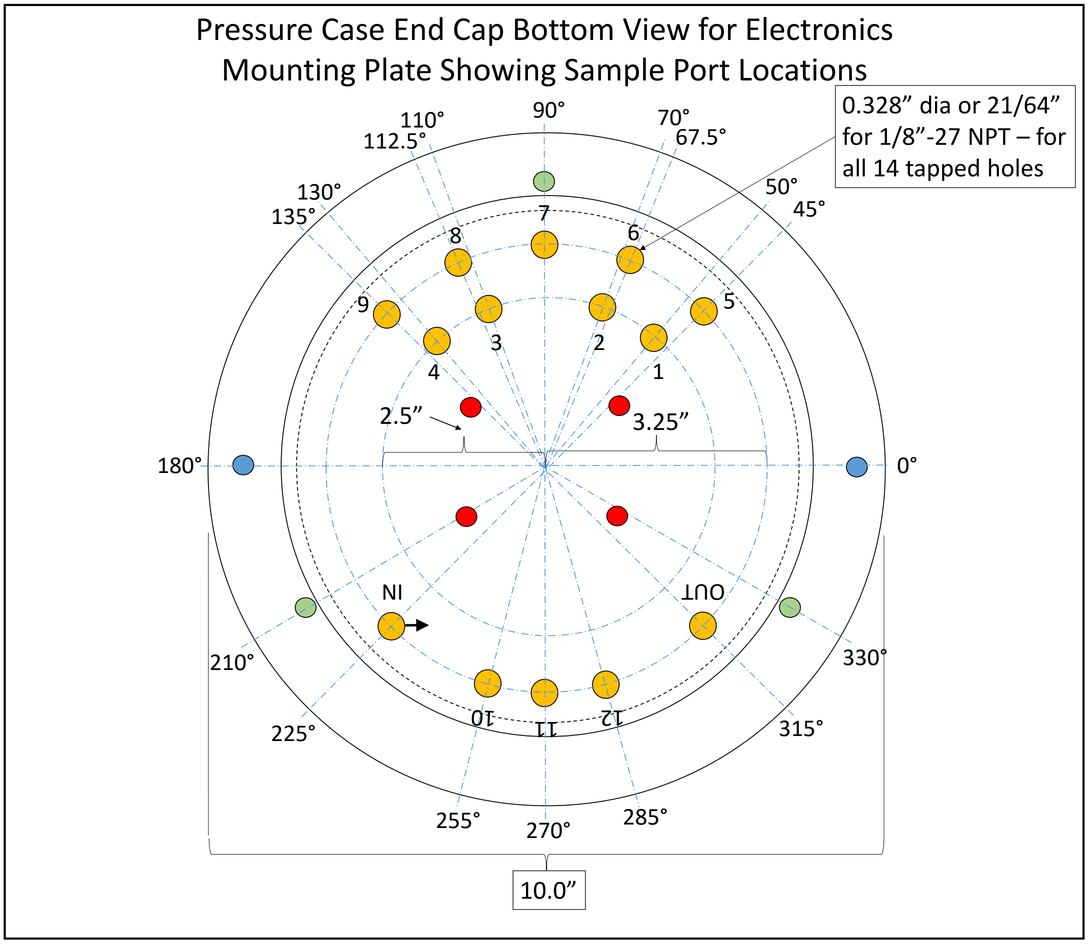

Figure 8: Bottom (piston) view of the electronics mounting plate end cap. The feed-through holes are shown in yellow. The reference lines and circles are indicated with blue dashed lines. . The number indicated the sample port locations and the intake (IN) and out take (OUT) port locations.

9. *Assembly:* To install the 1/8" NPT fitting on the piston face, pipe thread tape or thread sealer is required as these fittings need to be water tight. No sealer is required for the outer ¼-28 fittings.

    a. Apply pipe thread tape or sealer to each of the 1/8" tube to 1/8" NPT fittings (McMaster PN: 5463K438) and install them into all ports except the intake port (IN).

    b. Apply pipe thread tape or sealer to each of the 3/16" tube to 1/8" NPT elbow fittings (McMaster PN: 2974K208) and install it into the intake port (IN) until it points towards the OUT port as indicated by the arrow in Figure 8.

    c. Thread in the four 2-½" aluminum 10-32 standoffs (McMaster PN: 93505A031) into the threaded holes on the piston.

    d. Turn the end cap over and rest it on the aluminum standoffs.

    e. Install the quick turn tube ¼-28 UNF couplings (McMaster PN: 51525K221) into ports 1

to 12 (Figure 9). Then install the protective caps (McMaster PN: 51525K315) to protect the couplings. **Note** that the barbed mates (McMaster PN: 51525K213) to these quick turn connectors are used to attach sample tubes.

f. You can wait to install the IN and OUT barbed tube ¼-28 fittings (McMaster PN: 5047K119) until the all components on the end cap are installed.

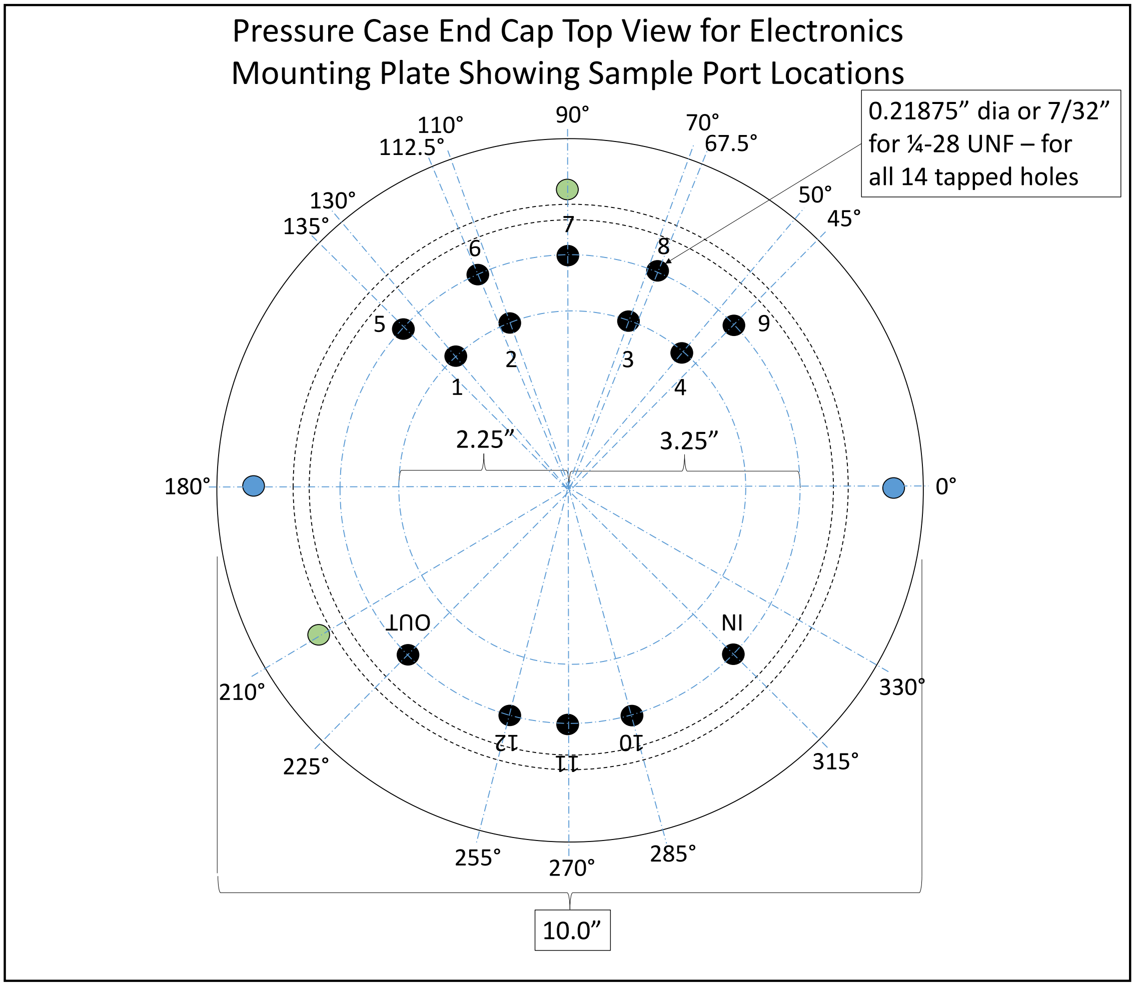

Figure 9: Top view of the electronics mounting plate end cap. The feed-through holes are shown in black. The reference lines and circles are indicated with blue dashed lines. The number indicated the sample port locations and the intake (IN) and out take (OUT) port locations.

C. Valve, Pump, and Manifold Mounting Plate: This acrylic plate will support the 6 port manifold, peristaltic pump and 13 pinch valves and will be mount on top of the 2- 1/2 “ aluminum standoffs on the end cap in figure 8. The electronic mounting plate is made from laser cut 7” OD x 1/8” thick clear acrylic from Delvies Plastics. These discs are laser cut and Delvies Plastics does not provide a part number, but they are easy to order online or these can be order from other vendors. Since there are many holes on this plate, to make it easier to fabricate we will use 3 figures (Figures 10, 11, and 12) to walk through the mockup and fabrication of this acrylic disc. **Note:** First mark on the acrylic disc with TOP, UP, DOWN, LEFT, and RIGHT.

a. Find the center of the acrylic disk and make two perpendicular reference lines across the disk as shown with the red dashed line in Figure 10. These will be your center reference lines for making all subsequent blue reference lines also shown in Figure 10.

b. Measure out, left and right, from the center point along the horizontal center line 1.125” and make a vertical reference line at each mark (Figure 10).

c. Measure up and down from the vertical center line 0.8125” and make a horizontal reference line at each mark. The intersections of these four reference lines will be the location of the 10-32 standoffs holes (Figure 10). These four holes are used to mount the plate on to the four aluminum standoffs.

d. Drill four 0.2031” (13/64”) diameter holes indicated in Figure 10 as yellow circles.

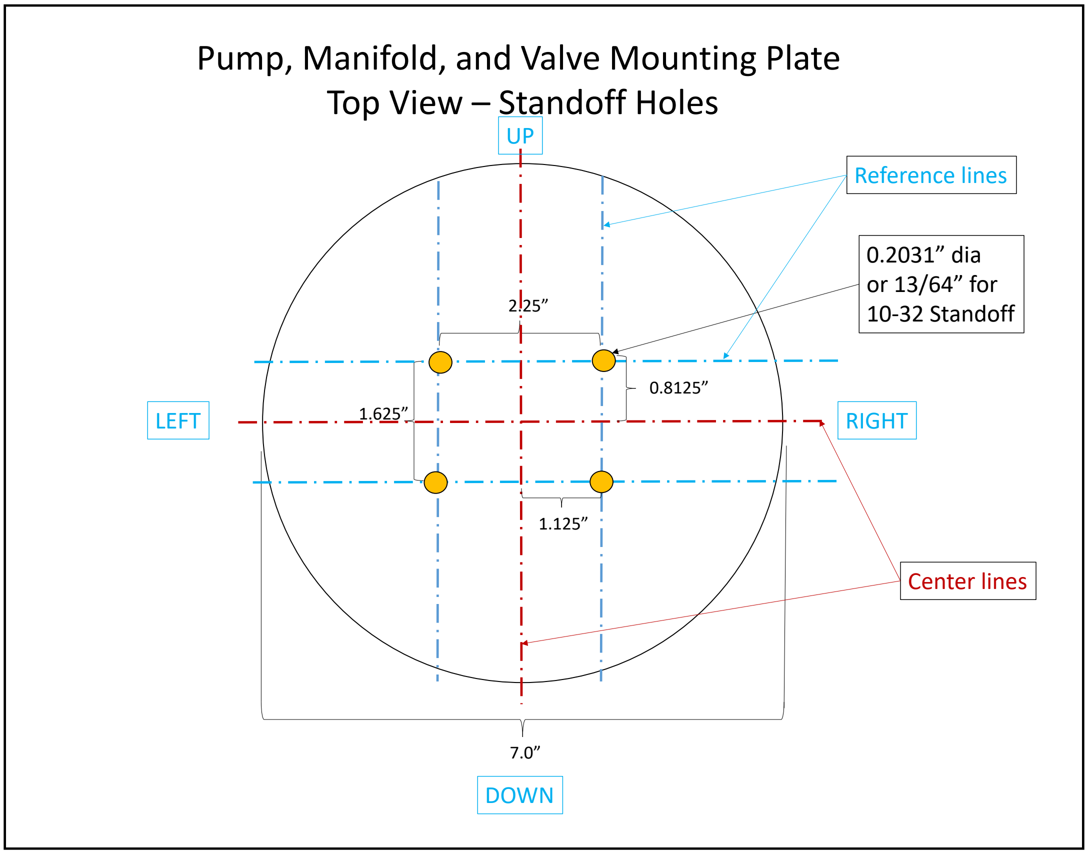

Figure 10: Schematic drawing of the Pump, Manifold, and Valve Mounting Plate illustrating the location of the 10-32 standoff holes (yellow circles). Reference lines are indicated as blue dashed lines and the center lines as red dashed lines.

e. Using the same acrylic disc, measure right and left along the horizontal center line 0.35” and make a vertical reference line at each mark (Figure 11).

f. Measure up and down along the vertical center line 1.5” and make a horizontal reference line at each mark. Where the lines intersect at the upper left and lower right (Figure 11, yellow circles) are the locations of the manifold mounting holes.

g. Drill two 0.125” (1/8”) diameter holes at these locations (Figure 11).

h. Measure right along the horizontal center line 1.25” and make a vertical reference line at this mark (Figure 11).

i. Measure up and down along the vertical center line 0.9375 and make a horizontal reference line at each mark (Figure 11). The intersections of these lines mark the location of the two peristaltic pump mounting holes (Figure 11, black circles).

j. Drill two 0.109375” (3/32”) diameter holes at these locations (Figure 11).

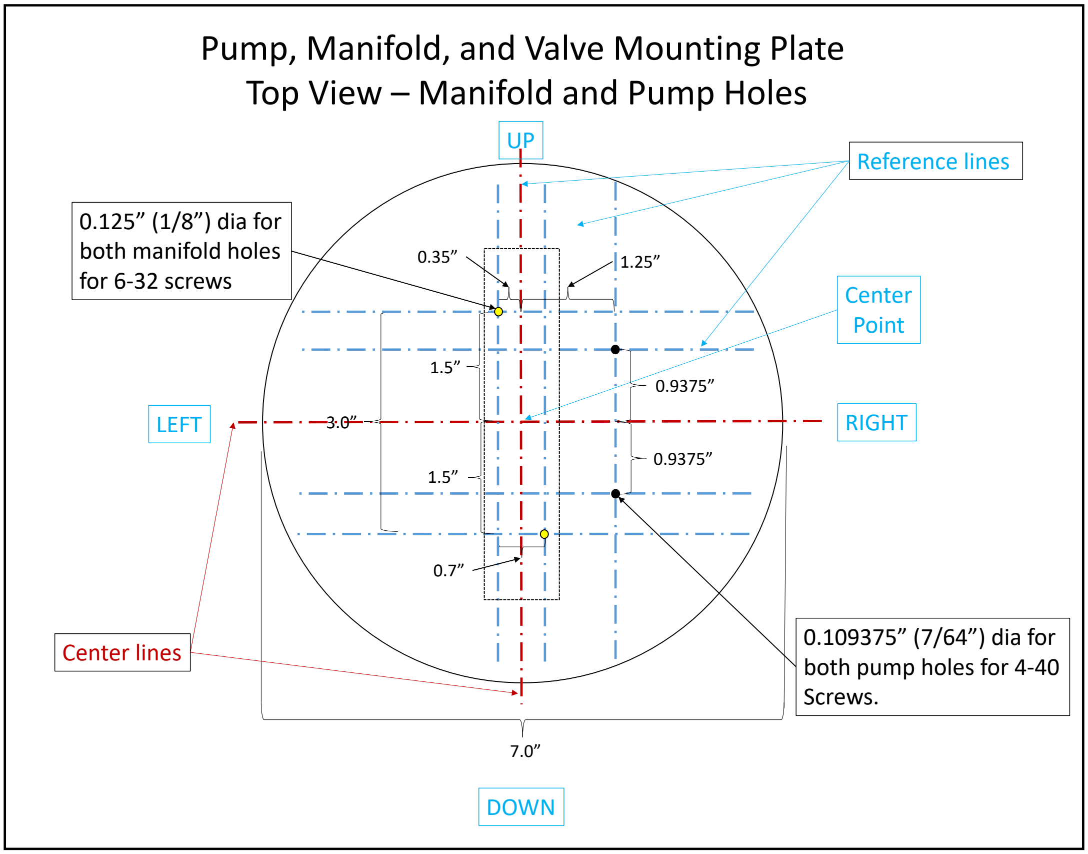

Figure 11: Schematic drawing of the Pump, Manifold, and Valve Mounting Plate illustrating the locations of the 6-32 manifold (yellow circles) and the 4-40 peristaltic pump (black circles) mounting holes. Reference lines are indicated as blue dashed lines and the center lines as red dashed lines.

k. Continuing to use the same acrylic disc, use Figure 12 to apply more reference lines to mark the drill locations for the pinch valves. The factory width between the two holes on the base of each pinch valve is 0.626” which is a specific measurement for proper fitment of the valves to the acrylic disc. The other measurements are important to maintain proper spacing, but if they are off by a small margin it is not as critical.

l. Measure right along the horizontal center line and make a mark at 1.0625”, 2.0”, and 2.378”, then make a vertical reference line at each measurement.

m. Measure left along the horizontal center line and make a mark at 0.25”, 1.625”, 1.75”, 2.0”, and 2.378”, then make a vertical reference line at each measurement.

n. Measure up along the vertical center line and make a mark at 1.0”, 2.0”, and 2.375”, then make a horizontal reference line at each measurement.

o. Measure down along the vertical center line and make a mark at 0.3125”, 1.625”, 2.0”, and 2.375”, then make a horizontal reference line at each measurement.

p. With all of the reference lines complete, the locations of the drill holes can be applied to the acrylic disc. Note that the location of one hole to each pinch valve is at an intersection of the reference lines. The second hole must be precisely 0.626” from the location for proper pinch valve fitment. Mark all 26 locations and drill using 0.109375” (7/64”) diameter holes.

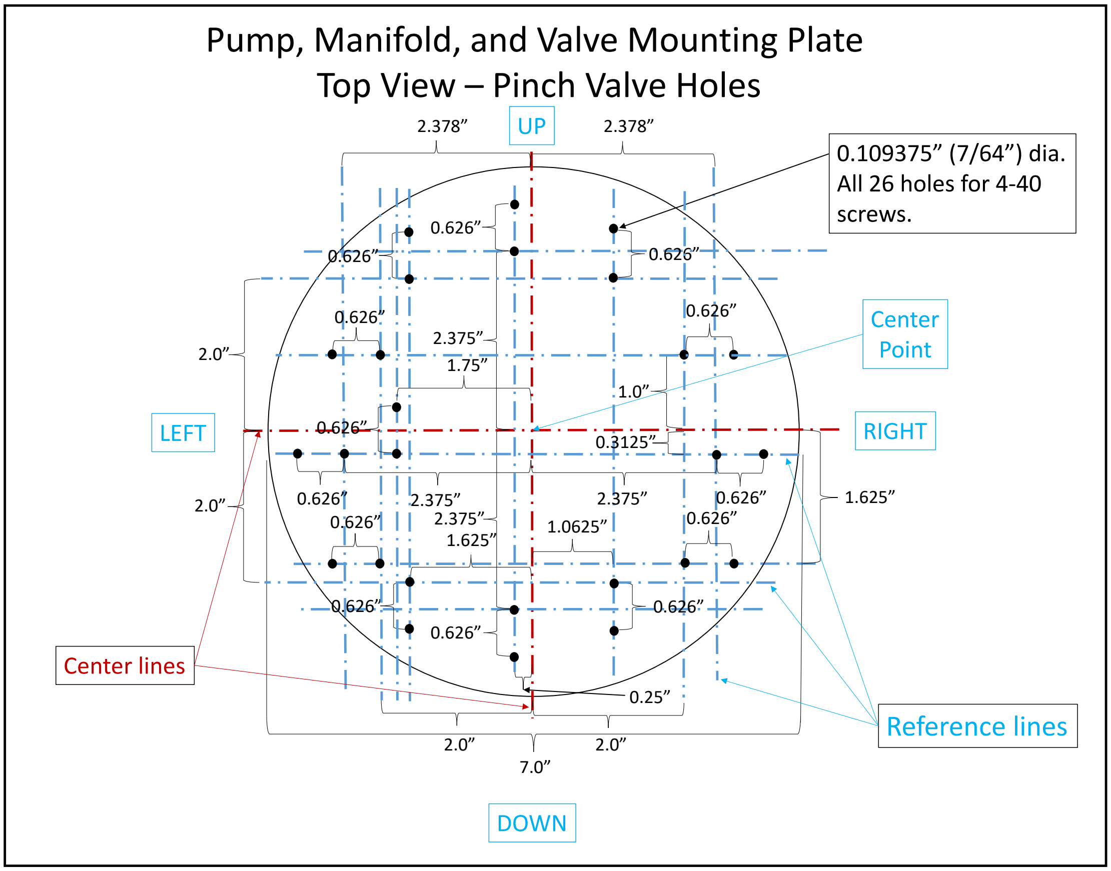

Figure 12: Schematic drawing of the Pump, Manifold, and Valve Mounting Plate illustrating the locations of the 4-40 pinch valves (black circles) mounting holes. Reference lines are indicated as blue dashed lines and the center lines as red dashed lines.

D. Valve, Pump, and Manifold Mounting Plate Assembly: With the fabrication of the plate complete the manifold, peristaltic pump, and pinch valves can be mounted onto the plate.

1. *Clippard ¼” Pinch Valves:* The acrylic disc is designed so that 6 pinch valves are mounted on top and 7 pinch valves and a peristaltic pump are mounted on the bottom of the disc. Note that 12” of tubing comes installed on each pinch valve. Do not remove this tubing as it will

be used for the installation, but it will need to be adjusted as described in Section 1D Step 4b below. Attach the 13 Clippard ¼” pinch valves (Clippard PN: NPV-1C-05-12) to the acrylic plate using two 4-40 x 3/8” long stainless steel screws (McMaster PN: 91772A108). Note that there are only 2 holes on the plastic portion of the pinch valve body and that they are offset as illustrated on Figure 13. The circles with the solid line are mounted on the top of the acrylic disc and the circles with the dashed line are mounted on the bottom.

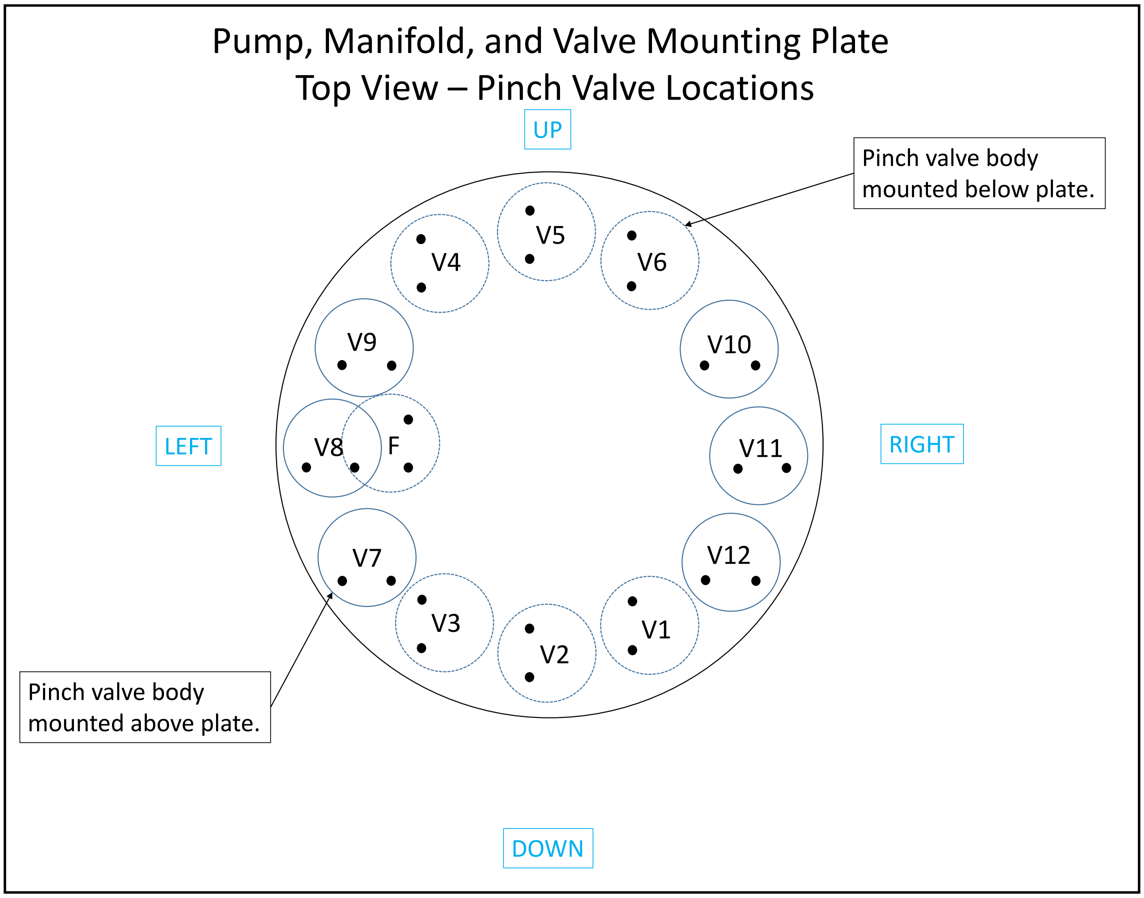

Figure 13: Clippard ¼” pinch valve locations on the acrylic disc. Circles with solid lines are mounted on the top face of the disc and the circles with dashed lines are mounted on the bottom face of the disc.

2. *Manifold:* Before the manifold can be mounted to the acrylic disc the nylon fitting must be attached using thread tape or sealer.

a. Apply thread tape or sealer to two ¼” NPT to 1/8” tube elbow fittings (McMaster PN: 5463K133) and attach them to the end ports on the 6-port manifold (McMaster PN: 5364K231). Tighten the fittings such that the openings of the tube ends are pointing in opposite direction angled slightly downward (~10°) from horizontal as indicated by the black arrows in Figure 14. **Caution:** Do not over tighten or the manifold body could crack.

b. Apply thread tape or sealer to the six 1/8” NPT to 1/8” tube tee fittings (McMaster PN: 5463K55) and attach them to the 6 ports on the top of the manifold. Tighten them until

the tee is perpendicular to the body of the manifold as indicated by the double arrows of Figure 14. **Caution:** Do not over tighten or the manifold body could crack.

c. Align the mounting holes with the holes on the acrylic disc. Note that the screws securing V5 and V2 interfere with the manifold from sitting flat on the disc. Use an abrasive tool to remove a portion of the manifold body material so that the manifold will sit flush with the face of the disc.

d. Attach the manifold to the acrylic plate using two 6-32 x 1.25" long stainless steel screws and matching nuts (McMaster PN: 91772A155 and 91841A007).

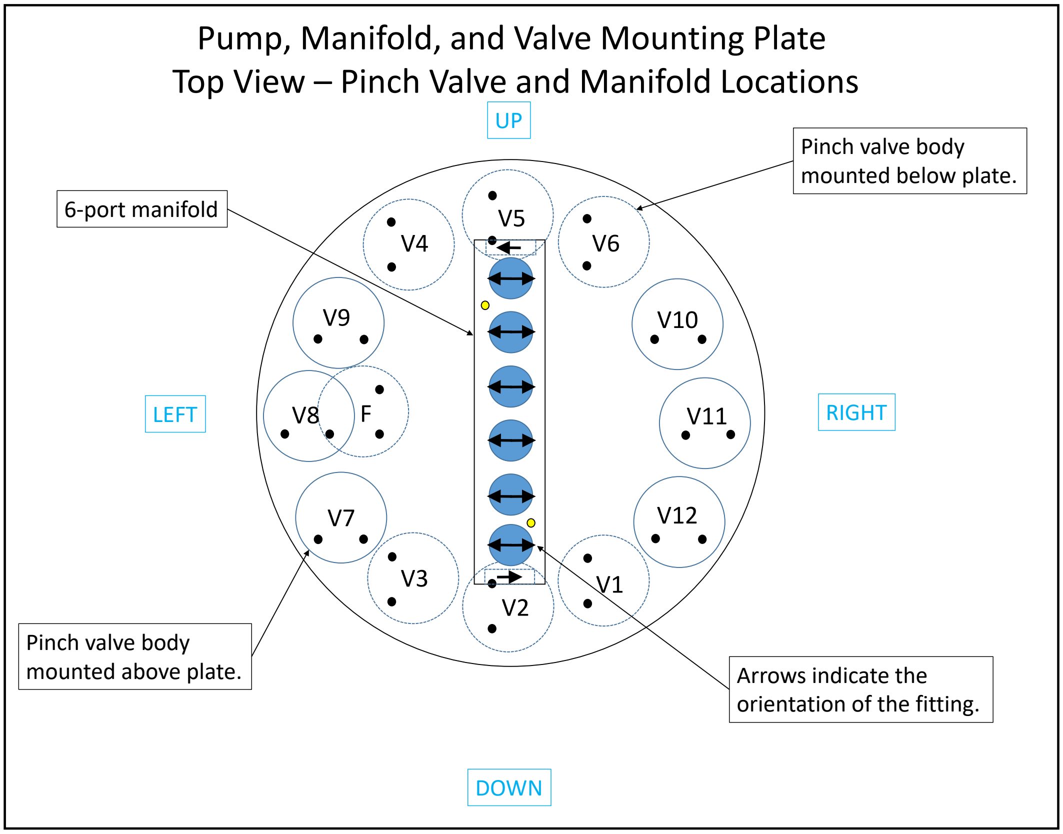

Figure 14: Location of the 6-port manifold on the acrylic disk. The single arrow denotes the direction of the ¼" NPT elbow fittings and the double arrows indicate the orientation of the 1/8" NPT tee fittings.

3. *Thomas Peristaltic Pump:* The Thomas peristaltic pump (Thomas PN: AV 2050-0503) does not come wired. On the motor there are two terminals are marked with a + (positive) and – (negative).

a. To wire the pump, cut an 18" piece of 22 AWG red and black wire (Jameco PN: 2153705) and trim off a small portion of insulation from one end of each wire. Solder the trim end of the red wire to the positive (+) terminal and the trimmed end of the black wire to the negative (-) terminal on the peristaltic pump. Slide heat shrink tubing (Jameco PN: TT74901) onto each of the two wires to cover the solder and terminal. Note: These two wires can be twisted together to make a bundle and make for a cleaner installation.

b. The head orientation on the peristaltic pump will need to be changed. There are two tabs on the side of the head. Squeeze them inward, pull the head away from the base until the tabs clear the base, then rotate clockwise 90°, and then push the head towards the base to secure the new orientation as indicated by the arrow in Figure 15.

c. Attach the peristaltic pump to the acrylic plate using two 4-40 x 5/8” long stainless steel screws and matching nuts (McMaster PN: 91772A112 and 90257A005) in the orientation shown in beige on Figure 15.

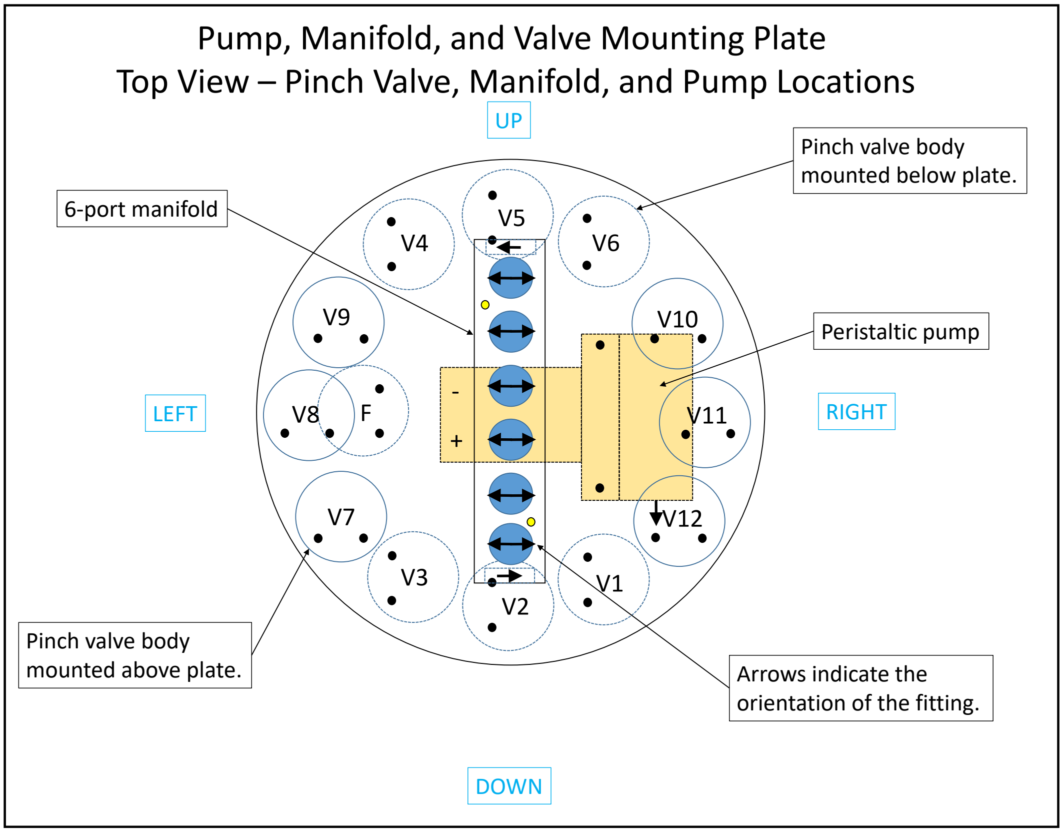

Figure 15: Location of the peristaltic pump on the acrylic disk (beige). The single arrow denotes the orientation of the pump head.

4. *Attach Pump, Manifold, and Valve Mounting Plate*: With the assembly of the acrylic disc completed it can now be attached to the pressure case housing end cap.

a. Place the competed mounting plate on top of the aluminum standoffs on the end cap as shown in Figure 16. Note that the peristaltic pump is located below pinch valves 10, 11, and 12. Do not secure the acrylic disc to the standoffs.

b. At this time it will be necessary to adjust the tubing installed in each of the pinch valves. To slide the tubing in the valve, the piston pressure needs to be released. This can be performed by physical means, but you risk damaging the tubing. The preferred method is to use a 12VDC power source to activate each of the valves as you adjust the position in the valve. The tubing in the pinch valves installed on the top of the acrylic plate will connect directly to the tee fittings on the manifold as numbered (7 to 12) in Figure 16.

The tubing in the pinch valves on the bottom of the acrylic plate need to be routed around the edge of the acrylic plate and attached to the tee fittings on the manifold as numbered (1 to 6) in Figure 16. The flush valve (F) will connect to the elbow fitting on the end of the manifold labeled F.

c. Activate the valves one by one and slide the tubing enough to attach one end of the tube to its respective numbered tee fittings on the manifold as shown in Figure 16 and secure with a cable tie (Jameco PN: 126544) and trim off the excess.

d. Attach the other end of the tubing in each pinch valve to its respective port on the end cap. A bit of creativity is required to maneuver the tubes so that they do not kink or pinch. **Do not** cut the tubing as all tubes must have the same length. Secure all connections with cable ties (Jameco PN: 126544) and trim off the excess cable tie tail.

e. It will be necessary to cut the tubing installed in the peristaltic pump. Align the tube coming out of the bottom of the pump with the elbow fitting on the pressure case end cap. Mark to length, cut, attach, and secure with a cable tie (Jameco PN: 126544) and trim off the excess cable tie tail. The tube coming from the top of the pump will not be long enough. Insert the 3/16” to 1/8” tube adapter (McMaster: 5463K626) into the 3/16” ID tubing on the pump and secure with a cable tie (Jameco PN: 126544) and trim off the excess.

f. Cut a 1.75” length of silicone 1/8” ID x 1/4” OD tubing (Clippard PN: SIH1-0804-NAS-005) and attach to the other end of the 3/16” to 1/8” adapter. The free end of this silicone tubing will be routed around the edge of the acrylic disc and attached to the elbow connector on the end of the manifold labeled P in Figure 16. Secure both connections with a cable tie (Jameco PN: 126544) and trim off the excess.

g. Recheck all tubing connections going to the 14 ports on the end cap and the connections on the 6-port manifold, place the assembled pump, valve, and valve mounting plate on to the 4 aluminum standoffs and secure using four 10-32 x ½” long stainless steel screws (McMaster PN: 91772A829) to attach the acrylic plate to the standoffs as shown in Figure 16.

h. Figure 17 show a schematic of the AutoSampler plumbing. The blue circles with PV represent the pinch valves and the blue square with PP denotes the peristaltic pump. The black and blue arrows indicated the flow direction. The blue line color shows the primary flow path and connection between the In and Out ports controlled by the flushing valve.

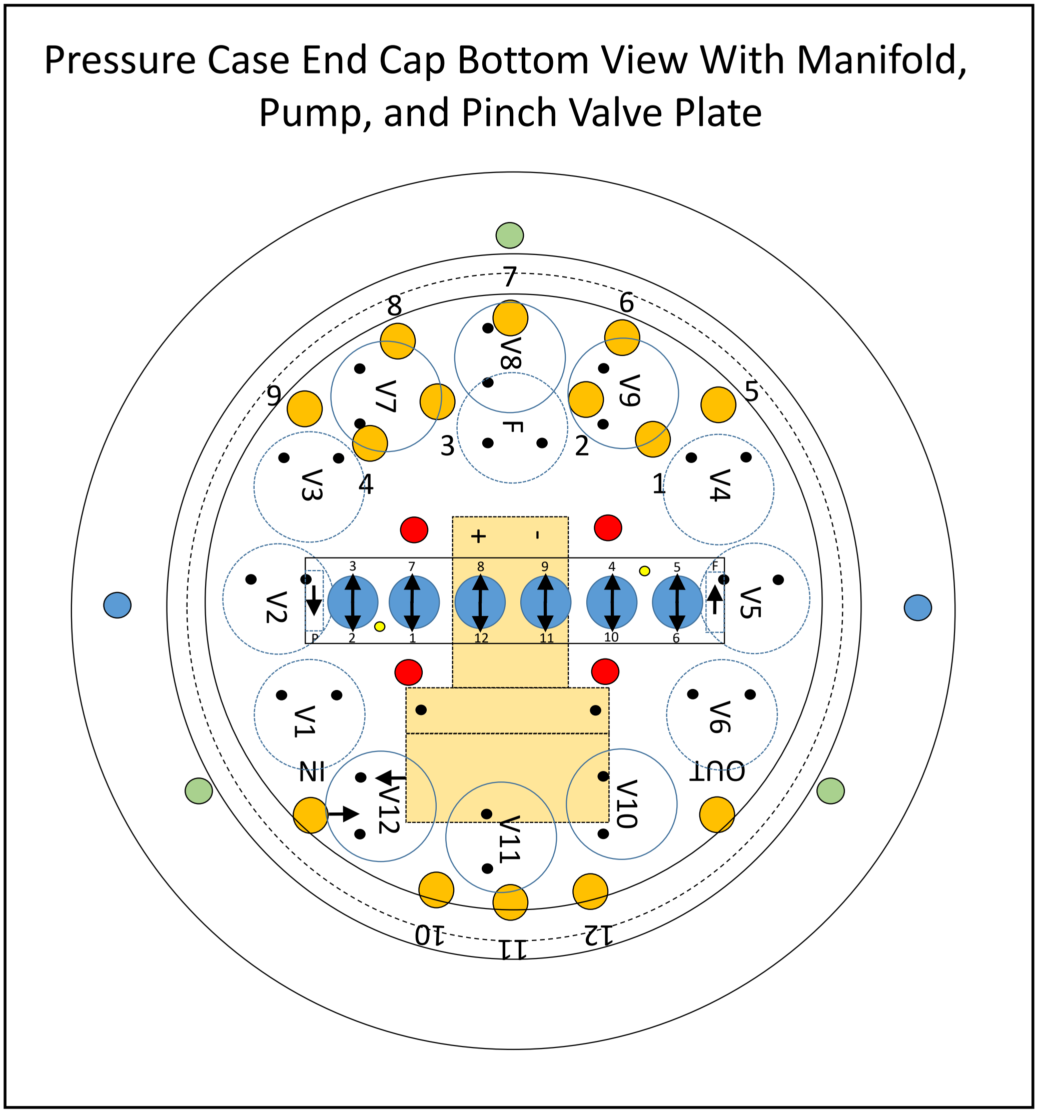

Figure 16: Schematic of the Manifold, Pump, and Pinch Valve Plate mounted on the pressure case end cap.

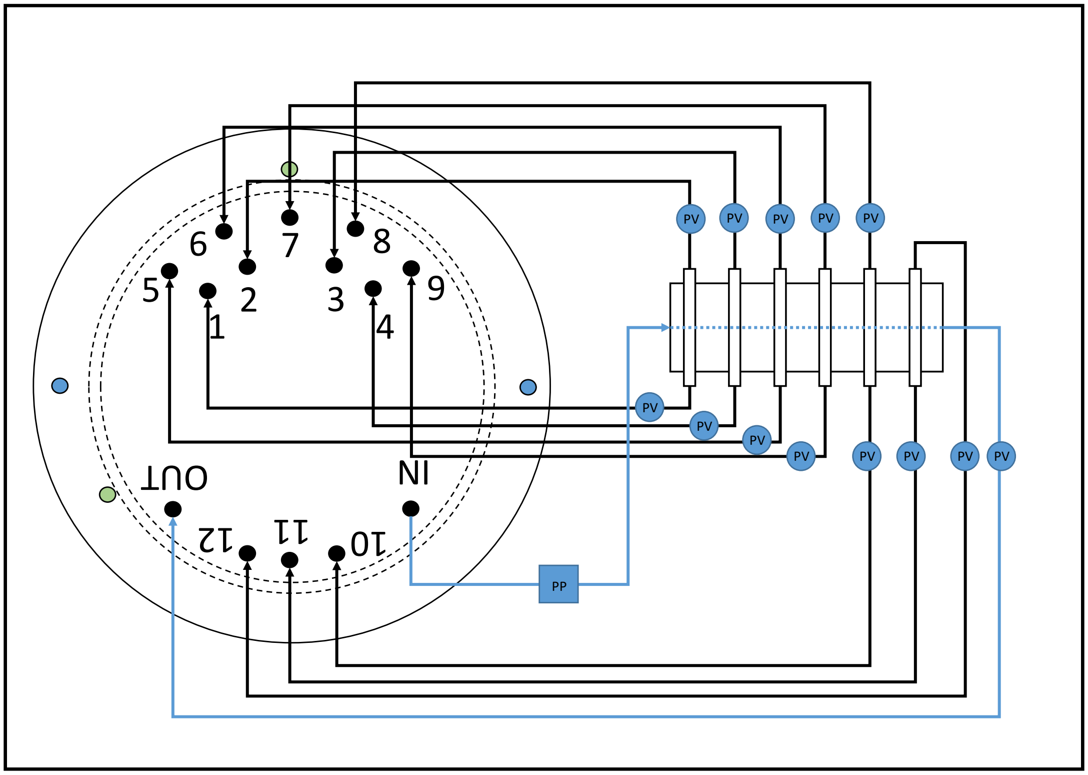

Figure 17: Schematic of the Manifold, Peristaltic pump (PP), and Pinch Valves (PV), direction of flow and relation to the fittings on the pressure case end cap.

E. **Electronics Mounting Plate:** The electronics mounting acrylic plate will support the two 8 channel relay boards (RB), an Arduino Pro Mini (AMP) controller, and electrical terminal block. The electronics mounting plate is attached on top of the Manifold, Pump, and Pinch Valve Plate using mounting holes that are on the top of the pinch valves (V7, V9, V10, and V12). The electronic mounting plate is made from laser cut 7" OD x 1/8" thick clear acrylic from Delvies Plastics. Mark on the acrylic disc TOP, UP, DOWN, LEFT, and RIGHT.

1. Find the center of the acrylic disk and make two perpendicular reference lines across the disk as shown with the red dashed line in Figure 18. These will be your center reference lines for making all subsequent blue reference lines also shown in Figure 18.

2. Measure out, left and right, from the center point along the horizontal center line 0.125, 2.0625" (0.125" + 1.9375"), and 2.626" and make a vertical reference line at each mark (Figure 18).

3. Measure up from the vertical center line 1.0" and 2.59375" and make a horizontal reference line at each mark.

4. Measure down from the vertical center line 1.625" and 2.59375" and make a horizontal reference line at each mark.

5. The intersections of these reference lines will mark the various drill locations as shown on Figure 18. Drill eight 0.09375" (3/32") diameter holes for 2-56 standoffs that will be used to secure the two relay boards to the acrylic disc (Figure 18, green circles).

6. Drill five 0.109375” (7/64”) diameter holes for 4-40 stainless steel screws to secure the acrylic disc to the pinch valves and for the electrical terminal block (Figure 18, black circles).

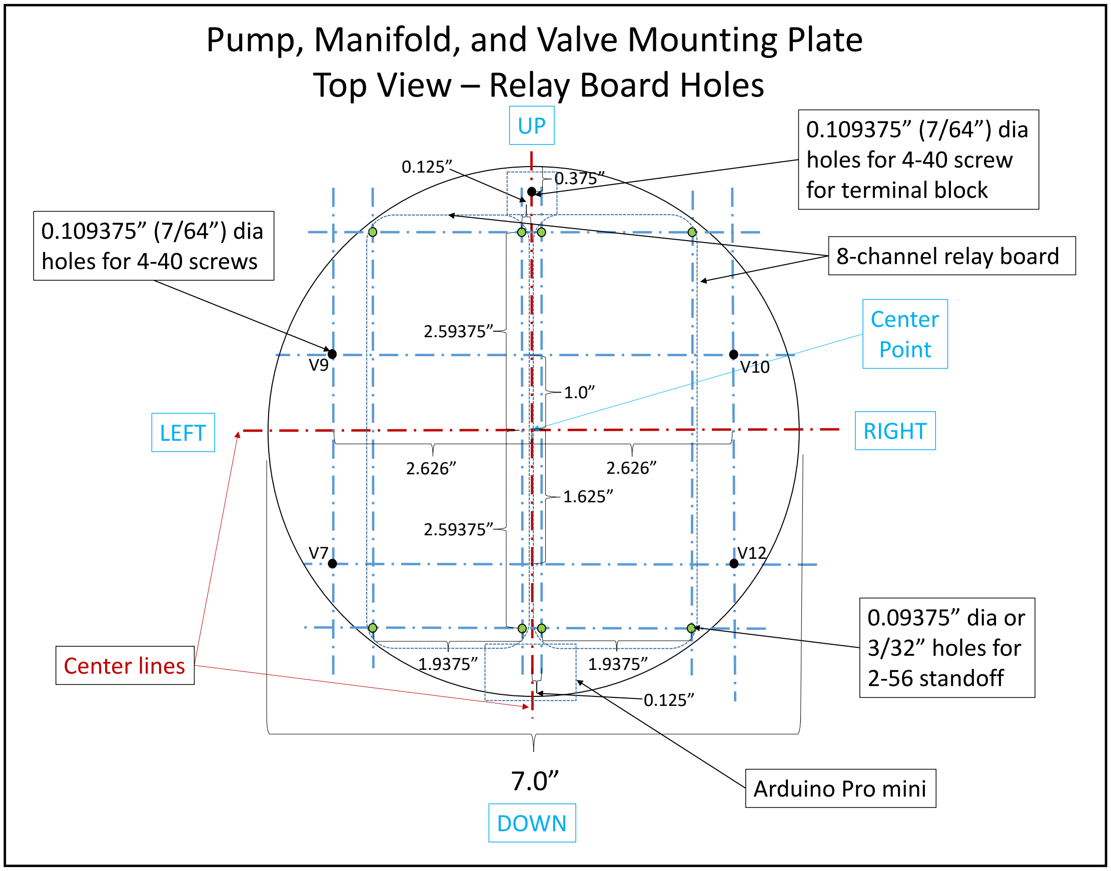

Figure 18: Schematic of the Electronics Mounting Plate illustrating the locations of the 4-40 pinch valves and terminal block (black circles) mounting holes and 2-56 standoff mounting holes (green circles). Reference lines are indicated as blue dashed lines and the center lines as red dashed lines.

F. Electronics Mounting Plate Assembly: With the fabrication of the acrylic disc complete, assembly can begin.

1. Attach eight nylon 2-56 x ¼” long standoffs (McMaster PN: 92745A370) to the acrylic disc using 2-56 x ¼” long stainless steel screws (McMaster PN: 94735A707) at the location of the green circles on Figure 18.

2. Place the two 8-channel relay boards (Sparkfun PN: 101-70-102) on to the ¼” standoffs and secure using six 2-56 x 0.75” long nylon standoffs (McMaster PN: 92319A275). Note: The ¼” standoffs over pinch valve V2 (next to the location of the Arduino Pro Mini) will be secured using two 2-56 nylon nuts (McMaster PN: 94812A100).

3. Attach a 2-port electrical terminal block (Jameco PN: 215011) to the acrylic disc at the location indicated on Figure 19 above pinch valve V5. **Note:** The terminal block comes with 8 ports. Use a utility knife to slice off 2 ports.

4. Orient the electronics mounting plate so that the terminal block is located above pinch valve V5 (Figure 19). Attach the acrylic disc to the top of the pinch valves using 4-40 x 3/8" long stainless steel screws (McMaster PN: 91772A108) at V7, V9, V10, and V12 (Figure 19).

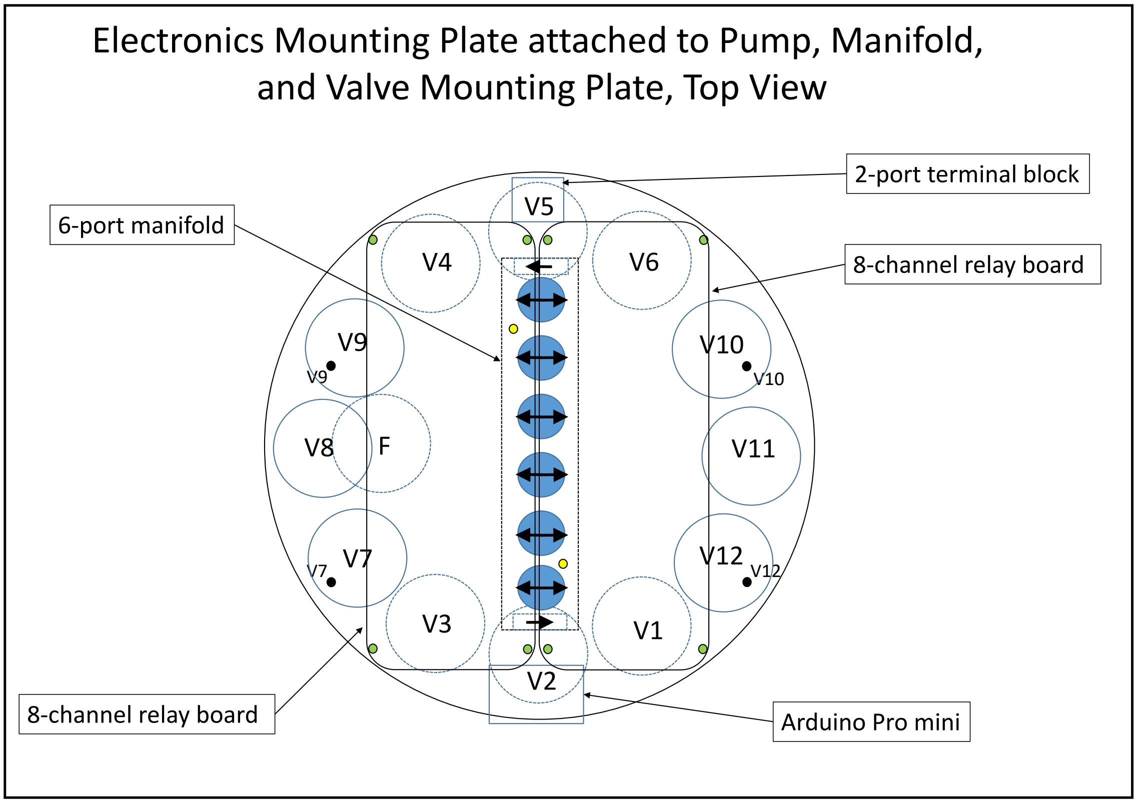

Figure 19: Schematic of the Electronics mounting plate mounted on top of the Manifold, Pump, and Pinch Valve Plate mounting plate.

G. **Electronics Mounting Plate Cover:** The electronics mounting plate cover protects the electronics and support the real-time clock (RTC) and attaches to the standoffs on the Electronics Mounting Plate.

1. The electronic mounting plate is made from laser cut 7" OD x 1/8" thick clear acrylic from Delvies Plastics. Mark on the acrylic disc TOP, UP, DOWN, LEFT, and RIGHT. The acrylic disc is designed so that it will mount on top of the 2-56 nylon standoff securing the two 8-channel relay boards.

2. Find the center of the acrylic disk and make two perpendicular reference lines across the disk as shown with the red dashed line in Figure 20. These will be your center reference lines for making all subsequent blue reference lines also shown in Figure 20.

3. Measure out left from the center point along the horizontal center line 0.125, 2.0625" (0.125" + 1.9375"), 2.4375", and 3.125" (2.4375 + 0.6875") and make a vertical reference line at each mark (Figure 20).

4. Measure out right from the center point along the horizontal center line 0.125, 2.0625" (0.125" + 1.9375"), and 2.4375" and make a vertical reference line at each mark (Figure 20).

5. The intersections of these reference lines will mark the various drill locations as shown on Figure 20. Drill eleven 0.09375” (3/32”) diameter holes for 2-56 standoffs and stainless steel screws (Figure 20, green circles).

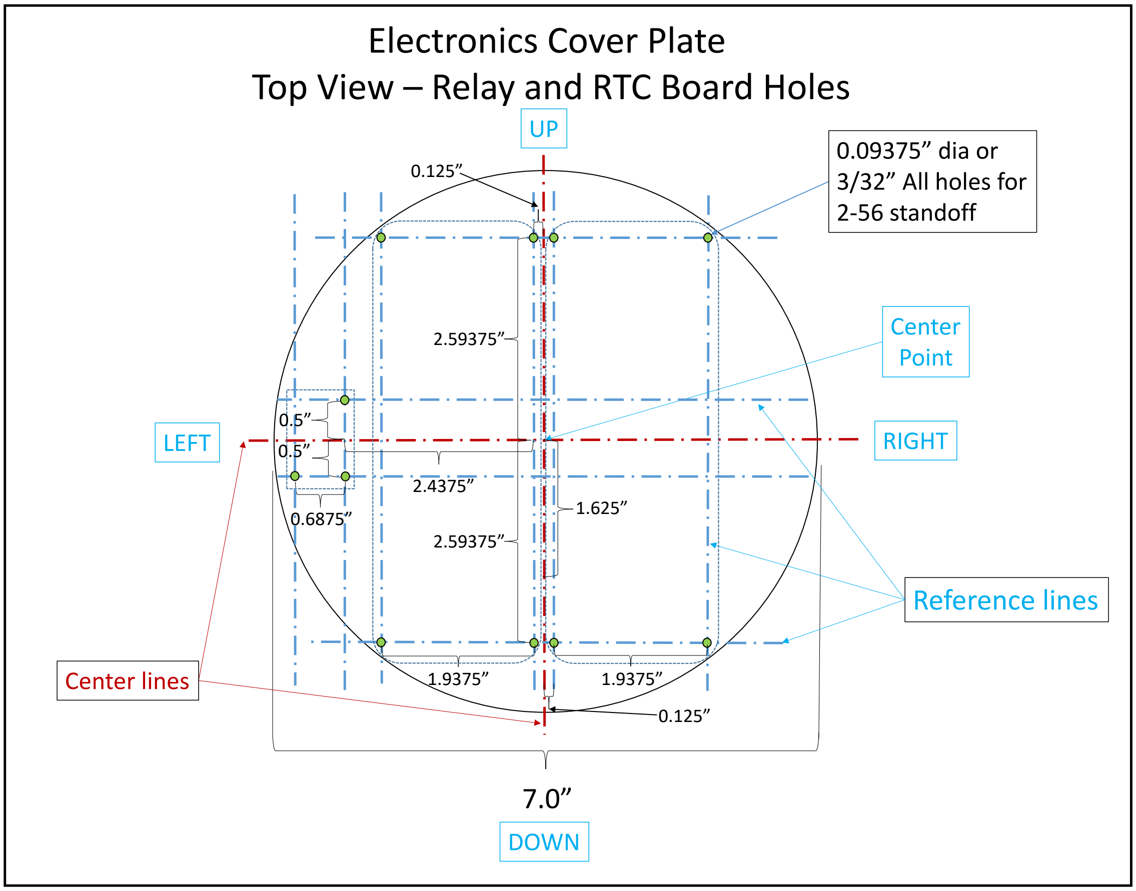

Figure 20: Schematic of the Electronics Cover Plate illustrating the locations of the 2-56 standoff mounting holes (green circles). Reference lines are indicated as blue dashed lines and the center lines as red dashed lines.

H. Electronics Cover Plate Assembly: With the fabrication of the acrylic disc complete, assembly can begin.

1. Attach 3 nylon 2-56 x ¼” long standoffs (McMaster PN: 92745A370) to the acrylic disc using 2-56 x ¼” long stainless steel screws (McMaster PN: 94735A707) at the location of the green circles on Figure 21.

2. Mount the RTC (Amazon PN: DS3231) onto the 2-56 standoffs such that the lithium battery holder is pointing away from the acrylic disc and secure using three 2-56 nylon nuts (McMaster PN: 94812A100). Install the CR2032 lithium battery.

3. The Electronics cover plate cannot be connected until the wiring is completed. When completed, this acrylic plate will secure on top of the six 2-56 x ¾” long standoffs using six 2-56 x 5/16” long stainless steel screws (McMaster PN: 91772A078).

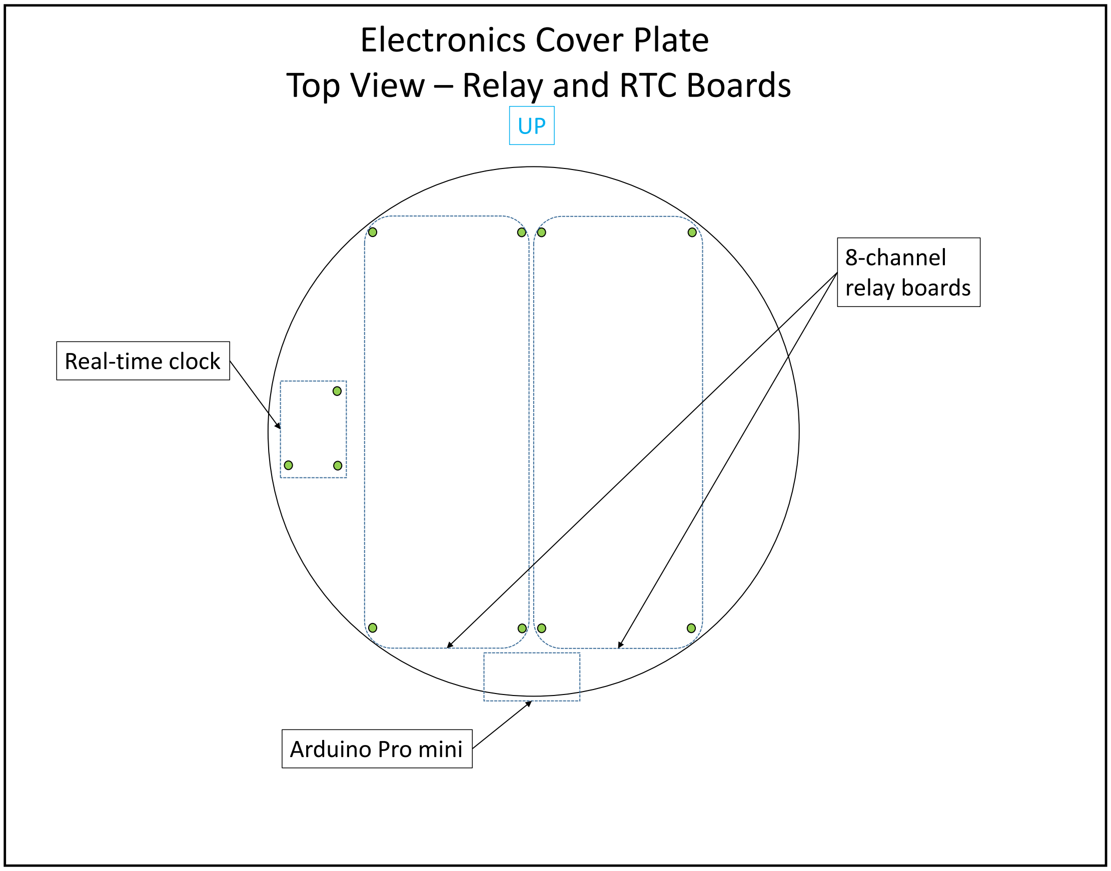

Figure 21: Schematic of the Electronics cover plate mounted on top of the Electronics Mounting Plate. The dashed rectangles indicate the location of the electronics boards.

I. **Arduino Pro Mini Assembly and Installation:** The Arduino Pro Mini (APM) does not come with the header pins installed. To accomplish this, some soldering is required on the APM. When you order the APM, some distributors will include straight header pins. While this can be used we prefer to use 90° header pins and to custom bend the header pins to approximately 45° because it makes a lower profile and it is easier/cleaner to run the wires to the various components on the plate if the jumpers run horizontally. Fortunately the RTC and the two 8-channel relay board require no soldering and are ready to use. Follow the steps below to assemble the APM board (Sparkfun PN: DEV11113).

1. Break off a block of seven straight header pins (Sparkfun PN: PRT-00553 comes in a block of 40 pins). Insert this block into the APM board labeled GND, VCC, RXI, TXD, facing away from the board, and solder in place. These pins will be used to connect the USB communications cable (Sparkfun PN: DEV-09718).

2. Break off a block of nine straight header pins (Sparkfun PN: PRT-00116). Using a pair of long-nose pliers, bend the short portion of the header pins to approximately 45° and insert into the holes on the APM board labeled GND, 2, 3, 4 to 9 facing inward and solder into place. GND is for the 5VDC ground, 4 through 9 will be used to signal the 8-channel relay to control the pinch valves. Pins 2 and 3 are not used, but are good to have as backup pins.

3. Break off a block of 12 straight header pins (Sparkfun PN: PRT-00116). Using a pair of long-nose pliers, bend the short portion of the header pins to approximately 45° and insert into the holes on the APM board labeled RAW to 10 facing outward and solder into place. RAW will be used for the 12VDC input, GND for the ground, RST is not used, VCC is the 5VDC output to the RTC and for both 8-Channel relay boards, and pins A3 to 10 are used to control more pinch valves and the peristaltic pump.

4. Break off a block of two straight header pins (Sparkfun PN: PRT-00116). Using a pair of long-nose pliers, bend the short portion of the header pins to approximately 45° and insert into the holes on the APM board labeled SDA and SCL (sometimes labeled A4 and A5 on clone boards) and facing outwards towards pins RAW to 10 and solder into place. These two outputs will be used to communicate with the RTC.

5. To attach the APM to the acrylic disc, cut a piece of ¼" thick x ½" wide x 1.25" long double stick foam tape (McMaster PN: 7626A272), apply one side to the back of the APM board and press fit to the acrylic disc between the two 8-channel relay board opposite the side with the 2-port electrical terminal block.

J. **Wiring the Autonomous Muliport Water Sampler:** With most of the electronics components installed on the 3 acrylic discs, wiring of this system can commence. To accomplish this, solder, heat shrink tubing (Jameco PN: TT74901), 22 AWG wire (various colors, Jameco PN: 2153705), soldering iron, a heat gun, cable ties (Jameco PN: 126544), jumpers (Amazon PN: 2260746), 1N4004 rectifier diodes (Digikey PN: 2368-1N4004-ND), and the knowledge of basic soldering will be required. There is a lot of wire to manage on this system and it is important to keep the wires as short and organized as possible for the wiring to fit inside the pressure housing. It is best to begin from the pinch valves and peristaltic pump and work your way to the relays, then to APM controller, and finally to the battery supply. Figure 22 shows the completed wiring and Figure 23 shows all sides of the completed assembly of the autonomous multiport water sampler.

1. *Pinch valves:* The pinch valves come with a long lead of yellow wire attached, all of this wire will not be needed, and with careful management, more wire will not be required. Rectifier diodes are installed for the pinch valves and peristaltic pump to prevent voltage back feed. Without the diodes the APM will reset with the activation of each pinch valve.

   a. Each relay channel has a blue three port terminal where the center and right ports are connected (normally closed) and the center and left ports are not connected (normally open), this build will only be using the normally open center and left ports on the relay board. The center port on the relay board will be designated the common port. Cut fourteen 1.5" and one 6.0" long pieces of 22 AWG yellow wire and trim off a small portion of insulation from each end. Connect the center ports on relays K1 to K8 on both 8-channel relay boards using the 1.5" wire and secure all except for K1 and K8 on Relay Board 1 (RB1) and K1 on Relay Board 2 (RB2). Insert on end of the 6" wire into the center port for K8 on RB1 and into the center port for K1 on RB2, and secure both (Figure 22). These wires will provide 12VDC power to each relay from the battery pack.

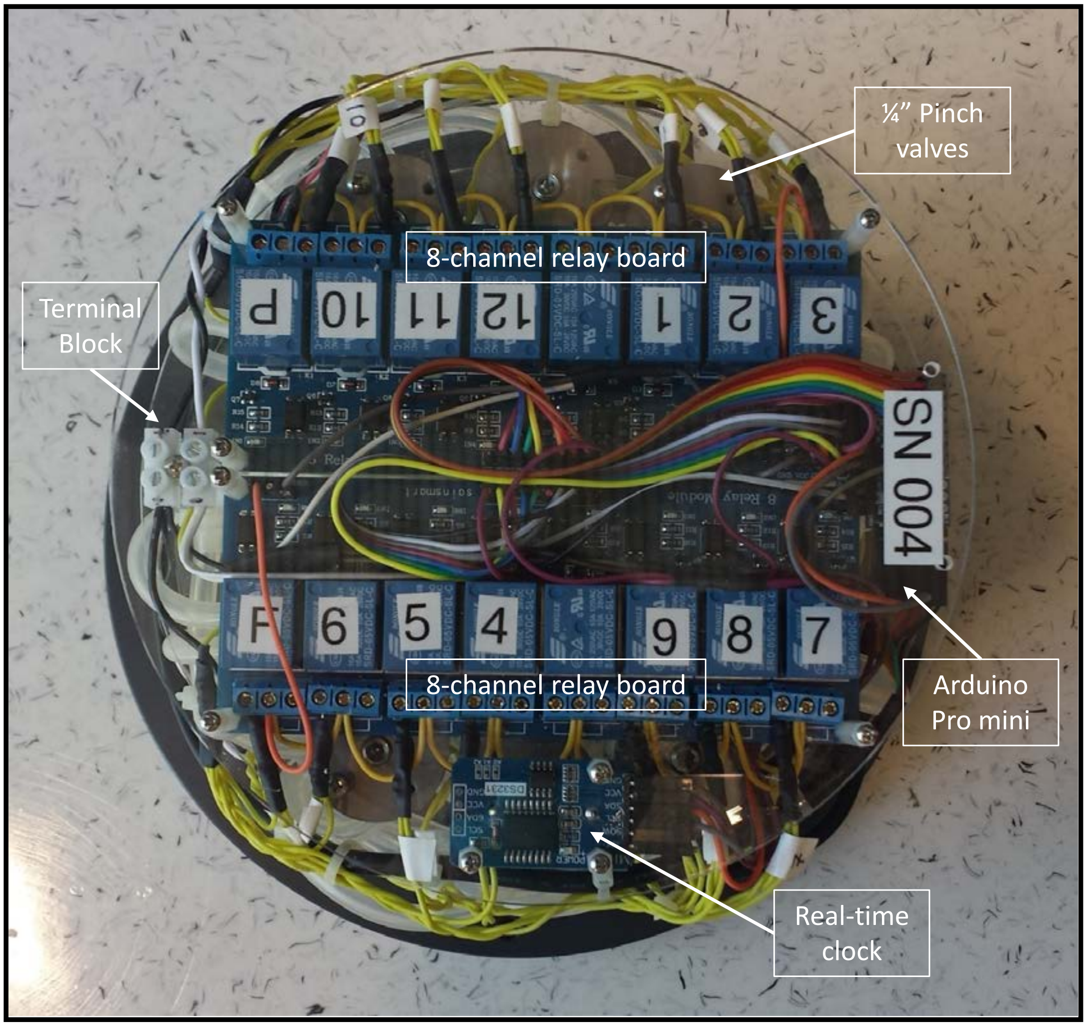

Figure 22: Completed wiring for the autonomous multiport water sampler.

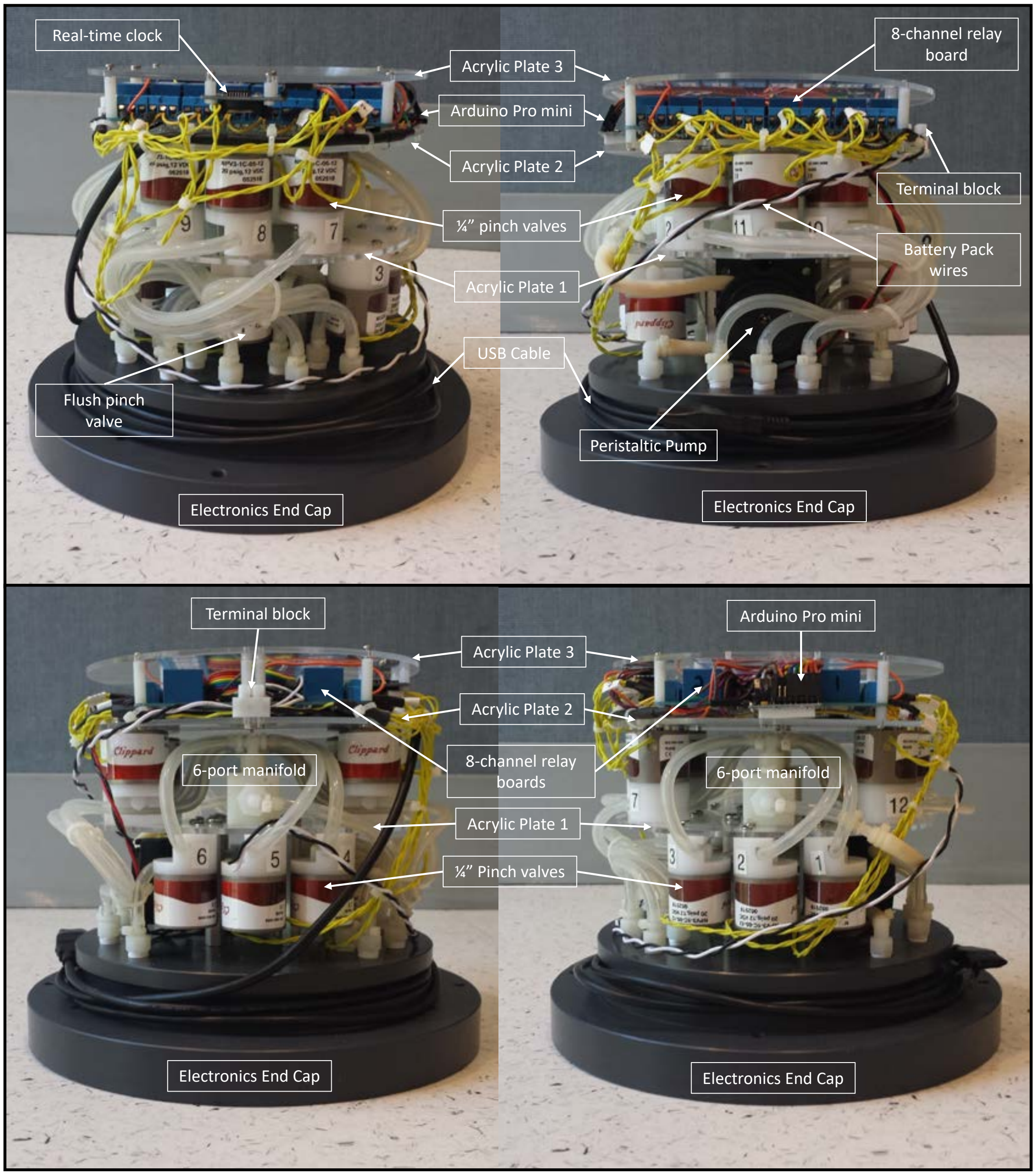

Figure 23: Final assembly of the autonomous multiport water sampler.

b. Begin organizing the wires for each pinch valve (PV) and the peristaltic pump by twisting the 2 wires on the individual pumps together to make a "braided" look so that the two wires are a single strand long enough to reach its assigned relay according to Table 1.

Table 1: Pinch valve, peristaltic pump, RB1, and RB2 APM GPIO address information.

Pinch Valve ID or Pump (P)	Relay Board #	Relay #	Jumper color	Relay input	APM GPIO ID
P	1	K1	Purple	IN1	A2
10	1	K2	Blue	IN2	4
11	1	K3	Green	IN3	5
12	1	K4	Yellow	IN4	6
		K5		IN5	
1	1	K6	Orange	IN6	7
2	1	K7	Red	IN7	8
3	1	K8	Brown	IN8	9
7	2	K1	Black	IN1	A3
8	2	K2	White	IN2	13
9	2	K3	Gray	IN3	A0
		K4		IN4	
4	2	K5	Purple	IN5	A1
5	2	K6	Blue	IN6	10
6	2	K7	Green	IN7	11
F	2	K8	Yellow	IN8	12

c. Begin with PV3 to K8 on RB1 and work towards the terminal block.

1. Bring the strand of 2 wires from PV3 up to K8 on RB1 with a slight catenary arc, cut both wires (save the excess wire), and trim a portion of insulation from both wires coming from PV3.

2. Trim a small portion of insulation one of the wires coming from PV3 and solder it to the gray end of the rectifier diode, close to the diode body because the wire from this end of the diode will eventually insert into the left port on K8.

3. Take one of the excess pieces of wire just cut from PV3, trim off a small portion of insulation from one end, then combine it with the second wire from PV3, and solder it to the black side of the rectifier diode, close to the diode body (Figure 24).

4. Trim off the excess wire from the black end of the diode.

5. Bring the wire soldered to the gray end along the body of the diode.

6. Cut enough length of heat shrink tubing long enough to cover the diode and the soldered joints and apply heat to shrink the tubing to the diode.

7. Trim enough lead on the gray side of the diode so that it will have enough wire to insert into the left port on K8 and then secure in place.

8. Repeat for the remaining pinch valves and for the peristaltic pump.

9. The excess wires coming from the black side of each diode will come together to make the negative return from the pinch valves and pump. Make 2 bundles by

braiding the excess wire from PV1, PV2, and PV3 together, and then braid the excess wire from PV10, PV11, and PV12 together, since the pump (P) is closest to the electrical terminal block it doesn't need to be grouped. Use some cable ties to tidy up and secure the two braids and bring the group of wires approximately equal to the arc of the acrylic disc and about 2.5" from the terminal block on the acrylic plate and cut to equal length. Cut a 2.5" piece of yellow 22 AWG wire and trim a small portion of insulation from both ends and solder it to the group of yellow wires.

10. Insert the single end of the 2.5" piece of yellow wire into the negative (-) port on the acrylic plate terminal block and lightly secure.

11. Cut a 4" piece of white 22 AWG wire, trim off a small portion of insulation from both ends, and insert into the negative (-) port on the acrylic plate terminal block (facing RB1) and fully secure with the yellow wire from Step 10 immediately above. The opposite end will be attached to a Molex connector (Jameco PN: 142201).

12. Cut a 4" piece of black 22 AWG wire, trim off a small portion of insulation from both ends, and insert one end into the positive (+) port on the acrylic plate terminal block (facing RB1), and fully secure. Twist the white wire from Step 11 together with this black wire to make a single bundle. The opposite end will be attached to a Molex connector (Jameco PN: 142201).

13. Take two female pin from the Molex connector kit and solder them on to the ends of the 4" black and white wires. These can be crimped on, but soldering is more secure. The Molex connector has a male and female plastic housing with an index V in the plastic body to correctly mate the connection. Insert these female pins into the male Molex connector housing and note their position with respect to this V. For example, insert the black female pin into the hole closest to the V and the white female pin furthest from the V.

14. This male Molex connector will attach the electronics to the female counterpart on the battery pack later explained in Section K8 below.

d. Begin with PV7 to K1 on RB2 and work towards the terminal block.

1. Bring the strand of 2 wires from PV7 up to K1 on RB2 with a slight catenary arc, cut both wires (save the excess wire), and trim a portion of insulation from both wires coming from PV7.

2. Trim a small portion of insulation one of the wires coming from PV7 and solder it to the gray end of the rectifier diode, close to the diode body because the wire from this end of the diode will eventually insert into the left port on K1.

3. Take one of the excess pieces of wire just cut from PV7, trim off a small portion of insulation from one end, then combine it with the second wire from PV7, and solder it to the black side of the rectifier diode, close to the diode body (Figure 24).

4. Trim off the excess wire from the black end of the diode.

5. Bring the wire soldered to the gray end along the body of the diode.

6. Cut enough length of heat shrink tubing long enough to cover the diode and the soldered joints and apply heat to shrink the tubing to the diode.
7. Trim enough lead on the gray side of the diode so that it will have enough wire to insert into the left port on K1 and then secure in place.
8. Repeat for the remaining pinch valves and for the peristaltic pump.
9. The excess wires coming from the black side of each diode will come together to make the negative return from the pinch valves and pump. Make 2 bundles by braiding the excess wire from PV7, PV8, and PV9 together, and then braid the excess wire from PV4, PV5, and PV6 together, since the flush pinch valve (F) is closest to the electrical terminal block it doesn't need to be grouped. Use some cable ties to tidy up and secure the two braids and bring the group of wires approximately equal to the arc of the acrylic disc and about 2.5" from the terminal block on the acrylic plate and cut to equal length. Cut a 2.5" piece of yellow 22 AWG wire and trim a small portion of insulation from both ends and solder it to the group of yellow wires.
10. Insert the 2.5" piece of yellow wire into the opposite side negative (-) port on the terminal block on the acrylic plate and light secure as there will be 2 more wires connected to this port in later steps.

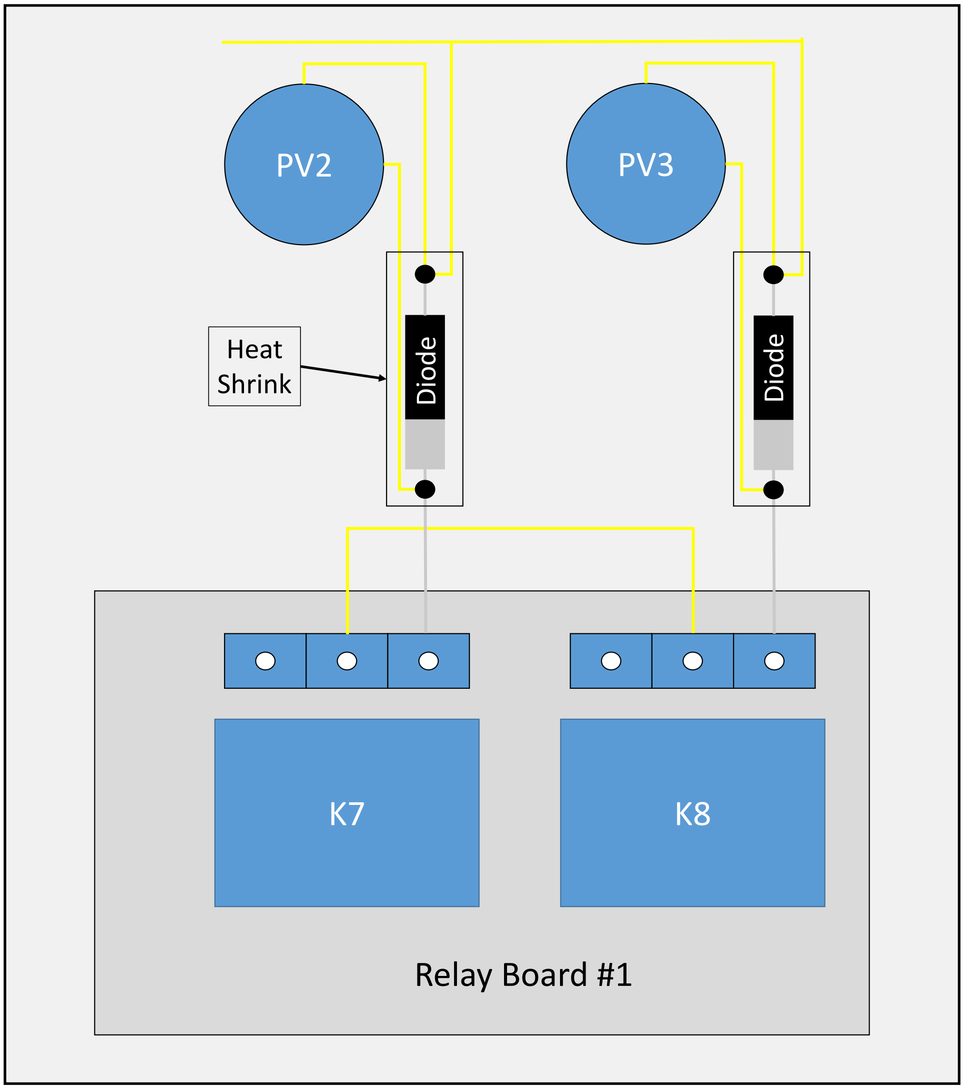

Figure 24: Schematic example of rectifier diode installation for PV2 and PV3, note that this is only an example of 2 (of 8 relays) on one of the two 8-channel relay boards.

2. *APM, RTC, and RB1 & RB2 Wiring:* There are many wires to install to connect the APM to RB1 and RB2, patience and planning are necessary for a correct and secure installation. Colored ribbon jumpers are used to connect the various components. Note that VCC on the APM, RTC, RB1, and RB2 all share the same +5VDC power. RAW and JD-VCC will share the same +12VDC coming from the battery pack. Refer to the schematic wiring diagram in Figure 25 for the following steps.

   a. *+5VDC VCC Connections:* Use 2 purple 8" jumpers to make the +5VDC VCC harness. Cut the two jumpers in half and trim off a small portion of insulation from all four ends. Attach these ends together, solder, and apply heat shrink over the connection. Attach one end of the harness to VCC on the APM, one to the RTC, and the remaining two ends to VCC on the 20 pin buss.

   b. *APM to RTC Connections:*

1. Attach a brown jumper from GND on the APM to GND on the RTC

2. Attach a red jumper from SDA (A4) on the APM to SDA on the RTC

3. Attach an orange jumper from SLC (A5) on the APM to SCL on the RTC.

c. *APM to RB1 and RB3 Connections:* Refer to Table 1 and Figure 25 for connecting the jumpers from the APM to both RB1 and RB2.

3. *RB +12VDC Power Connections:*

a. Use one 8” red jumper, cut it in half, trim off a small piece of insulation from the cut ends, and tin the exposed wires with solder.

b. Connect one red jumper to the JD-VCC pin on RB1 and the other end to the center port terminal on K8 (PV3).

c. Connect the other red jumper to JD-VCC pin on RB2 and the opposite end to the center port terminal on K8 (PV-F).

d. Cut an 8” piece of black 22 AWG wire, trim off a small portion of insulation from each end, insert one end into the positive (+) port on the acrylic plate terminal block (facing RB2) and lightly secure as another wire will be added (Step 4 below). Insert the other end into the center terminal port on RB1, K1 (P) and secure it with the yellow wire already occupying that port.

4. *APM +12VDC Power Connections:*

a. Use one 8” black jumper, cut the connector from one end of the jumper, trim off a small piece of insulation from the cut end, and tin the exposed wire with solder.

b. Place the connector end of the jumper to the RAW pin on the APM and insert the opposite end into the positive (+) port on the acrylic plate terminal block (facing RB2) and secure with the wire from Step 3d above.

5. *RB Ground (-GND) Power Connections:* Use two 8” brown jumper, cut the connector from one end of each jumper, trim off a small piece of insulation from the cut ends, and tin the exposed wires with solder.

a. Use two 8” gray jumpers, cut the connector from one end of each jumper, trim off a small piece of insulation from the cut ends, and tin the exposed wires with solder.

b. Cut a 4” piece of white 22 AWG wire and trim off a small portion of insulation from each end.

c. Attach the two tinned ends of the gray wires to one end of the 4” white wire, solder and cover with heat shrink.

d. Connect one gray jumper to the GND pin (on the JD-VCC, VCC, GND buss) on RB1.

e. Connect the other gray jumper to the GND pin (on the JD-VCC, VCC, GND buss) on RB2.

f. Insert the other end of the 4” white wire into the negative (-) port on the acrylic plate terminal block (facing RB2) and lightly with the yellow wire for the pinch valves on RB2.

6. *APM Ground (-GND) Power Connections:*

c. Use one 8” white jumper, cut the connector from one end of the jumper, trim off a small piece of insulation from the cut end, and tin the exposed wire with solder.

d. Place the connector end of the jumper to the GND pin on the APM and insert the opposite end into the negative (-) port on the acrylic plate terminal block (facing RB2) and secure with the yellow wire from the pinch valves on RB2 and the white wire from Step 5f above.

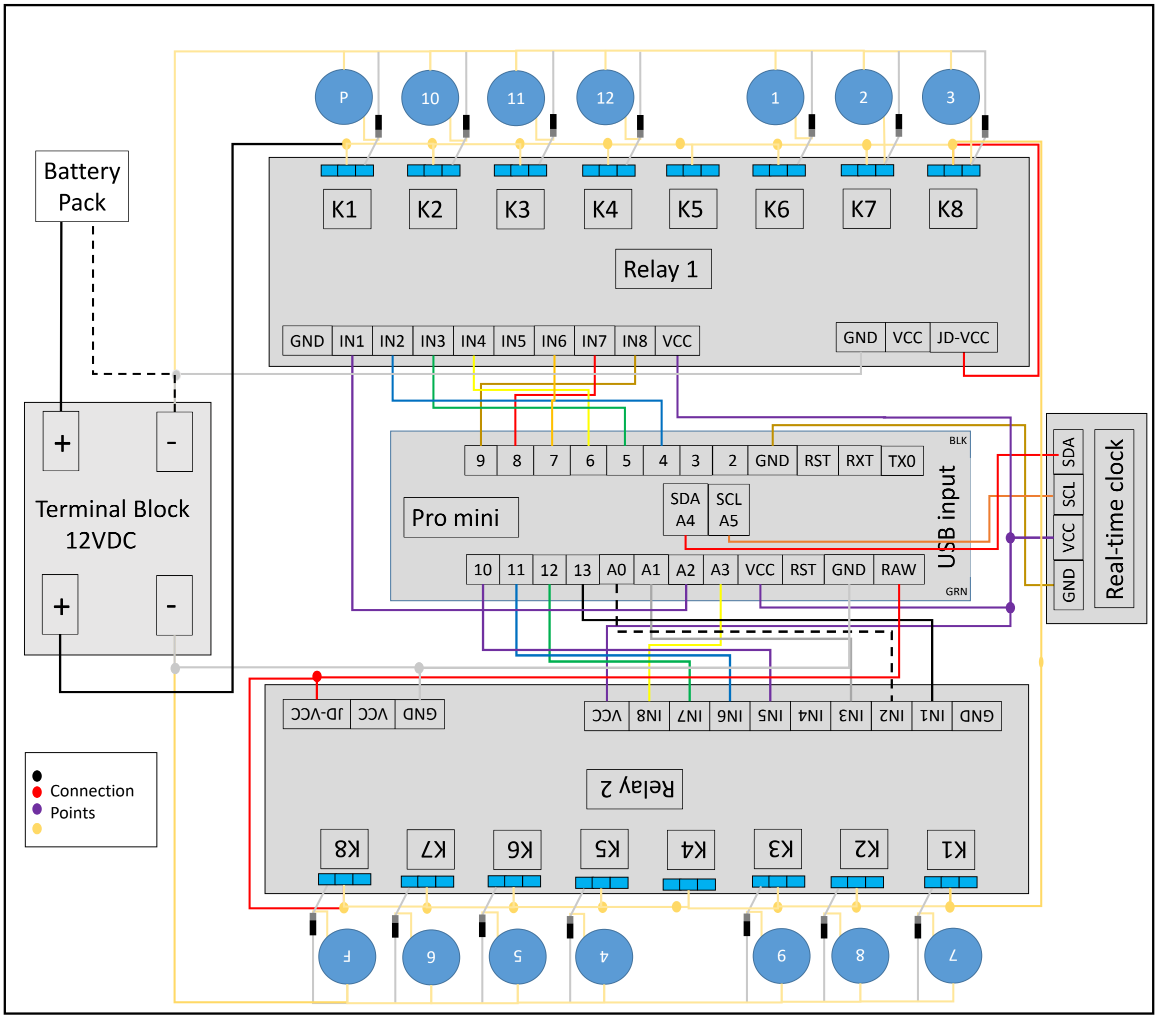

Figure 25: Wiring Schematic for the autonomous multiport water sampler.

K. **12VDC Battery Pack:** There are many ways to supply 12VDC battery power. We will focus on one design using 8 D-cell 1.5V alkaline batteries assembled in series and shrink wrapped to make 12VDC at 15Hh (Digikey PN: P643-F024-ND) and then link 4 of these in parallel to make a 12VDC at 60Ah battery pack (Figure 26 and 27).

1. The mounting plate is made from laser cut 7” OD x 1/8” thick clear acrylic from Delvies Plastics. Mark on the acrylic disc TOP, UP, DOWN, LEFT, and RIGHT.

   a. Find the center of the acrylic disc and make two perpendicular reference lines across the disk as shown with the red dashed line in Figure 26. These will be your center reference lines for making all subsequent blue reference lines also shown in Figure 26 and 27.

   b. Measure out, left and right, from the center point along the horizontal center line 1.125” and make a vertical reference line at each mark (Figure 26).

   c. Measure up and down from the vertical center line 0.8125” and make a horizontal reference line at each mark. The intersections of these four reference lines will be the location of the ¼-20 threaded rod holes (Figure 26).

   d. Drill four 0.2656” (17/64”) diameter holes indicated in Figure 26 as green circles.

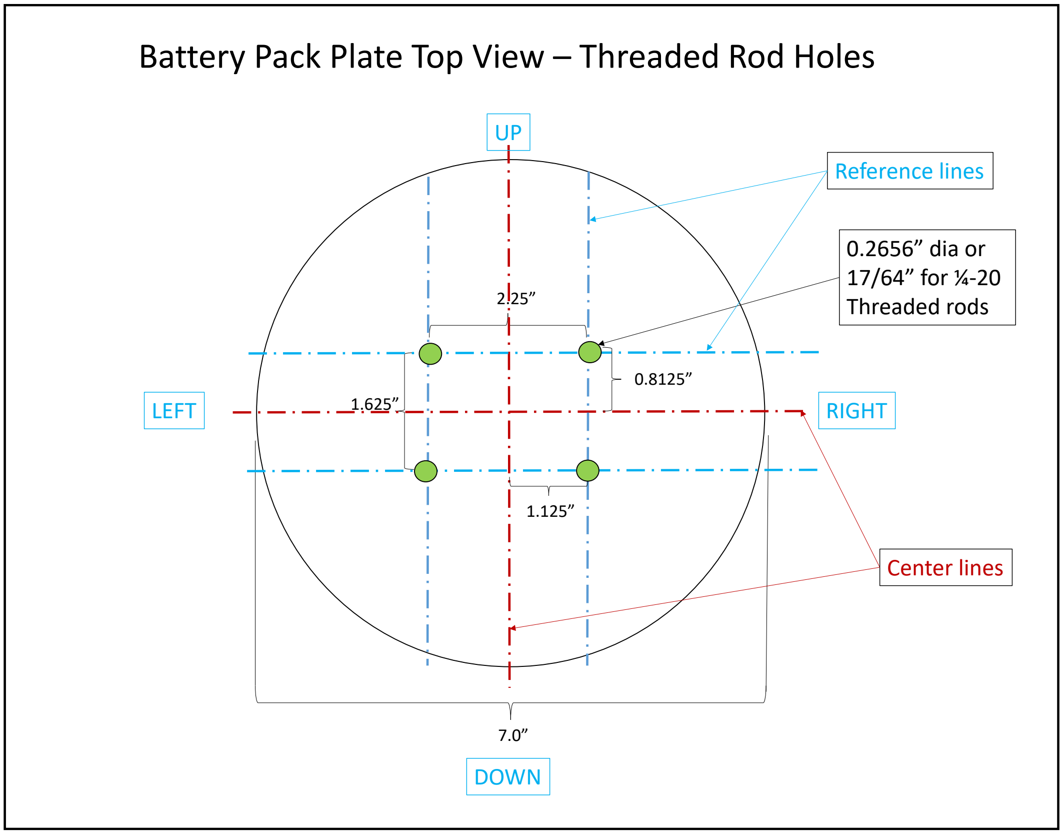

Figure 26: Schematic of the acrylic disc used for the battery pack mounting plate.

   e. To add the DC to DC boost converter module XL6009 to the battery pack use the specifications on Figure 27. It is the same as Figure 26, but adds two additional holes to accept the 2-56” x ¼” nylon standoffs and the XL6009 module.

   f. Measure up from the horizontal red center line 2.0” and 2.625” and make two parallel horizontal reference lines (Figure 27).

g. Measure left and right from the vertical red center line 0.59375” (19/32”) and make two parallel reference lines (Figure 27).

h. Where the two reference lines from Steps K1f and g intersect will be the location of the mounting holes for the XL6009 module shown as green dots in Figure 27.

i. Drill two 0.59375 (19/32”) diameter holes indicated on Figure 27 as green circles.

j. At the center where the two red center lines intersect drill a 0.109375” (7/64”) diameter hole for the two port terminal block shown as a blue dot on Figure 27.

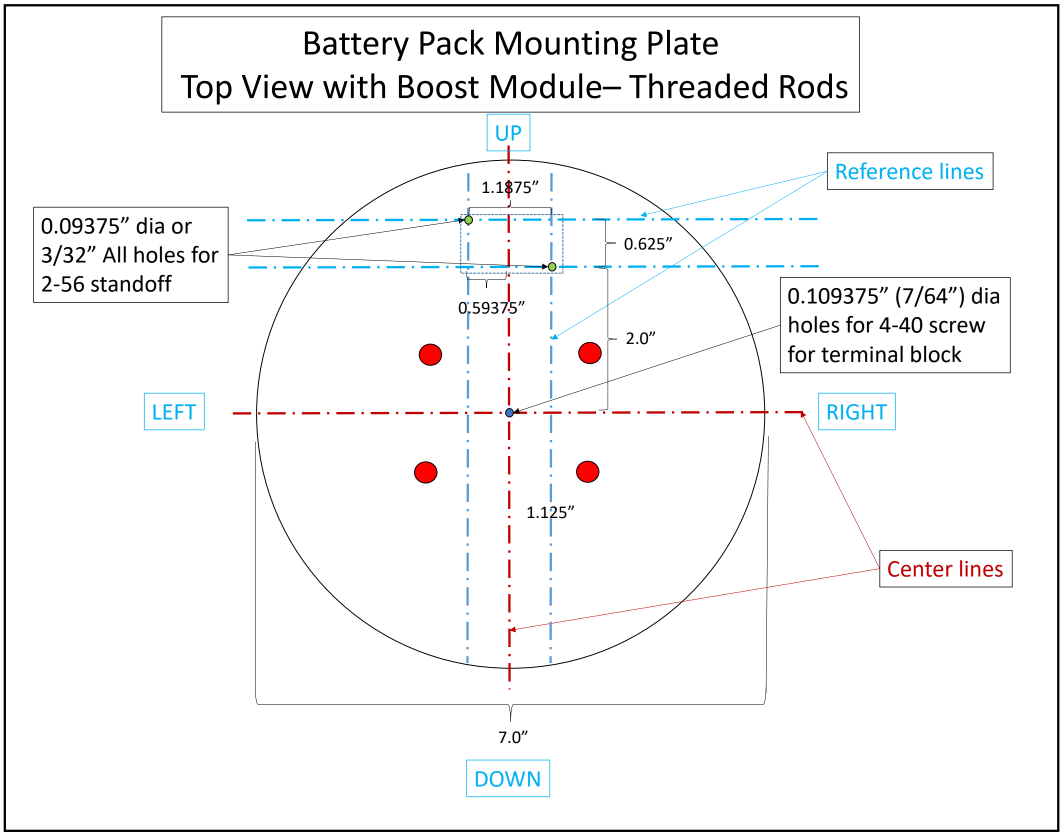

Figure 27: Schematic of the acrylic disc used for the battery pack mounting plate and the DC to DC boost converter XL6009 module. The location is indicated by a dashed rectangle. The greed dots show the location of the standoffs to secure the module. The blue dot is for the terminal block.

2. Attach a 2-port electrical terminal block (Jameco PN: 215011) to the acrylic disc at the location indicated on Figure 27 by a blue dot using a 4-40 x 5/8” long stainless steel screws (McMaster PN: 91772A112) and stainless steel nut (McMaster PN: 90257A005). **Note:** The terminal block comes with 8 ports. Use a utility knife to slice off 2 ports.

3. Attach 2 nylon 2-56 x ¼” long standoffs (McMaster PN: 92745A370) to the acrylic disc using 2-56 x ¼” long stainless steel screws (McMaster PN: 94735A707) at the location of the green circles on Figure 27.

4. Connect the four 8 D-cell battery packs (Digikey PN: P643-F024-ND) in parallel as shown in Figure 28.

5. Insert two ¼-20 x 6" long threaded rods (McMaster PN: 95412A564) and thread them into two opposite facing tapped holes in the battery end cap for the pressure case housing. Lock them in place with a stainless steel washer and nut (McMaster PN: 94804A029 and 90107A029).

6. Place half of the battery pack on one side of the rods and the other half on the other. Bring the cells together and using electrical tape, wrap the battery pack tight against the rods.

7. Thread on two stainless steel nuts (McMaster PN: 94804A029) and place a stainless steel washer McMaster PN: 90107A029) on top of the nuts. Thread the nuts down until they are approximately 1/8" below the top of the battery pack

8. Cut a two 5", two 6", and two 24" long piece of white and black 22 AWG wire and trim off a small portion of insulation from both ends of the 6 wires.

9. Solder one end of the 6" black wire to the positive (+) post of the battery pack and one end of the 6" white wire to the negative (-) post on the battery pack. To make a single bundle by braiding the black and white 6" wires together then cut to equal length and re-trim if necessary. Make sure the ends do not touch. Tape can be temporarily applied to protect the ends for touching.

10. Solder the black 5" long black wire to the positive (+) IN terminal and the white 5" long wire to the negative (-) IN terminal on the XL6009 module (Amazon PN: XL6009). Make a single bundle by braiding the black and white 5" wires together and cut to equal length if not even and trim the ends if needed.

11. Solder the black 24" long black wire to the positive (+) OUT terminal and the white 24" long wire to the negative (-) OUT terminal on the XL6009 module (Amazon PN: XL6009). Make a single bundle by braiding the black and white 24" wires together and cut to equal length and re-trim if necessary.

12. At the other end of the 24" black and white wire bundle a Molex connector (Jameco PN: 142201) will be attached. Take two male pin from the Molex connector kit and solder them to other end of the 24" black and white wires. These can be crimped on, but soldering is more secure. As explained in Section J1-c14 above The Molex connector has a male and female plastic housing with an index V in the housing to correctly mate the connection. Insert these male pins into the female plastic Molex connector housing and note their position with respect to this V. Keeping with the same example, insert the black male pin into the hole closest to the V and the white male pin furthest from the V. **Important note of caution**: Check to be certain that the black and white wire inserted into the plastic male and female Molex housings align across for each other when indexing with the V on the plastic connector. This connector will allow you to safely connect and disconnect the battery power as needed, but <u>do not connect them at this time</u>. Refer to the users manual for additional information.

13. Now attach the XL6009 module to the acrylic disc by placing it onto the 2-56 standoffs and secure using two 2-56 nylon nuts (McMaster PN: 94812A100). Attach the two 5" long black

and white wires to the 2-port terminal block on the acrylic disc. This will complete the acrylic battery pack disc.

14. Place the completed battery pack acrylic disk on top of the battery pack and secure using stainless steel washers and nuts (McMaster PN: 94804A029 and 90107A029).

15. Remove the tape from the black and white wire leads on the battery pack and insert them into the 2-port terminal block on the battery pack acrylic disc making sure the black and white wires align across from each other and secure.

16. There is a blue potentiometer on the XL6009 that can be used to adjust the output DC voltage. Use a digital multi-meter (DMM) to check and note the incoming battery voltage. Now attach and DMM to the end of the Molex connector to check and note the output voltage from the XL6009. Use a small screw driver to adjust the output voltage to be 12 VDC. This output voltage check should be performed using a DMM after each battery pack change.

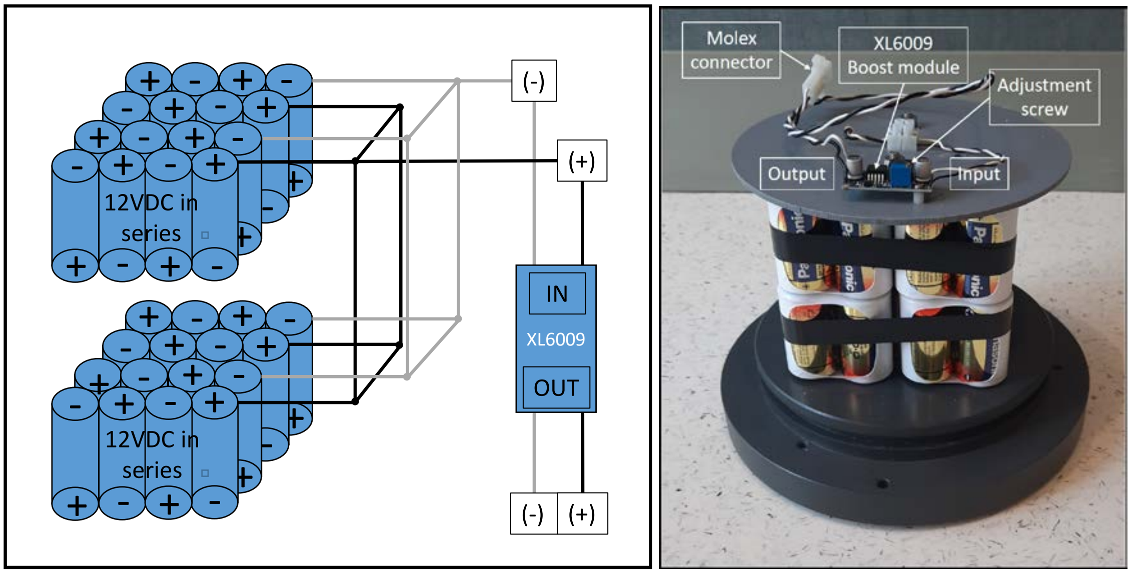

Figure 28: Schematic 12VDC alkaline battery pack (left) for the autonomous underwater pumping system with actual alkaline battery pack (right) attached to end cap.

L. Pressure Case Housing Assembly: To seal the end caps, Viton o-rings (McMaster: 9464K681) are lightly greased with silicone o-ring grease and inserted into the groove on both end caps. This assembly procedure is covered in the user manual.

1. To install the end cap, make sure that the USB communications cable (top end cap) and battery power wires (bottom end cap) are clear, align the holes in the end cap with the holes on the end cap ring, press the end cap into place, and secure with two ¼-20 x 1-3/4" long stainless steel bolts (McMaster PN: 93190A547) and flat washers (McMaster PN: 90107A029). These same two ¼-20 x 1-3/4" bolts and washers will be used as jack bolts to remove the end caps. Keep a third ¼-20 bolt and washer on hand, or use one from the other end cap.

2. Install three smaller ¼-20 x ½” long stainless steel bolts (McMaster PN: 93190A537) with flat washers (McMaster PN: 90107A029) into the three inner ¼-20 threaded holes. These bolts will serve to protect the jack bolt holes from being clogged.

3. Repeat Steps 1 and 2 above for the other end cap.

4. Once both end caps are secured install the stainless steel purge plug (McMaster PN: 51205K288) into the bottom end cap.

5. For anti-foul protection PVC tape (McMaster PN: 6029T96) can be used to cover the body and end caps of the pressure case housing.
