# Supplementary material for: Autonomous submersible multiport water sampler
Source: HardwareX. 2021 Apr 22;9:e00197. doi: 10.1016/j.ohx.2021.e00197 (PMC9041238; doi:10.1016/j.ohx.2021.e00197)
Supplement: Supplementary data 3 [file mmc3.pdf]

Designator	Component	Cost/ea - USD	Qty needed	Total Cost - USD	Vendor	Material type	Comments
Electronics	FTDI TTL-232R-3V3 USB - TTL Level Serial Converter Cable, PN: DEV-09718	$17.95	1	$17.95	sparkfun.com	Non-specific Other	USB cable to communicate with Arduino pro mini
Electronics	8-channel 5V relay, PN: 101-70-102	$9.99	2	$19.98	sainsmart.com	Non-specific Other	Control pumps - mechanical relay
Electronics	Arduino pro mini, PN: DEV-11113	$9.95	1	$9.95	sparkfun.com	Non-specific Other	Computer interface
Electronics	Adafruit DS3231 Precision RTC Breakout, PN: P3013	$14.95	1	$14.95	adafruit.it	Non-specific Other	Real-time clock for Arduino pro mini interface
Electronics	Lithium battery, PN: CR1220	$1.99	1	$1.99	amazon.com	Non-specific Other	Battery for real-time clock
Electronics	Polyurethane Foam Mounting Tape, Open-Cell, 1/4" Thick, 1/2" Wide, 15 Feet Long, Off-White, PN: 7626A272	$24.14	1	$24.14	mcmaster.com	Polyurethane	Mount Arduino Pro mini to acrylic disc
Electronics	Female-female jumper wires 8", AWG 26 Pack of 40, PN: 2260746	$4.95	1	$4.95	amazon.com	Non-specific Other	Wires for Arduino pro mini interface, relay, and real-time clock
Electronics	break away header pins (40) straight, PN: PRT-00116	$1.50	1	$1.50	sparkfun.com	Non-specific Other	Pin connections for Arduino pro mini interface - USB connection
Electronics	Rectifier diode, 1N4004, PN: 2368-1N4004-ND	$0.10	13	$1.30	digikey.com	Non-specific Other	Rectifier diode for pinch valves to prevent electrical backfeed
Electronics	break away header pins (40) 90 deg, PN: PRT-00553	$1.95	1	$1.95	sparkfun.com	Non-specific Other	Pin connections for Arduino pro mini interface - USB connection
Pump & Manifold	Passivated 18-8 Stainless Steel Pan Head Phillips Screw 4-40 Thread, 3/8" Long, PN: 91772A108	$0.05	6	$0.30	mcmaster.com	Stainless steel	Secure relay plate to pinch valves
Electronics	Laser Cut Clear Acrylic Disks, 1/8" Thick Disk Size: 7" Diameter, PN: cut to order	$4.25	2	$8.50	delviesplastics.com	Acrylic	Electronics mounting discs

Designator	Component	Cost/ea - USD	Qty needed	Total Cost - USD	Vendor	Material type	Comments
Electronics	8mm terminal block, PN: 215011	$1.95	1	$1.95	Jameco.com	Nylon	Terminal bock
Electronics	Heat shrink tubing, assortment pack, 4" black, PN: TT74901	$17.95	1	$17.95	Jameco.com	Non-specific Other	Heat shrink for covering wire splices place of crimp connectors
Electronics	22AWG 6-Color Tinned-Copper Wire Assortment, PN: 2153705	$18.95	1	$18.95	Jameco.com	Non-specific Other	Wire for electronics
Electronics	Passivated 18-8 Stainless steel pan head screws Phillips, 2-56 Thread, 5/16" Long, PN: 91772A078	$0.05	6	$0.30	mcmaster.com	Stainless steel	For electronics standoffs on top of relay boards
Electronics	PNylon Hex Nut, 2-56 Thread Size, PN: 94812A100	$0.07	5	$0.35	mcmaster.com	Stainless steel	For electronics standoffs on relay boards
Electronics	Nylon 6/6 Plastic Hex Standoff, 3/16" Hex, 3/4" Long, 2-56 Female Thread, PN: 92319A275	$2.01	6	$12.06	mcmaster.com	Nylon	Electronics standoffs - above relays
Electronics	Off-White Nylon Pan Head Screws Phillips, 2-56 Thread, 1/4" Long, PN: 94735A707	$0.06	14	$0.84	mcmaster.com	Nylon	For electronics standoffs
Electronics	Male-Female Threaded Hex Standoff, Nylon 6/6, 3/16" Hex Size, 1/4" Long, 2-56 to 2-56 Size Thread, PN: 92745A370	$2.89	11	$31.79	mcmaster.com	Nylon	Electronics standoffs - below relays
Pressure Housing	Super-Corrosion-Resistant 316 Stainless Steel Hex Head Screw, 1/4"-20 Thread Size, 1-3/4" Long, Fully Threaded, PN: 93190A547	$0.52	6	$3.12	mcmaster.com	Stainless steel	End cap jack bolts - used to separate end cap from housing

Designator	Component	Cost/ea - USD	Qty needed	Total Cost - USD	Vendor	Material type	Comments
Pressure Housing	*Standard-Wall Unthrded Rigid PVC Pipe for Water, 8 Pipe Size, 5 Feet Long PN: 48925K26*	*$82.59*	*1*	*$82.59*	*mcmaster.com*	*PVC*	*Pressure case housing stock*
Pressure Housing	*Chemical-Resistant PVC Sheet, 12" x 12" x 1", PN: 8748K118*	*$45.73*	*2*	*$91.46*	*mcmaster.com*	*PVC*	*Pressure case housing ring*
Pressure Housing	*Chemical-Resistant PVC Sheet, 12" x 12" x 3", PN: 8747K401*	*$228.40*	*2*	*$456.80*	*mcmaster.com*	*PVC*	*Pressure case end caps*
Pressure Housing	*Floor Marking Tape, 2" Wide, 180 Feet Long, PN: 6029T96*	*$13.44*	*1*	*$13.44*	*mcmaster.com*	*PVC*	*Tape for anti-fouling or securing pumps to housing*
Pressure Housing	*Chemical-Resistant Viton® Fluoroelastomer O-Ring 1/4 Fractional Width, Dash Number 443, PN: 9464K681*	*$14.71*	*2*	*$29.42*	*mcmaster.com*	*Viton*	*End cap o-rings*
Pressure Housing	*Pipe Cement for Plastic Pipe for 12" Maximum Diameter PVC Plastic Pipe, 8oz, PN: 74605A15*	*$6.38*	*1*	*$6.38*	*mcmaster.com*	*Viton*	*PVC cement*
Pressure Housing	*Primer for PVC Pipe Cement for Plastic Pipe, Clear, 8oz, PN: 18815K51*	*$6.07*	*1*	*$6.07*	*mcmaster.com*	*Viton*	*PVC primer*
Pressure Housing	*8 oz. Cleaner for PVC Pipe Cement for Plastic Pipe, 8oz, PN: 74605A44*	*$5.38*	*1*	*$5.38*	*mcmaster.com*	*Viton*	*PVC cleaner*
Pressure Housing	*Super-Corrosion-Resistant 316 Stainless Steel Hex Head Screw 1/4"-20 Thread Size, 1/2" Long, Fully Threaded, PN: 93190A537*	*$0.19*	*6*	*$1.14*	*mcmaster.com*	*Stainless steel*	*End cap jack bolts - used to separate end cap from housing*

Designator	Component	Cost/ea - USD	Qty needed	Total Cost - USD	Vendor	Material type	Comments
Pressure Housing	*Extreme-Pressure 316 Stainless Steel Pipe Fitting, Plug with External Hex Drive, 1/2"-20 UNF, PN: 51205K288*	*$15.08*	*1*	*$15.08*	*mcmaster.com*	*Stainless steel*	*Pressure release plug*
Pump & Manifold	*Thomas peristaltic pump, SR10-50 series PN: AV 2050-0503*	*$84.00*	*1*	*$84.00*	*gd-thomas.com*	*Non-specific Other*	*Thomas 220ml/min peristaltic pump*
Pump & Manifold	*Passivated 18-8 Stainless Steel Pan Head Phillips Screw 4-40 Thread, 5/8" Long, PN: 91772A112*	*$0.05*	*2*	*$0.05*	*mcmaster.com*	*Stainless steel*	*Secure peristaltic pump to acrylic plate*
Pump & Manifold	*Stainless steel hex nut, 316, 4-40 thread, PN: 90257A005*	*$0.06*	*2*	*$0.05*	*mcmaster.com*	*Stainless steel*	*Secure peristaltic pump to acrylic plate*
Pump & Manifold	*Right-Angle Flow Rectangular Manifold, Polypropylene, 6 Outlets, 1/4 NPT Inlet x 1/8 NPT Outlet, PN: 5364K231*	*$35.25*	*1*	*$35.25*	*mcmaster.com*	*Polypropylene*	*6-port manifold*
Pump & Manifold	*Plastic Barbed Tube Fittings for Air & Water Tight-Seal, Inline Tee, 1/8" Tube ID x 1/8 NPT Male, PN: 5463K55*	*$1.48*	*6*	*$8.88*	*mcmaster.com*	*Nylon*	*Valve ports on 6-port manifold*
Pump & Manifold	*Plastic Barbed Tube Fittings for Air & Water Tight-Seal, Elbow, for 1/8" Tube ID x 1/4 NPT Male, PN: 5463K133*	*$0.69*	*2*	*$1.38*	*mcmaster.com*	*Nylon*	*In/Out ports on 6-port manifold*
Pump & Manifold	*Plastic Barbed Tube Fittings for Air & Water Tight-Seal, Adapter, for 1/8" Tube ID x 1/8 NPT Male, PN: 5463K438*	*$1.53*	*13*	*$19.89*	*mcmaster.com*	*Nylon*	*Internal end cap fittings for valves and purge port*

Designator	Component	Cost/ea - USD	Qty needed	Total Cost - USD	Vendor	Material type	Comments
Pump & Manifold	*Plastic Barbed Tube Fitting for Air and Water, Tight-Seal, Elbow, 3/16" ID x 1/8 NPT Male, White, PN: 2974K208*	*$0.96*	*1*	*$0.96*	*mcmaster.com*	*Nylon*	*Connect peristaltic pump tubing to end cap intake port*
Pump & Manifold	*Passivated 18-8 Stainless Steel Pan Head Phillips Screw 6-32 Thread, 1-1/4" Long, PN: 91772A155*	*$0.83*	*2*	*$1.66*	*mcmaster.com*	*Stainless steel*	*Secure 6 port manifold*
Pump & Manifold	*18-8 Stainless Steel Hex Nut, 6-32 Thread Size, PN: 91841A007*	*$0.03*	*2*	*$0.06*	*mcmaster.com*	*Stainless steel*	*Secure 6 port manifold*
Pump & Manifold	*Plastic Barbed Tube Fitting for Air and Water Tight-Seal, Reducer, 3/16" x 1/8" ID, Semi-Clear White, PN: 5463K626*	*$0.82*	*1*	*$0.82*	*mcmaster.com*	*Nylon*	*Reducer to connect peristaltic pump to inlet port tubing*
Pump & Manifold	*Silicone tubing, 1/8" ID x 1/4" OD, 5 ft long, PN: SIH1-0804-NAS-005*	*$46.14*	*1*	*$46.14*	*clippard.com*	*Silicone*	*Reducer to connect peristaltic pump to inlet port tubing*
Battery Pack	*Laser Cut Clear Acrylic Disks, 1/8" Thick Disk Size: 7" Diameter, PN: cut to order*	*$4.25*	*1*	*$4.25*	*delviesplastics.com*	*Acrylic*	*Battery holder*
Battery Pack	*8 D cell 12V battery pack, PN: P643-F024-ND*	*$18.95*	*4*	*$75.80*	*digikey.com*	*Non-specific Other*	*Batteries/shrink wrapped*
Battery Pack	*316 Stainless Steel Washer for 1/4" Screw Size, 0.281" ID, 0.625" OD, PN: 90107A029*	*$0.08*	*4*	*$0.33*	*mcmaster.com*	*Stainless steel*	*To secure acrylic disc and battery packs to rods on end cap*
Battery Pack	*Super-Corrosion-Resistant 316 Stainless Steel Hex Nut, 1/4"-20 Thread Size, PN: 94804A029*	*$0.80*	*6*	*$4.80*	*mcmaster.com*	*Stainless steel*	*To secure acrylic disc and battery packs to rods on end cap*

Designator	Component	Cost/ea - USD	Qty needed	Total Cost - USD	Vendor	Material type	Comments
Battery Pack	*18-8 Stainless Steel Threaded Rod, 1/4"-20 Thread Size, 6" Long, PN: 95412A564*	*$1.15*	*2*	*$2.30*	*mcmaster.com*	*Stainless steel*	*Rods for securing heat shrink Digikey battery packs*
Battery Pack	*Connector Kit 1490Series 4Pin .093 12Amp 250VAC 18-22AWG, PN: 142201*	*$5.49*	*1*	*$5.49*	*Jameco.com*	*Nylon*	*Battery plug connection*
Battery Pack	*8mm terminal block, PN: 215011*	*$1.95*	*1*	*$1.95*	*Jameco.com*	*Nylon*	*Terminal bock*
Battery Pack	*Passivated 18-8 Stainless Steel Pan Head Phillips Screw, 4-40 Thread, 5/8" Long, PN: 91772A112*	*$0.05*	*1*	*$0.05*	*mcmaster.com*	*Stainless steel*	*Secure terminal block to acrylic plate*
Battery Pack	*Stainless steel hex nut, 316, 4-40 thread, PN: 90257A005*	*$0.06*	*1*	*$0.05*	*mcmaster.com*	*Stainless steel*	*Secure terminal block to acrylic plate*
Battery Pack	*EBOOT XL6009 DC boost converter module PN: XL6009*	*$12.25*	*1*	*$12.25*	*amazon.com*	*Non-specific Other*	*DC to DC boost converter module XL6009*
Battery Pack	*PNylon Hex Nut, 2-56 Thread Size, PN: 94812A100*	*$0.07*	*2*	*$0.14*	*mcmaster.com*	*Stainless steel*	*For standoffs on XL6009 module*
Battery Pack	*Off-White Nylon Pan Head Screws Phillips, 2-56 Thread, 1/4" Long, PN: 94735A707*	*$0.06*	*2*	*$0.12*	*mcmaster.com*	*Nylon*	*For standoffs on XL6009 module*
Battery Pack	*Male-Female Threaded Hex Standoff, Nylon 6/6, 3/16" Hex Size, 1/4" Long, 2-56 to 2-56 Size Thread, PN: 92745A370*	*$2.89*	*2*	*$5.78*	*mcmaster.com*	*Nylon*	*Electronics standoffs - XL6009 module*
Battery Pack	*8 D cell battery holder, PN: BH48DW-ND*	*$6.96*	*4*	*$27.84*	*digikey.com*	*Non-specific Other*	***Optional*** *- Battery holders for Battery Pack*

Designator	*Component*	*Cost/ea - USD*	*Qty needed*	*Total Cost - USD*	*Vendor*	*Material type*	*Comments*
Battery Pack	*18-8 Stainless Steel Threaded Rod 1/4"-20 Thread Size, 7" Long, PN: 95412A566*	*$1.81*	*2*	*$3.62*	*mcmaster.com*	*Stainless steel*	***Optional*** *- Rods for securing battery holders for individual D-cells*
Battery Pack	*18-8 Stainless Steel Hex Head Screw 1/4"-20 Thread Size, 1/2" Long, PN: 92240A537*	*$0.08*	*2*	*$0.16*	*mcmaster.com*	*Stainless steel*	***Optional*** *- Bolts to prevent battery packs from shifting*
Battery Pack	*8 D cell 12V battery pack, 15.25Ah PN: SY632-F024-ND*	*$17.74*	*4*	*$70.96*	*digikey.com*	*Non-specific Other*	*Batteries/shrink wrapped (* ***alternative*** *)*
Valve Assembly	*Pinch valve, 1/4", PN: NPV3-1C-05-12*	*$107.05*	*13*	*$1,391.65*	*clippard.com*	*Non-specific Other*	*Clippard 1/4" sample and purge pinch valves*
Valve Assembly	*Laser Cut Clear Acrylic Disks, 1/8" Thick Disk Size: 7" Diameter, PN: cut to order*	*$4.25*	*2*	*$8.50*	*delviesplastics.com*	*Acrylic*	*Electronics mounting discs*
Pump & Manifold	*Cable ties 4.0" x 0.10" PN: 126544*	*$0.04*	*20*	*$0.80*	*Jameco.com*	*Nylon*	*Cable ties to secure wires*
Pump & Manifold	*Passivated 18-8 Stainless Steel Pan Head Phillips Screw, 4-40 Thread, 3/8" Long, PN: 91772A108*	*$0.05*	*26*	*$1.30*	*mcmaster.com*	*Stainless steel*	*Secure Clippard pinch valves, pump, and relay plate to disc*
Valve Assembly	*Passivated 18-8 Stainless Steel Pan Head Phillips Screw, 10-32 Thread, 1/2" Long, PN: 91772A829*	*$0.92*	*4*	*$3.68*	*mcmaster.com*	*Stainless steel*	*Secure valve disc to standoffs*
Valve Assembly	*Male-Female Threaded Hex Standoff, Aluminum, 3/8" Hex Size, 2-1/2" Long, 10-32 Size Thread, PN: 93505A031*	*$1.92*	*4*	*$7.68*	*mcmaster.com*	*Aluminum*	*Valve assembly standoffs*

Designator	Component	Cost/ea - USD	Qty needed	Total Cost - USD	Vendor	Material type	Comments
End Cap fittings	*Tight-Seal Moisture-Resistant Barbed Tube Fitting, Plastic, for 3/16" Tube ID x 1/4"-28 Thread Male Pipe, PN: 5047K119*	*$0.73*	*2*	*$1.46*	*mcmaster.com*	*Acetal*	*In and out ports - end cap external fittings*
End Cap fittings	*Plastic Quick-Turn Tube Coupling Plugs, 1/4"-28 UNF Male Thread Size, Nylon, PN: 51525K221*	*$5.69*	*12*	*$68.28*	*mcmaster.com*	*Nylon*	*End cap external valve fittings*
End Cap fittings	*Plastic Quick-Turn Tube Coupling Sockets, for 1/8" Barbed Tube ID, Nylon, PN: 51525K213*	*$3.78*	*12*	*$45.36*	*mcmaster.com*	*Nylon*	*Fitting to connect tubing to external end cap valve fittings*
End Cap fittings	*Plastic Quick-Turn Tube Coupling Nylon Caps for Sockets, PN: 51525K315*	*$3.78*	*12*	*$45.36*	*mcmaster.com*	*Nylon*	*Caps for external valve end cap ports*
