# Supplementary material for: Autonomous submersible multiport water sampler
Source: HardwareX. 2021 Apr 22;9:e00197. doi: 10.1016/j.ohx.2021.e00197 (PMC9041238; doi:10.1016/j.ohx.2021.e00197)
Supplement: Supplementary data 4 [file mmc4.pdf]

Autonomous Underwater Multiport Water Sampler (AutoSampler)

This document contains all of the drawings and schematics to build the AutoSampler. Section 1 is focused on the pressure case housing designs, Section 2 on the Acrylic disc designs for the electronics, pump, and battery pack, and Section 3 contains the wiring schematics.

Section 1: Pressure Case Housing

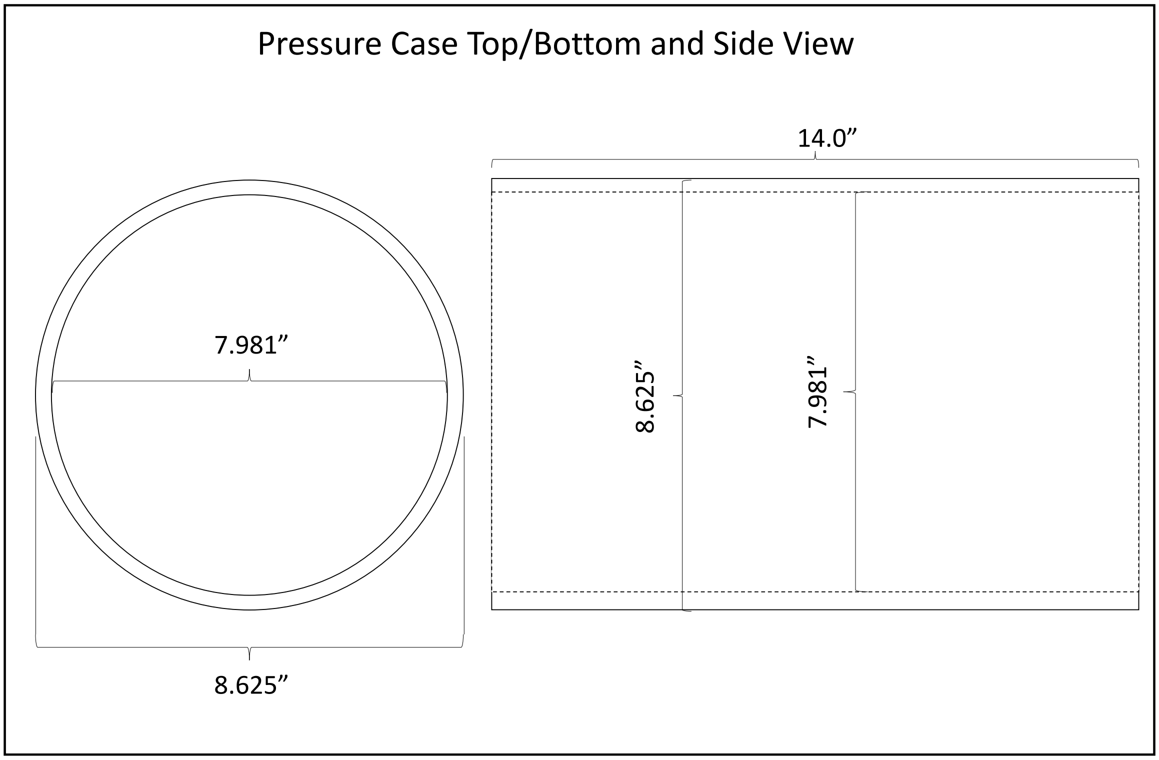

Pressure case housing body, top and side views.

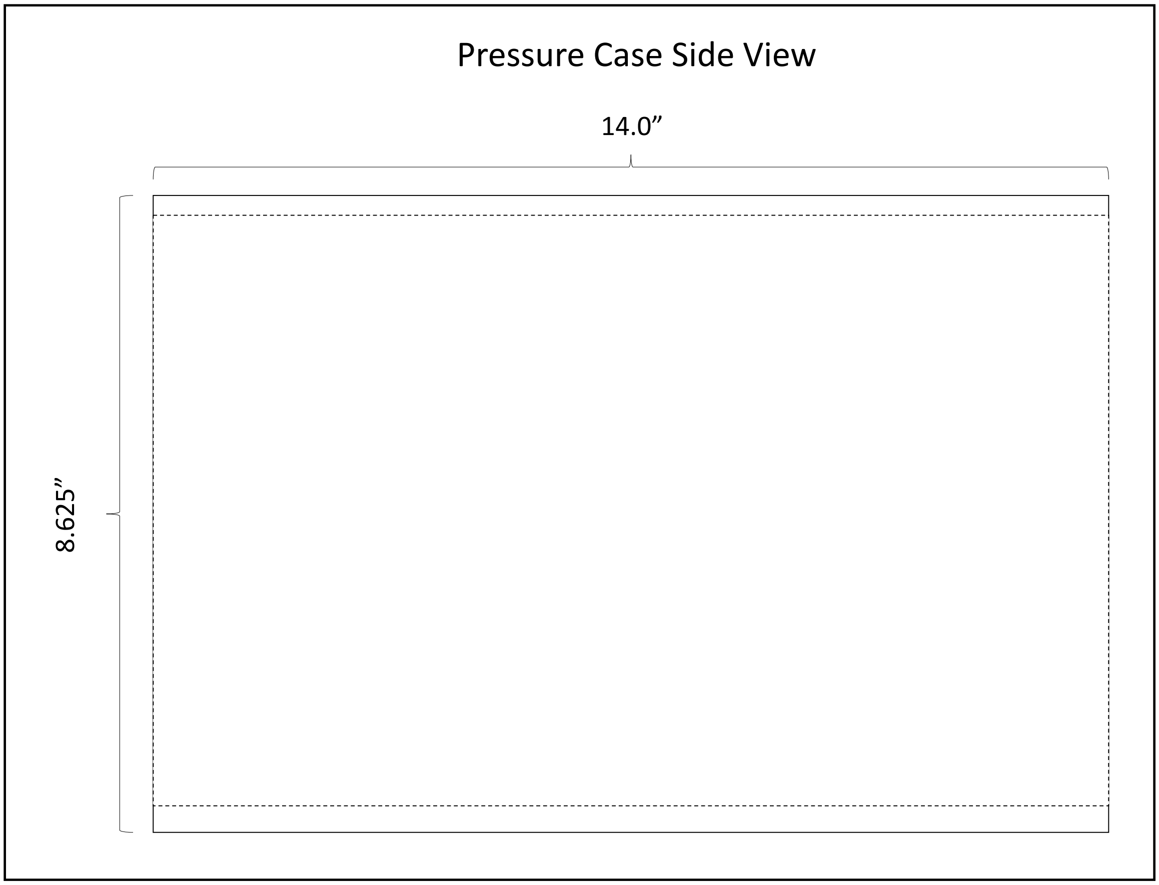

Pressure case housing body, side view.

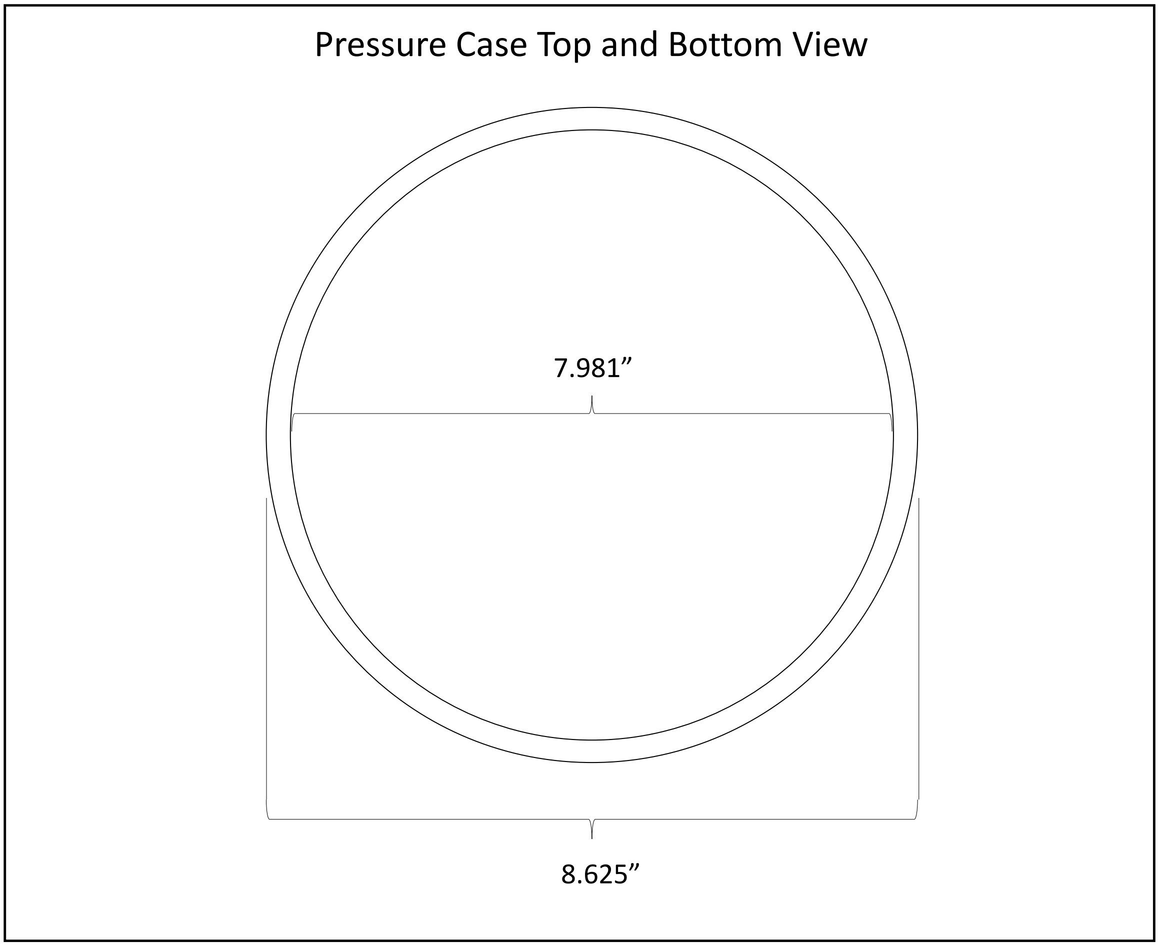

Pressure case housing body, top and bottom views.

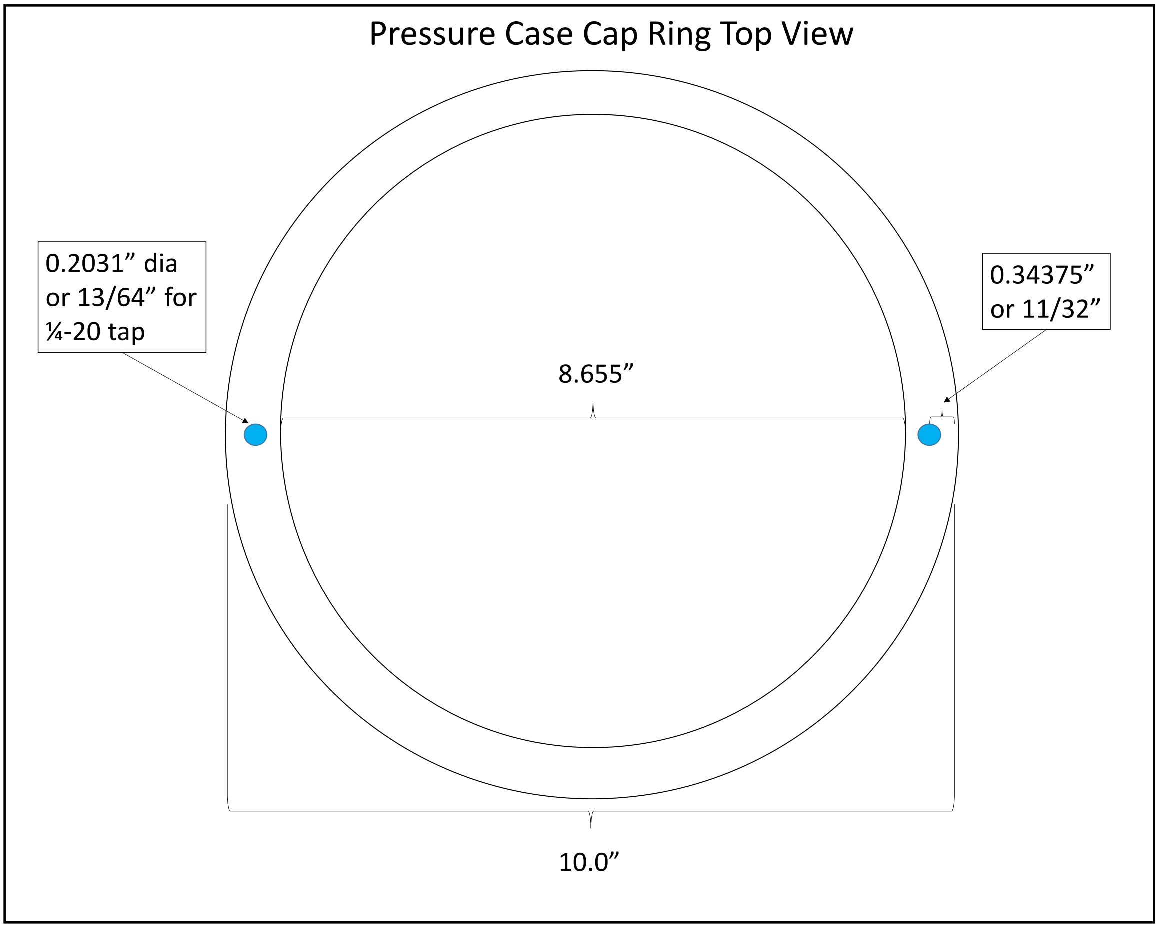

Pressure case housing cap ring, top view

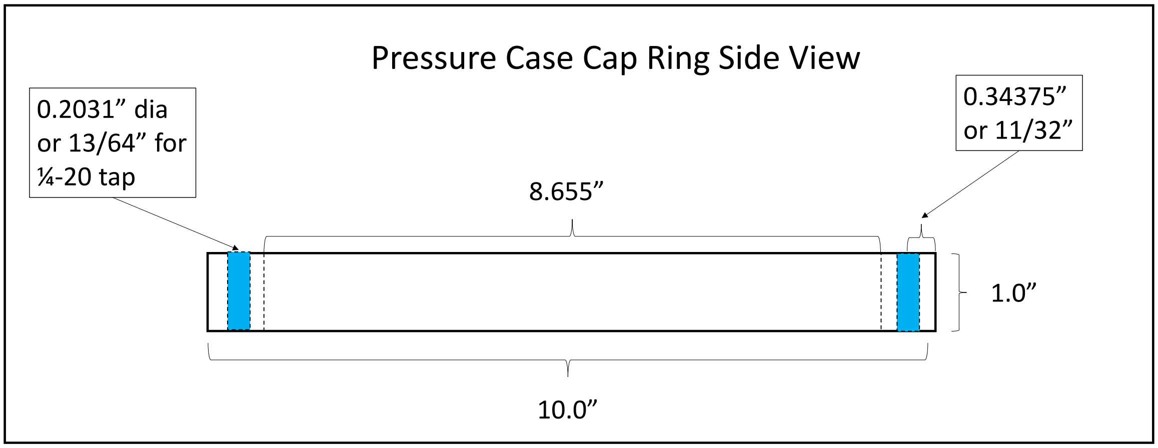

Pressure case housing cap ring, side view

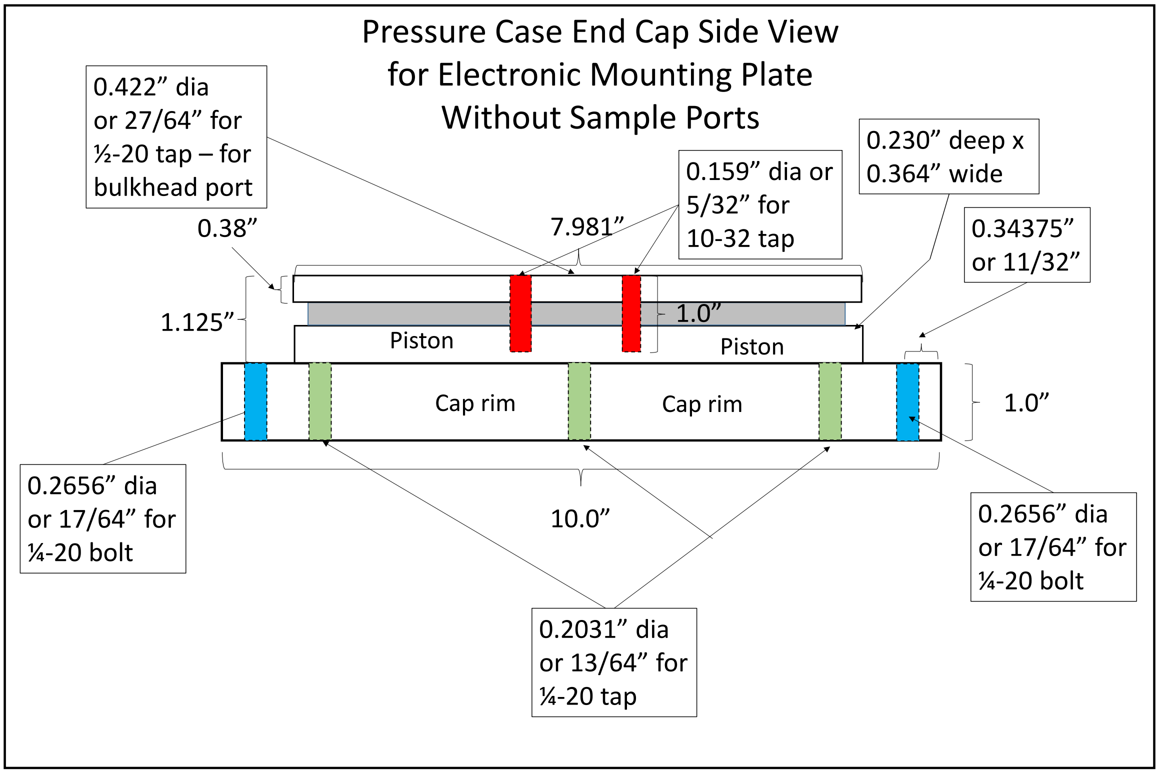

Pressure case housing electronics end cap without sample ports, side view.

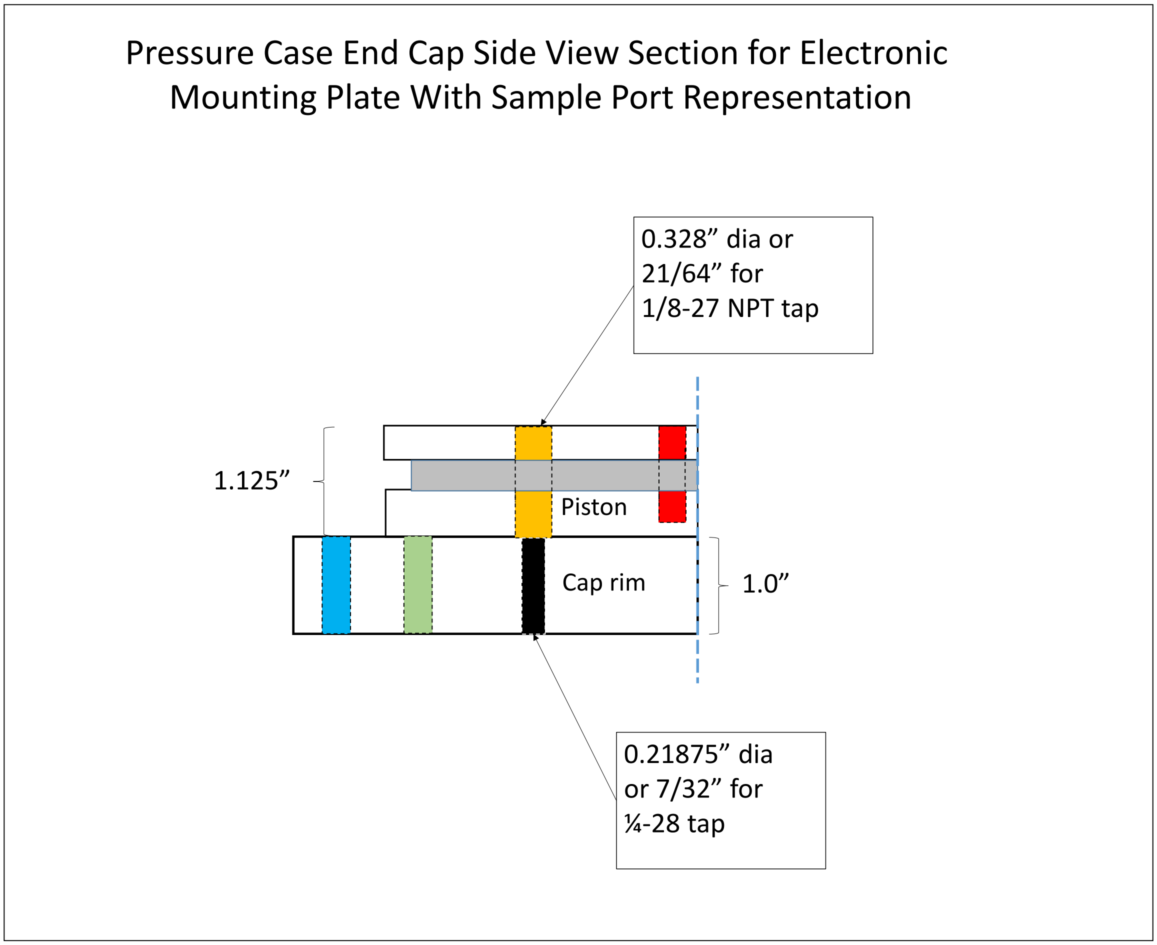

Pressure case housing electronics end cap showing the example of one sample port shown in black and yellow.

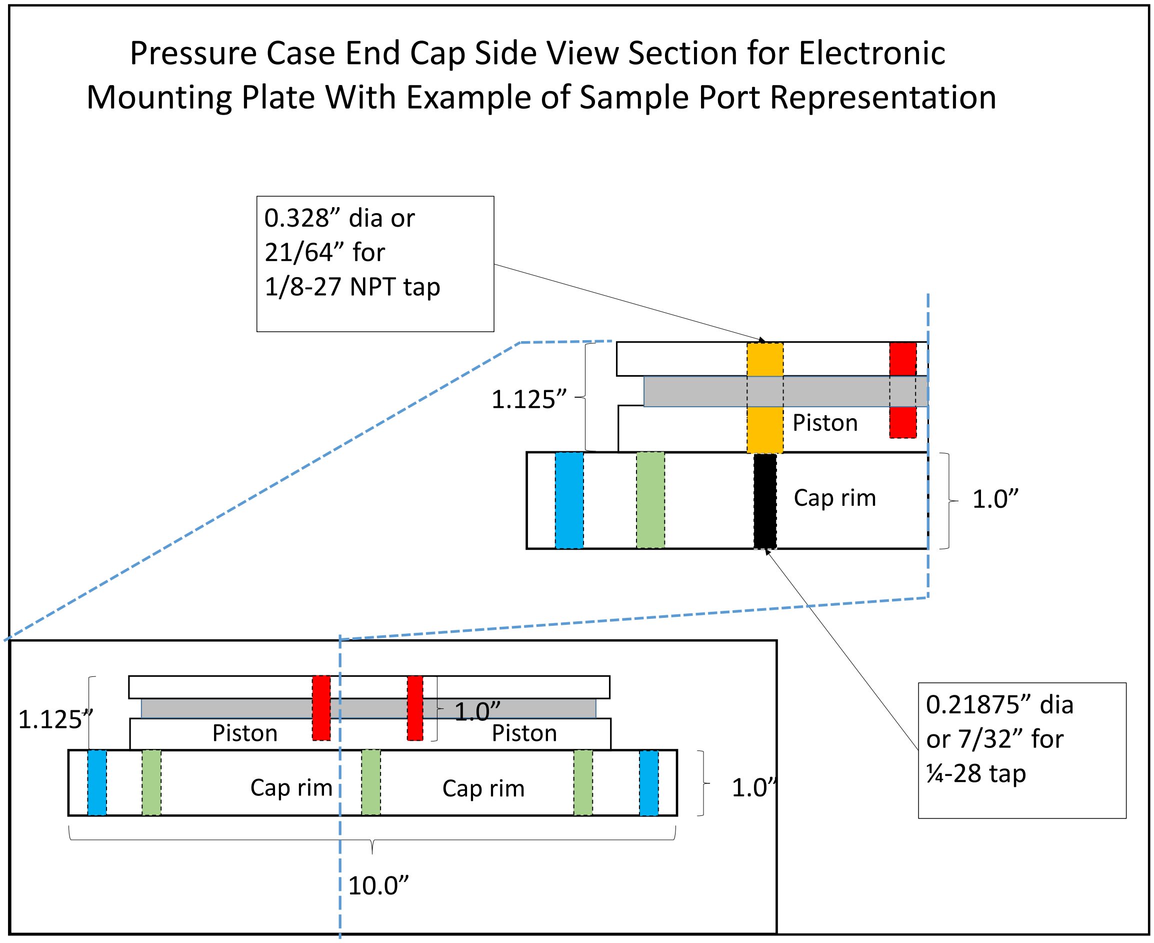

Pressure case electronics end cap with a close up of how the sample ports are drilled, side view.

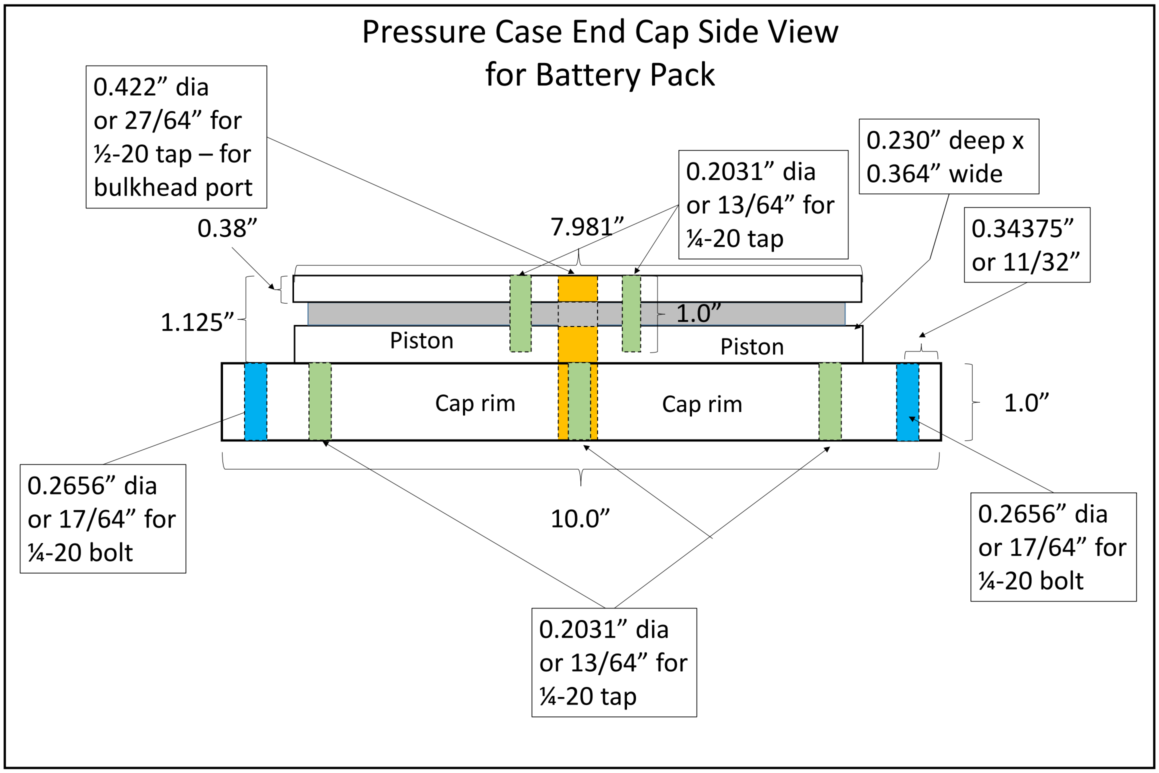

Pressure case end cap for the battery pack, side view.

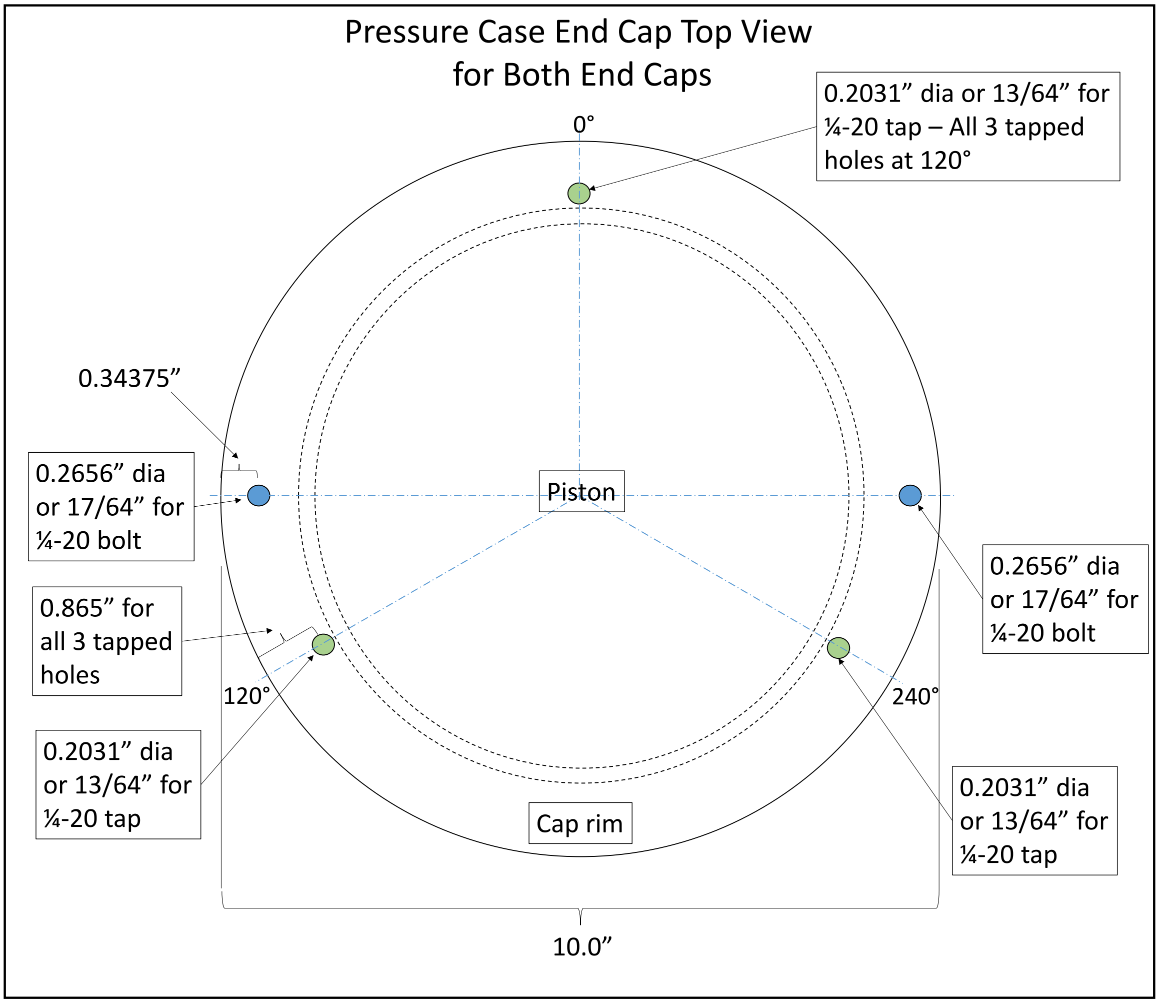

Pressure case end cap for the battery pack and the electronics, top view.

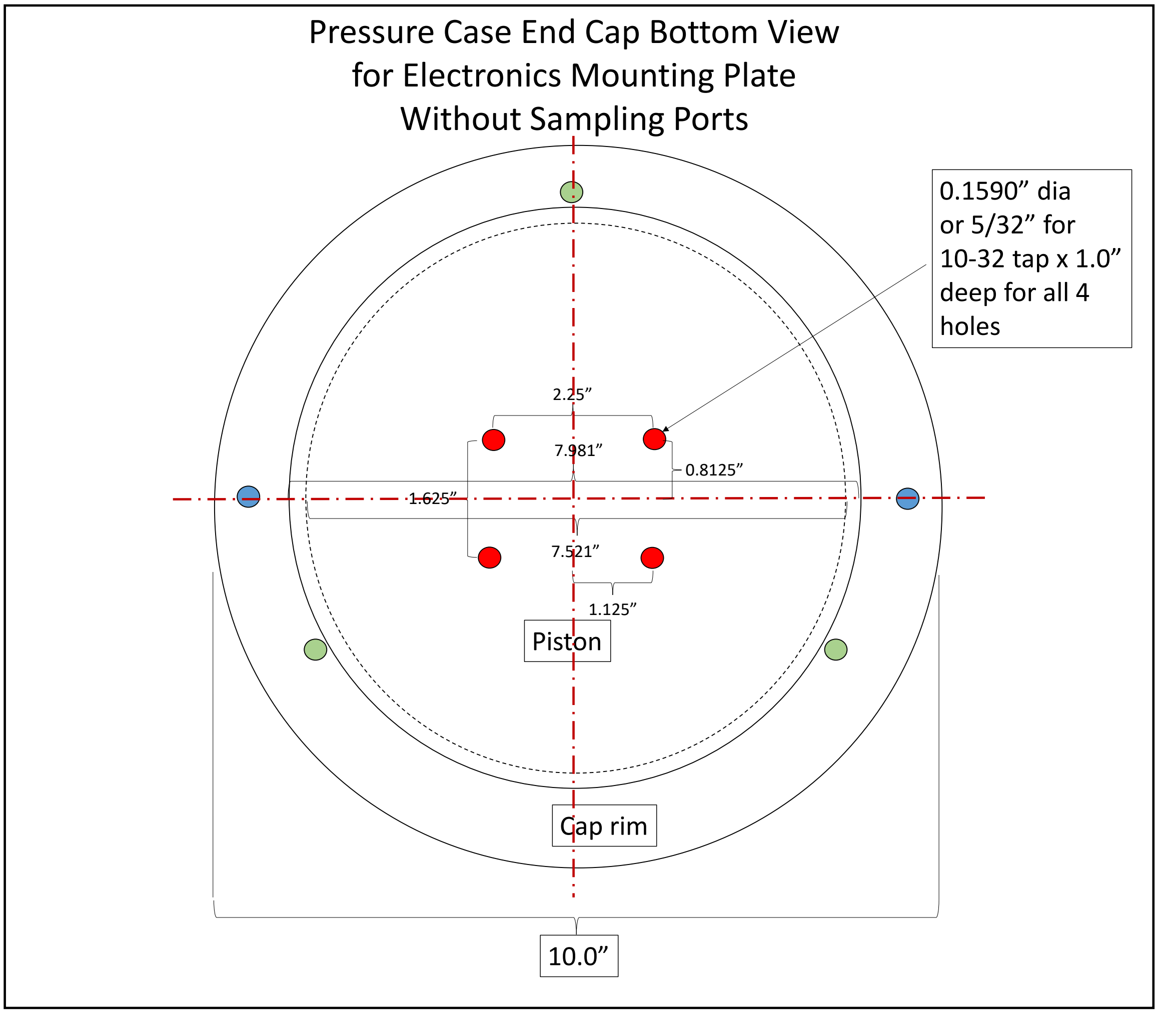

Pressure case end cap for the electronics mounting plate showing the location for the standoffs, bottom view.

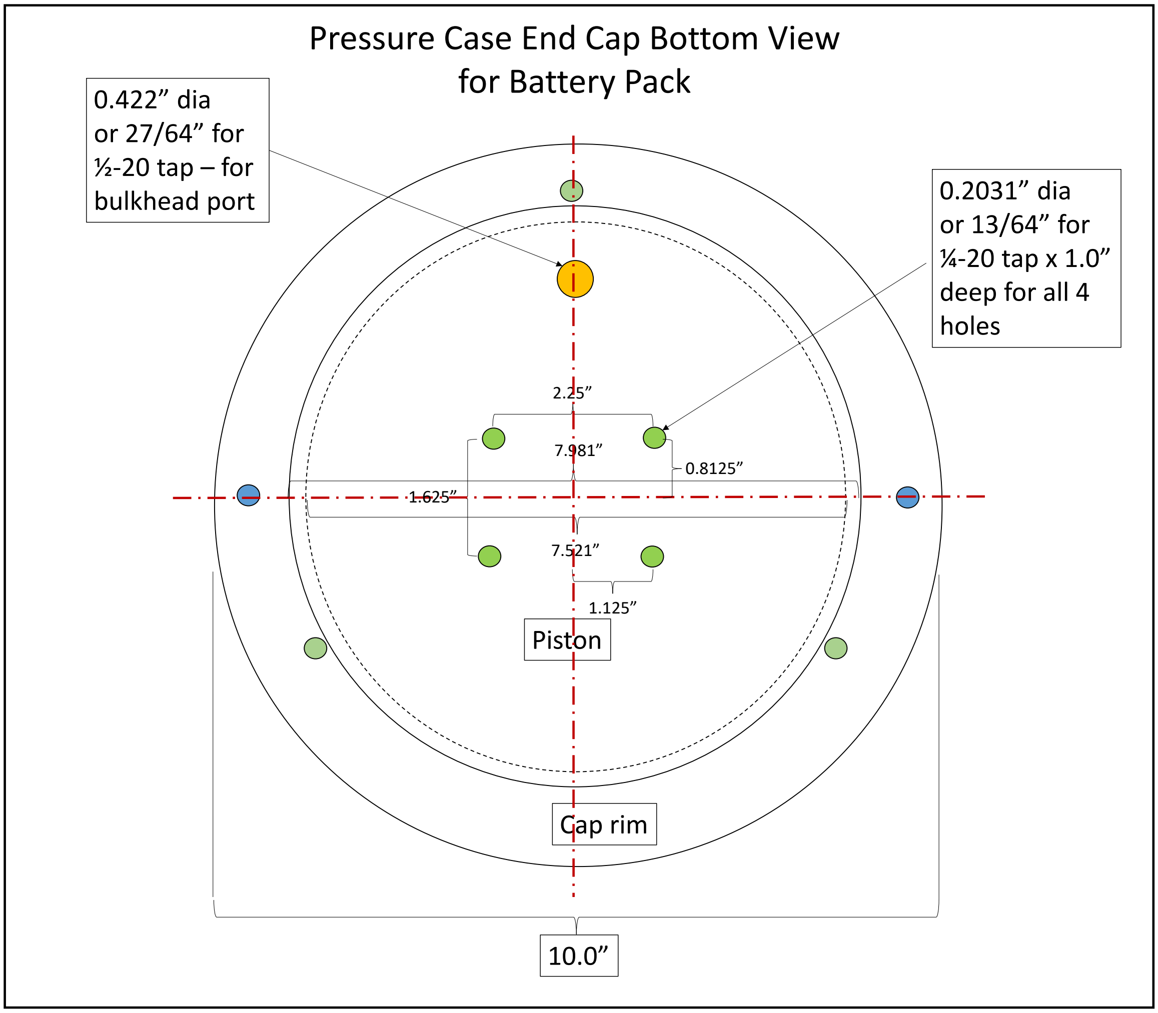

Pressure case end cap for the battery pack showing the location for the threaded rods and purge plug, bottom view.

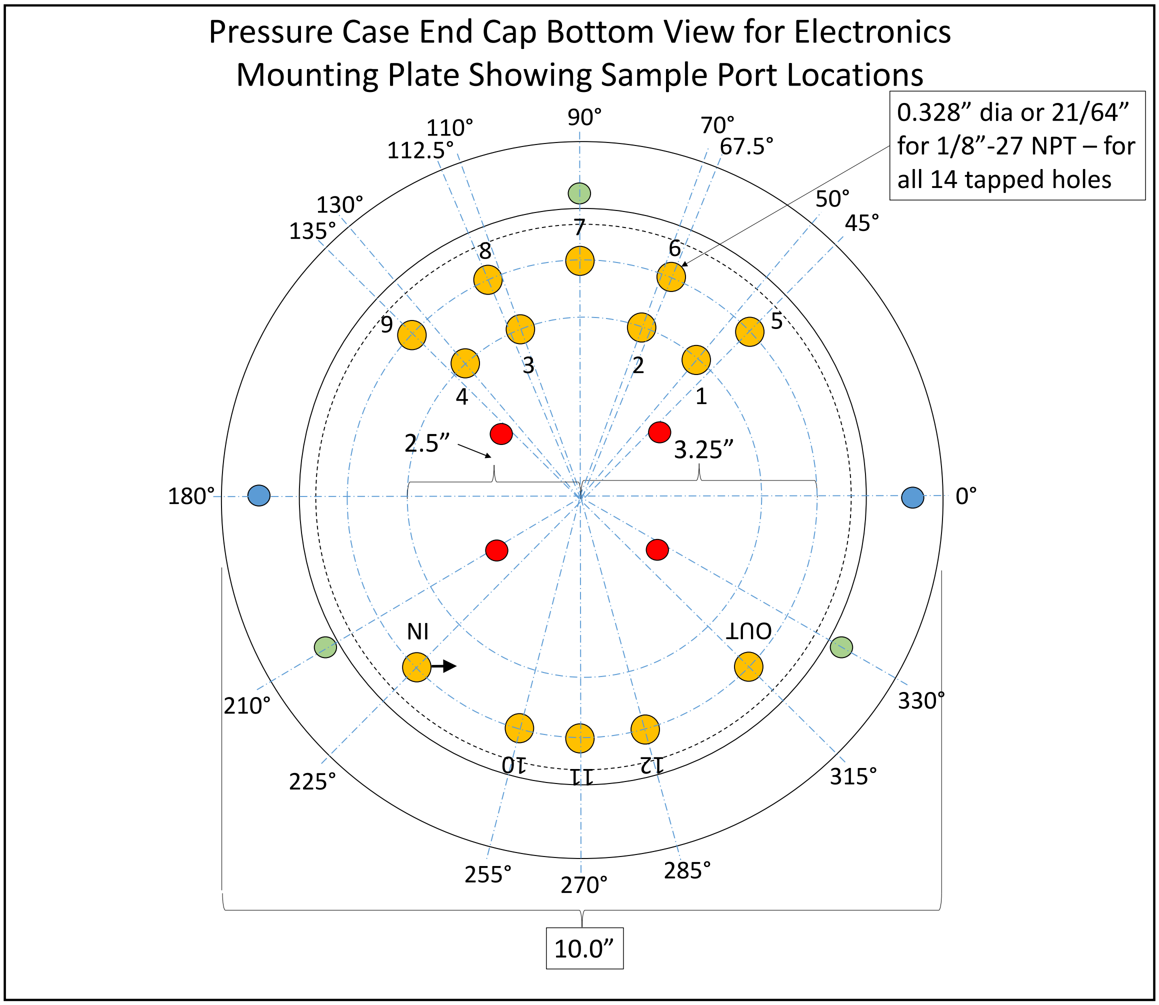

Pressure case end cap for the electronics mounting plate showing the location for the standoffs and sample ports, bottom view.

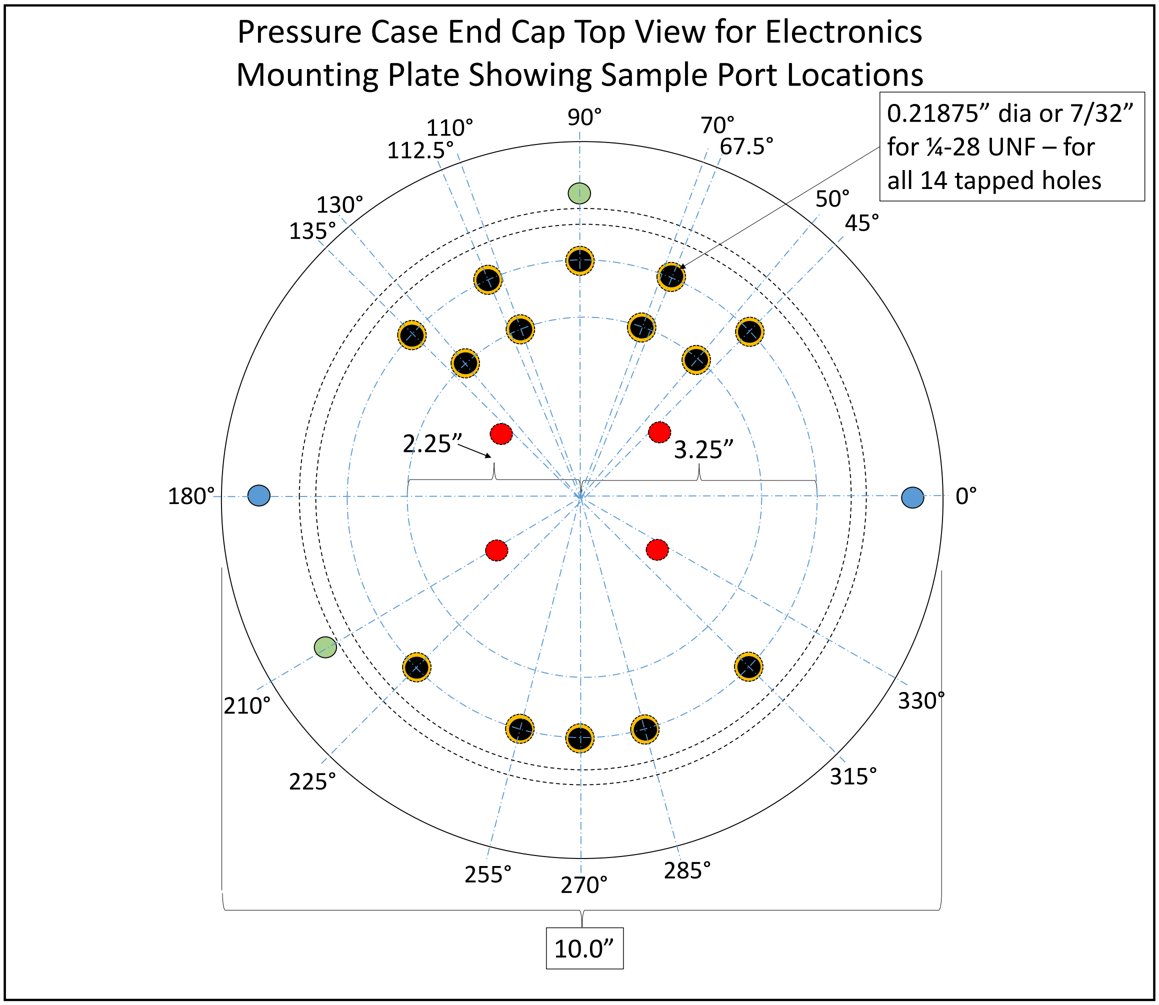

Pressure case end cap for the electronics mounting plate showing the location for the standoffs and sample ports, top view.

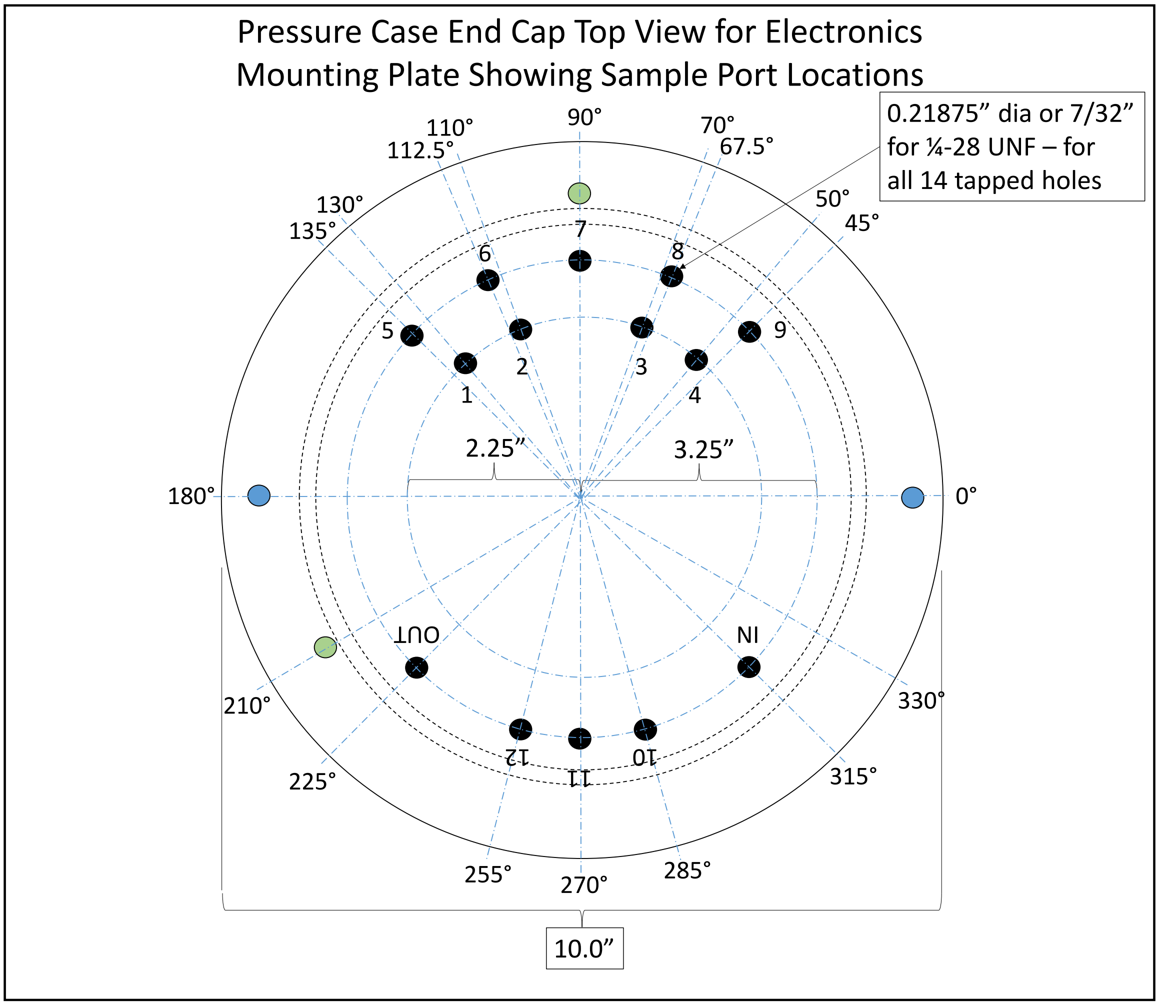

Pressure case end cap for the electronics mounting plate showing the location for the standoffs and sample ports, top view.

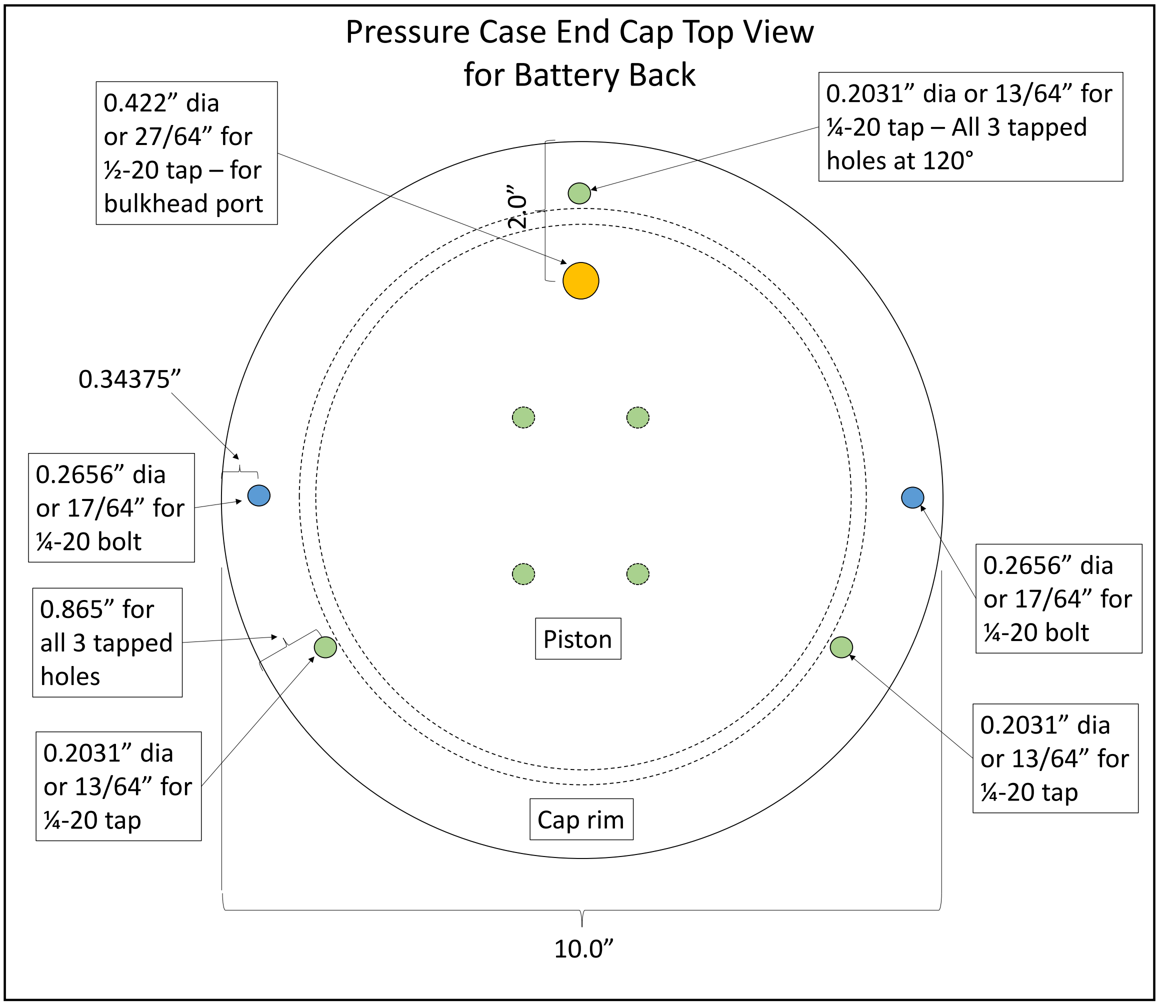

Pressure case end cap for the battery pack showing the location for the threaded rods and purge plug, top view.

Section 2: Acrylic discs for the electronics and battery pack

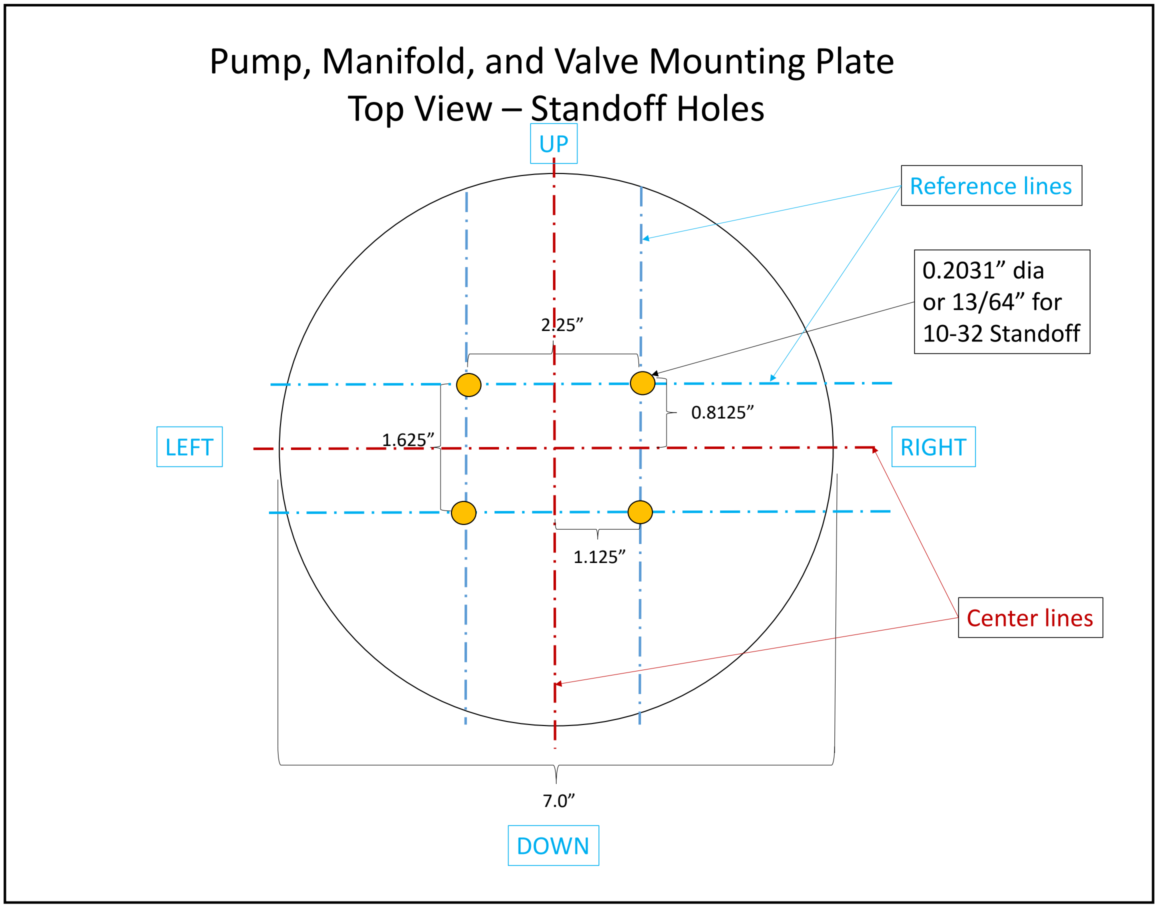

Acrylic disc for manifold/valve/pump plate showing reference lines for standoffs, top view.

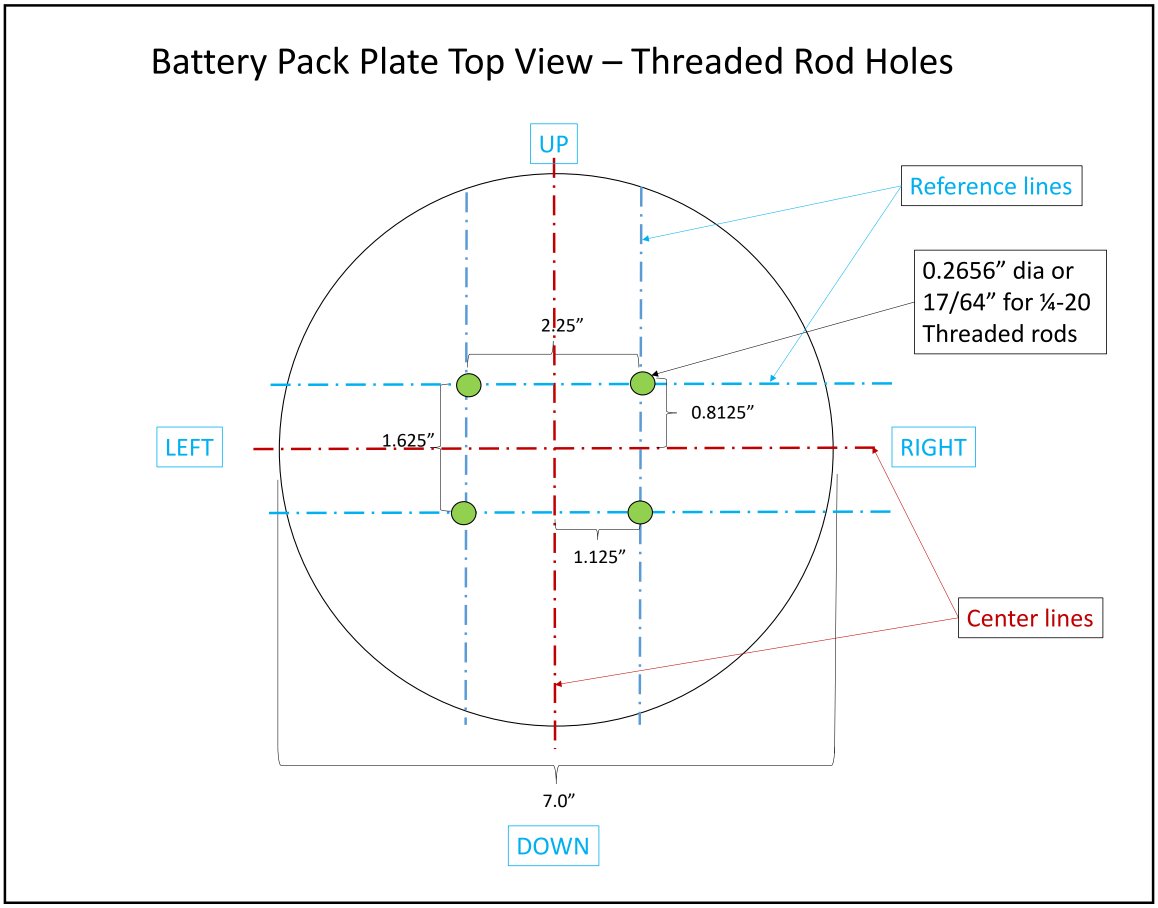

Acrylic disc for battery pack showing reference lines for threaded rods, top view.

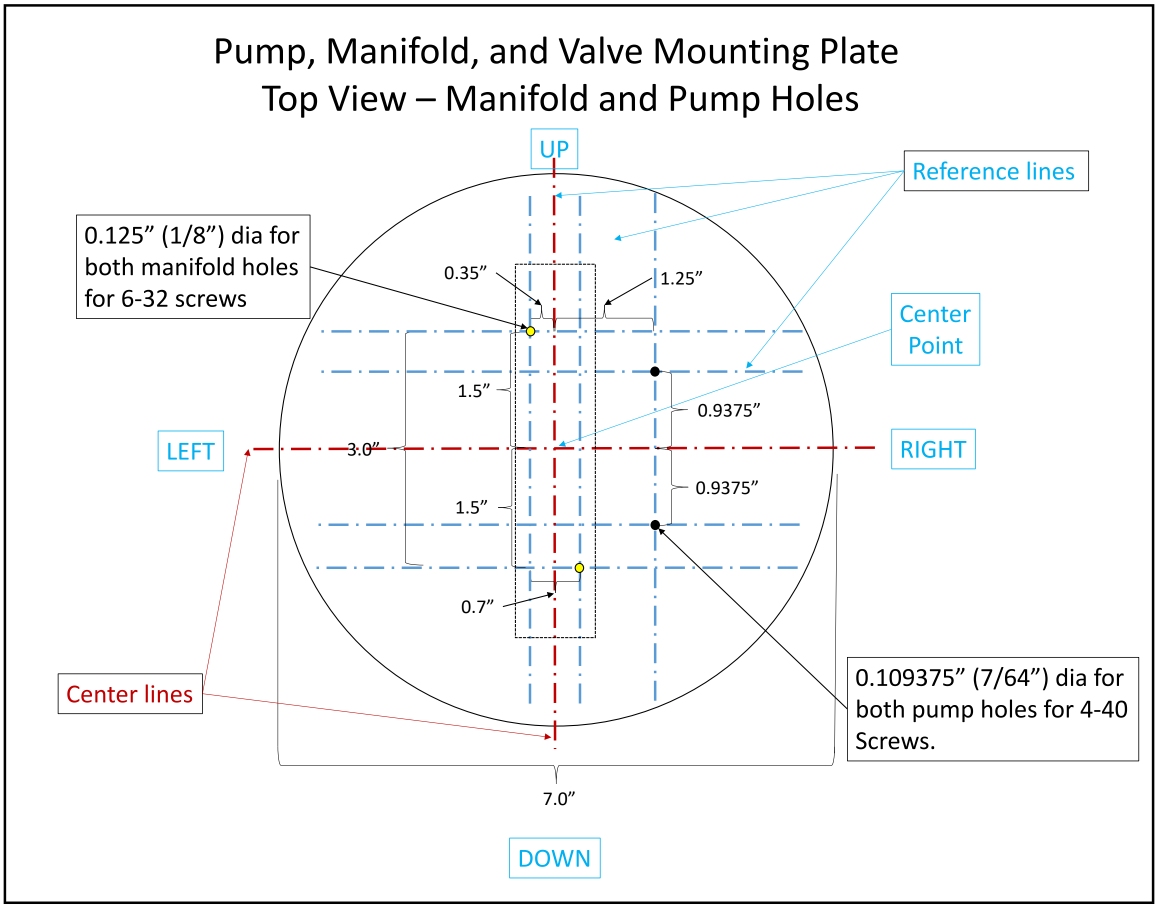

Acrylic disc for manifold/valve/pump plate showing reference lines for the peristaltic pump and manifold, top view.

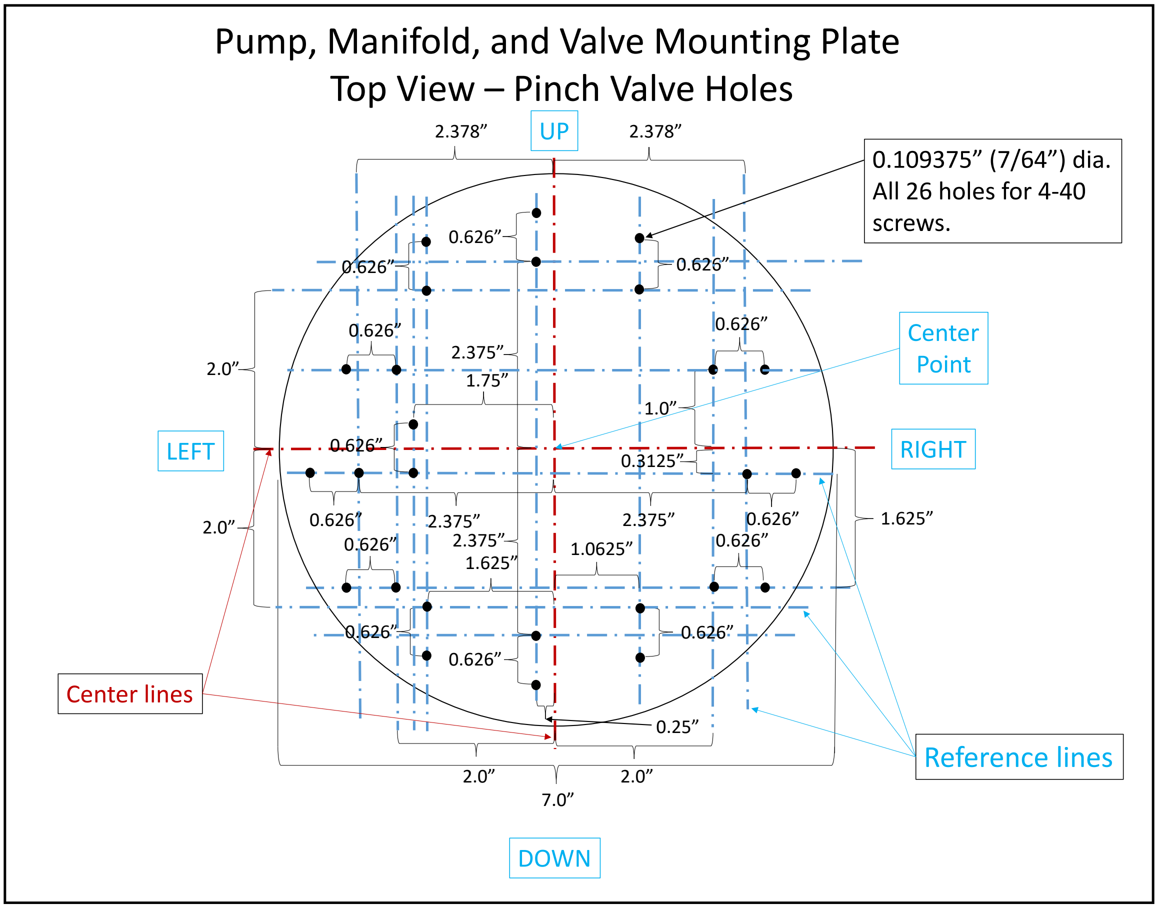

Acrylic disc for manifold/valve/pump plate showing reference lines for the 13 pinch valves, top view.

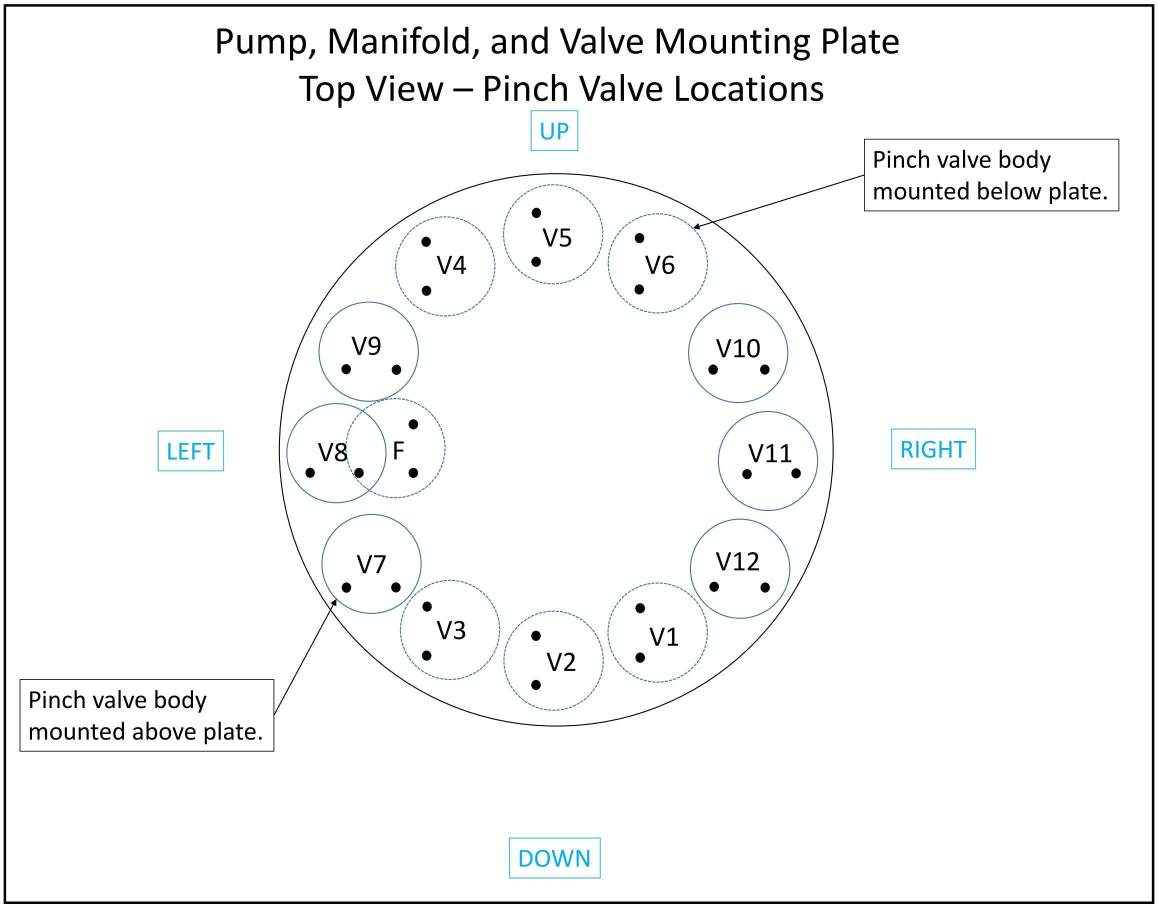

Acrylic disc for manifold/valve/pump plate showing the orientation of the 13 pinch valves, top view.

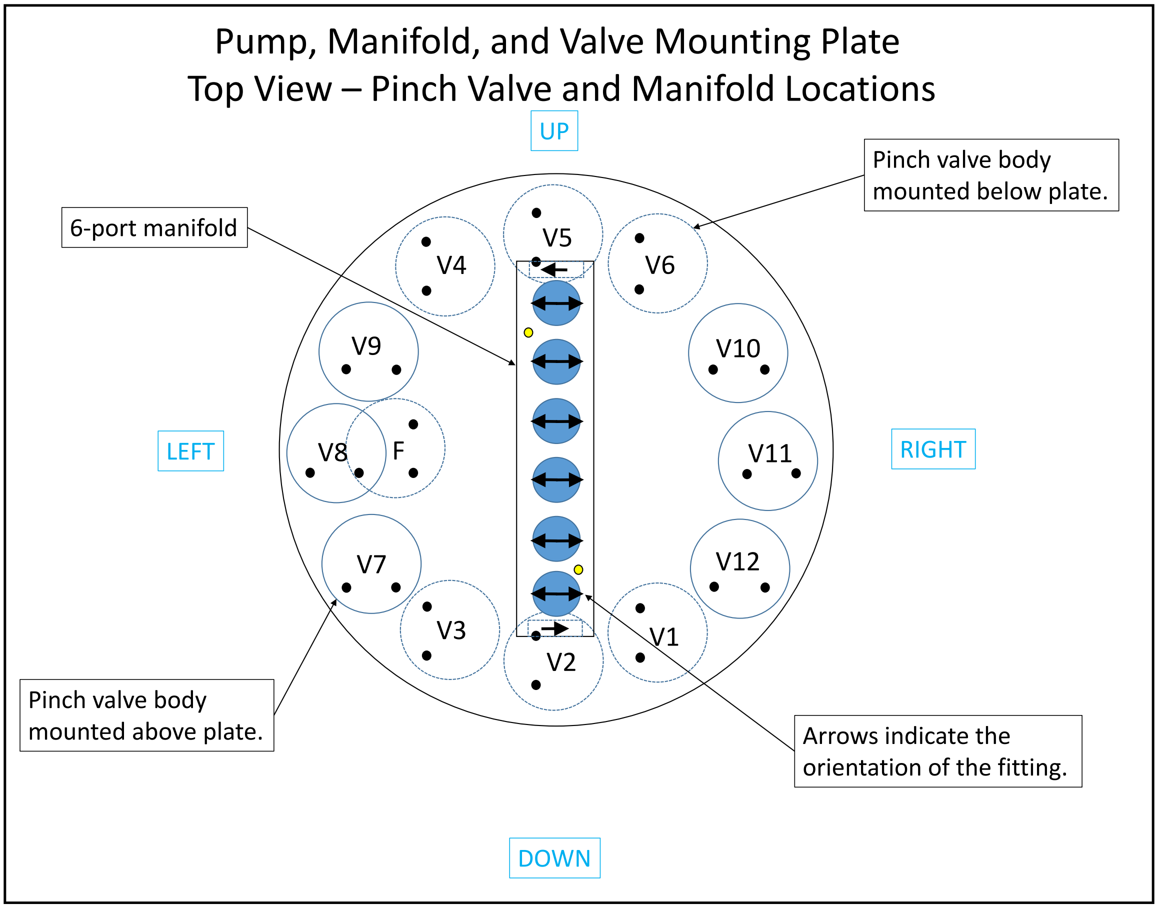

Acrylic disc for manifold/valve/pump plate showing the orientation of the 13 pinch valves and the manifold, top view.

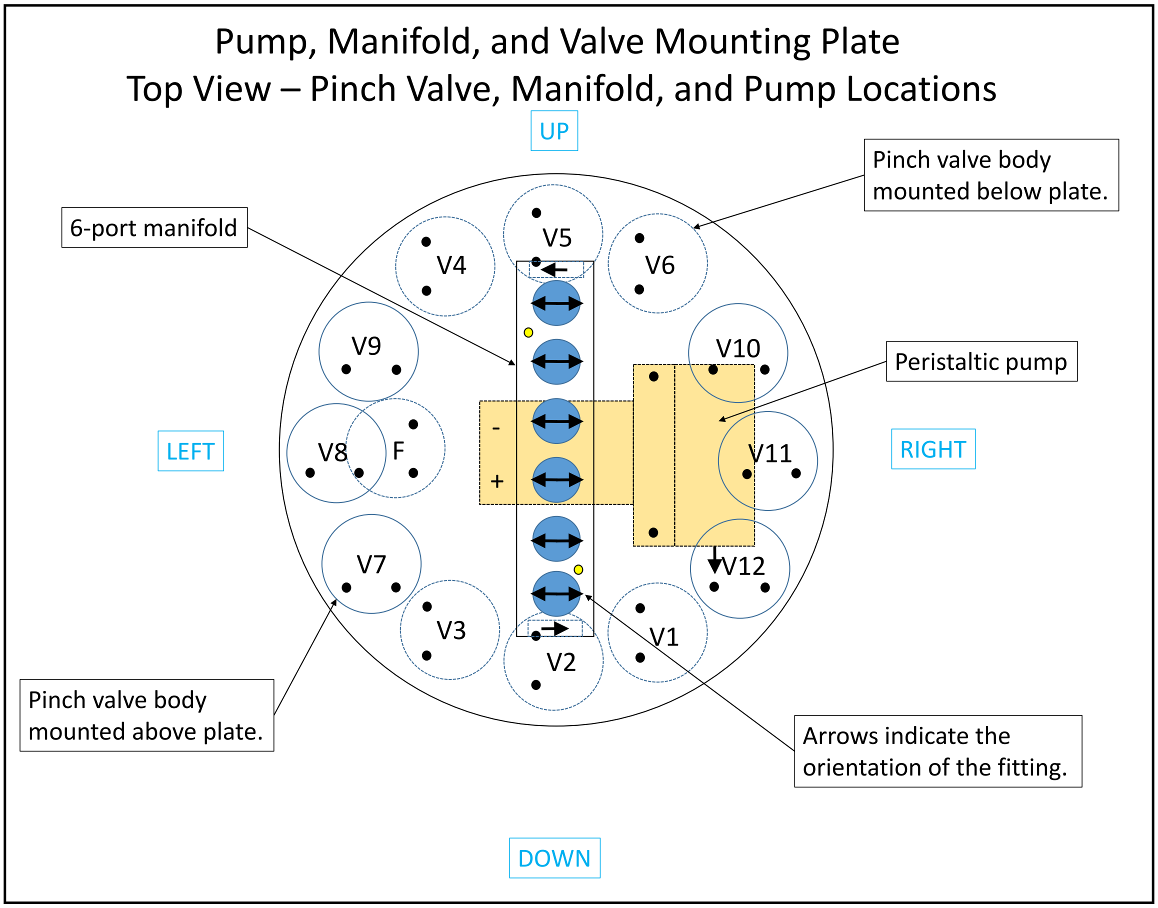

Acrylic disc for manifold/valve/pump plate showing the orientation of the 13 pinch valves, manifold and the peristaltic pump, top view.

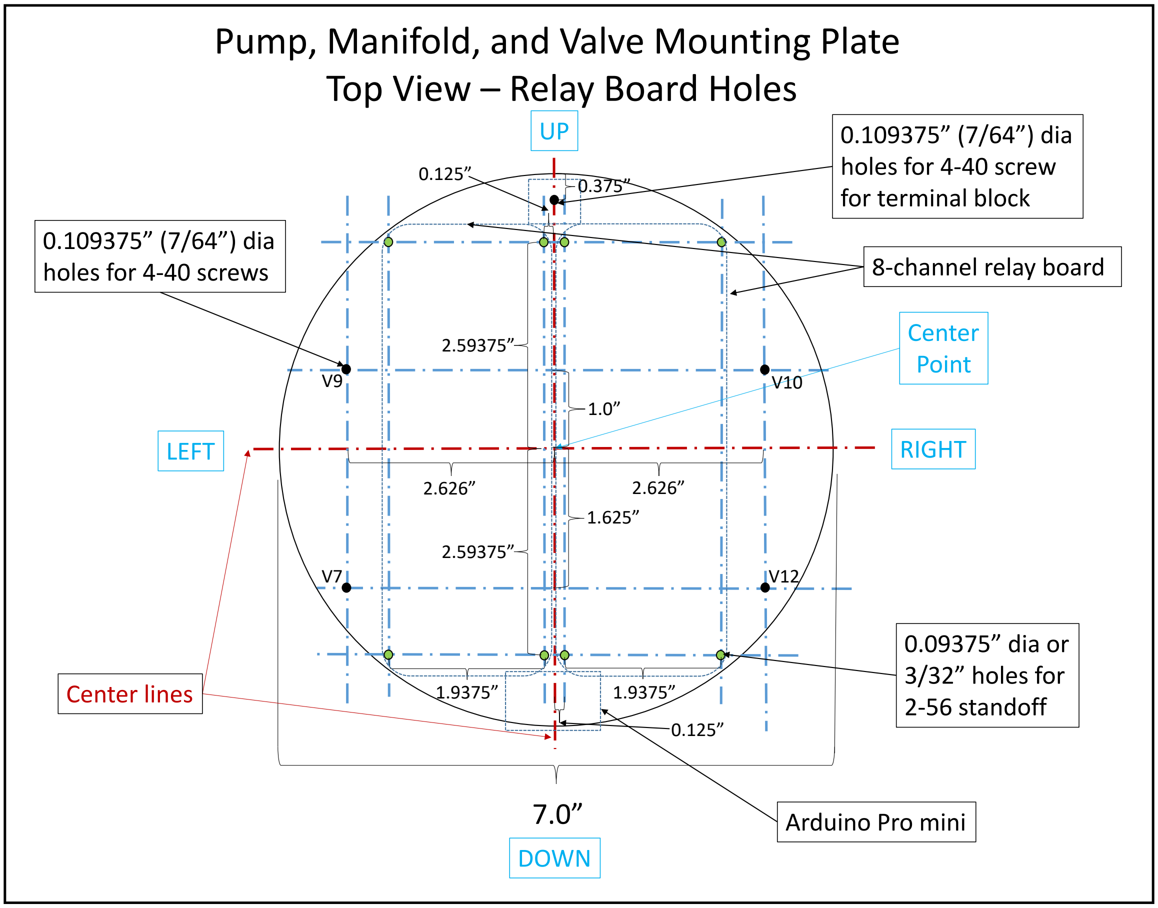

Acrylic disc for the electronics mounting plate showing the reference lines for the relays, terminal block, and mounting holes to attach to the manifold/valve/pump plate, top view.

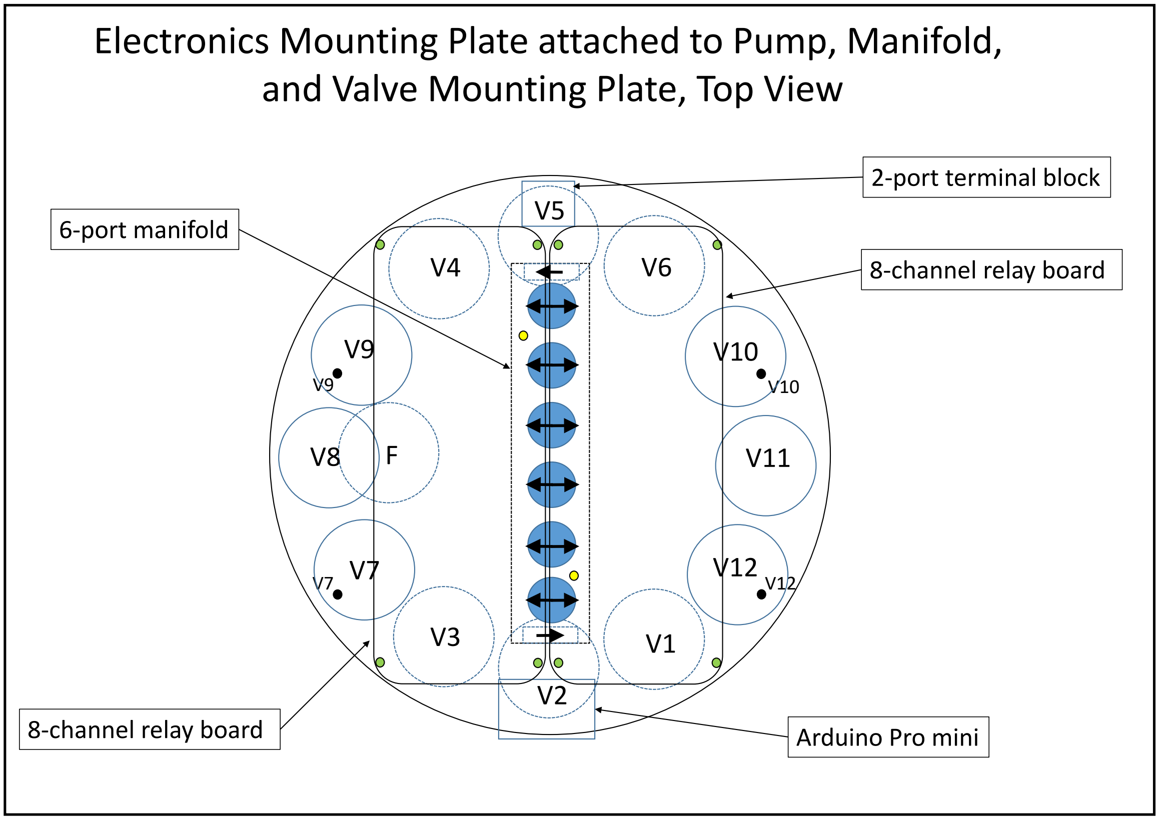

Illustration of the manifold/valve/pump plate with the electronics plate mounted on top with the outlines of all components, top view.

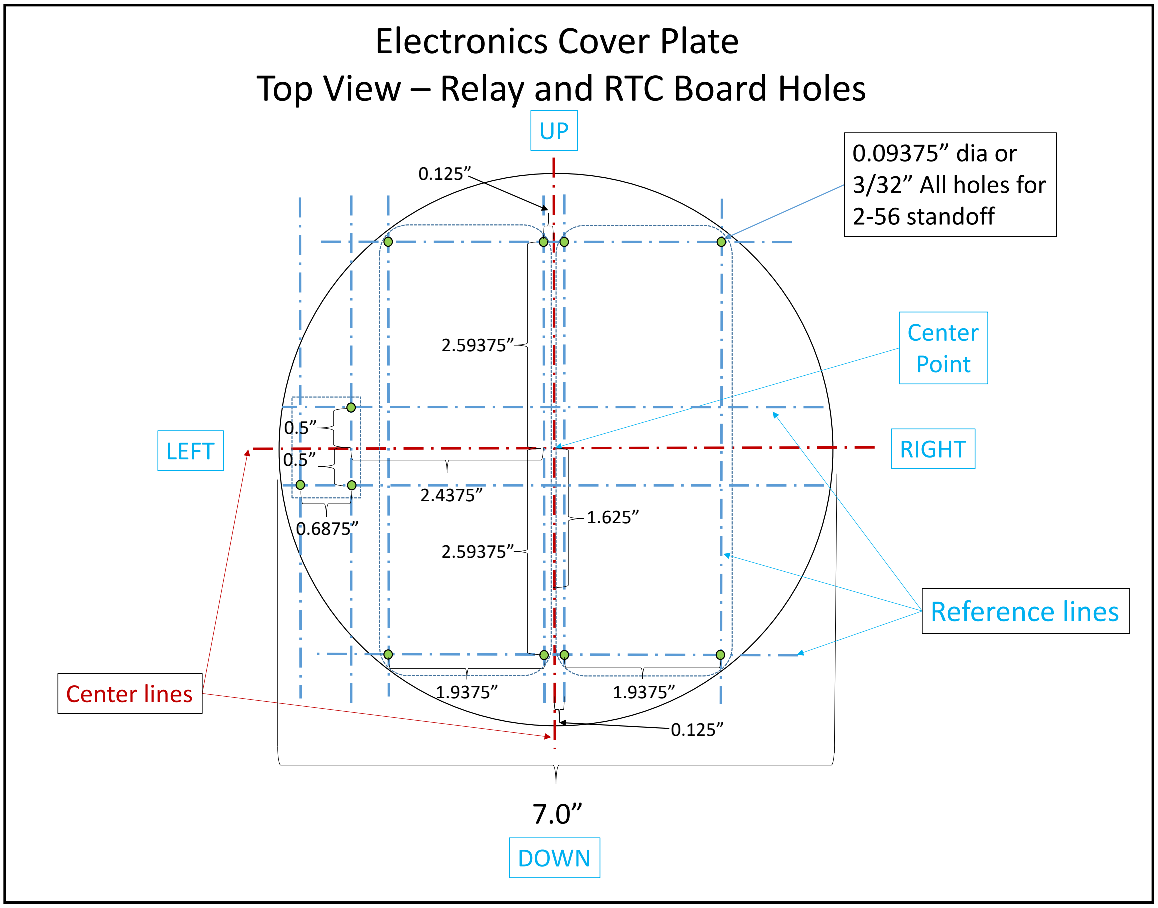

Acrylic disc for the electronic mounting plate showing reference lines for relays and real-time clock (RTC), top view.

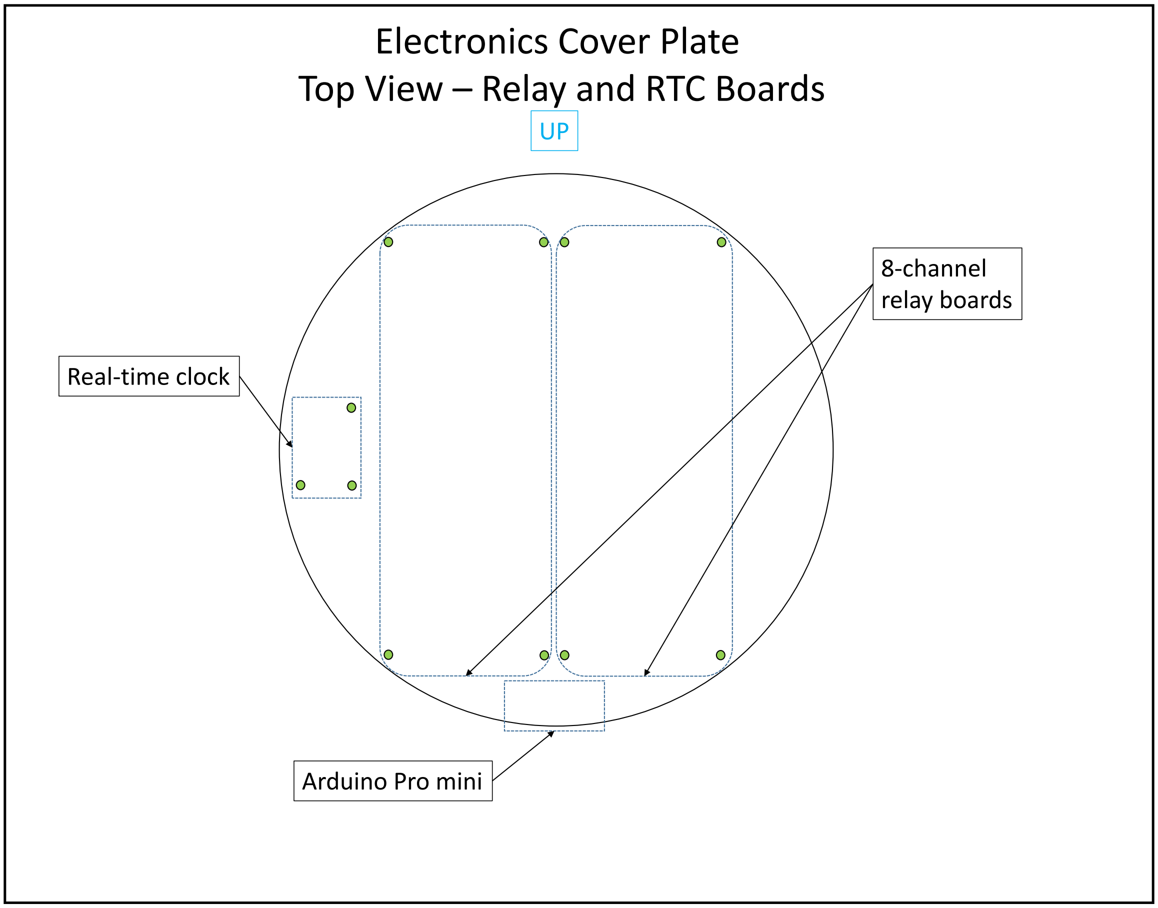

Acrylic disc for the electronic mounting plate showing the orientation of the relay board, real-time clock (RTC), and Arduino controller, top view.

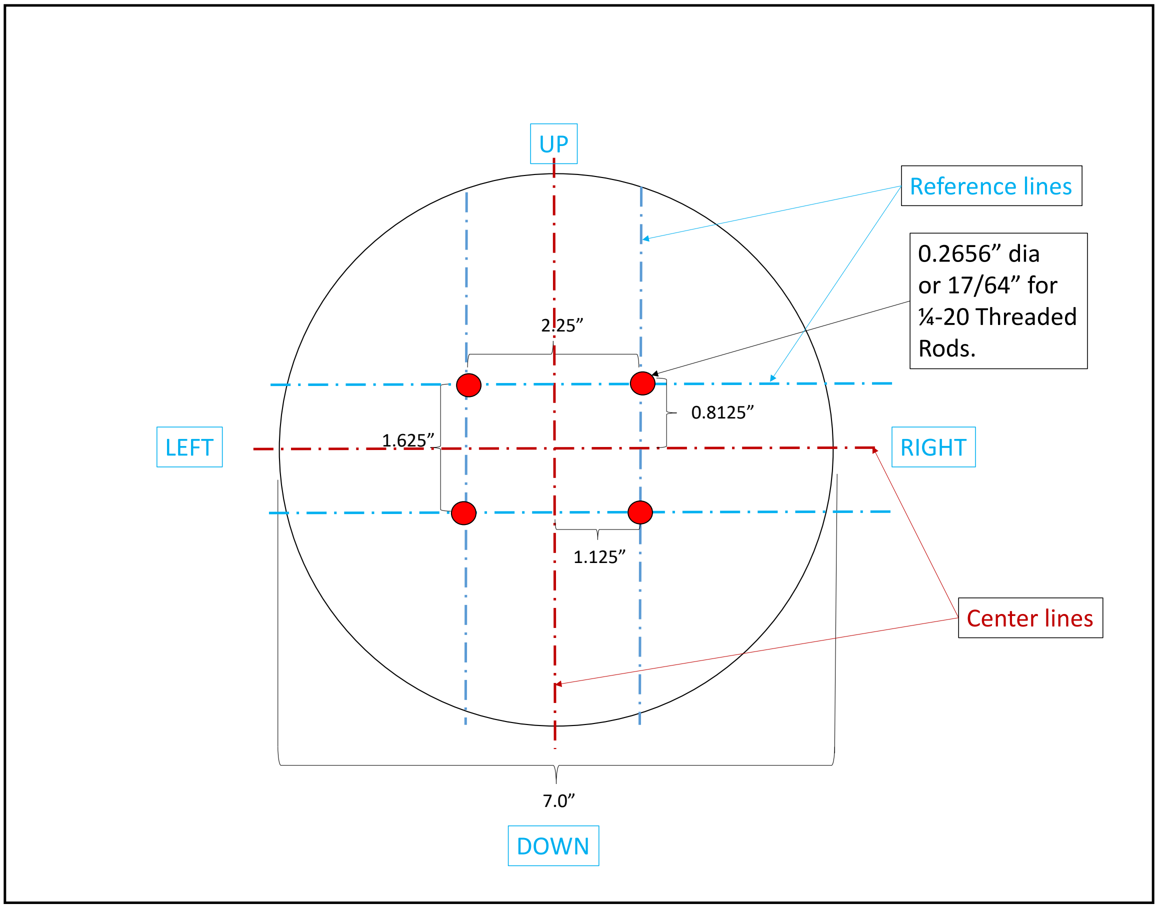

Acrylic disc for the battery pack mounting plate showing reference lines for the threaded rods, top view.

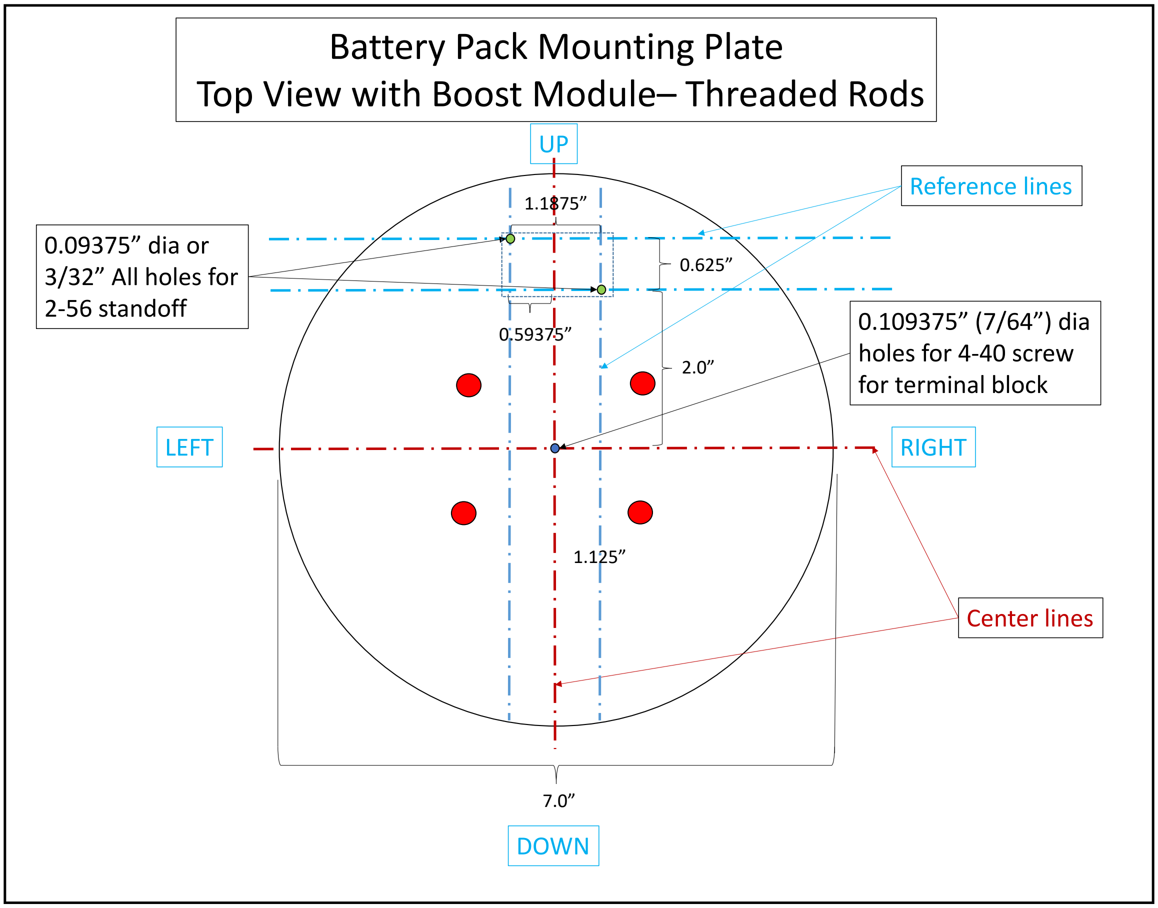

Acrylic disc for the battery pack mounting plate showing reference lines for the threaded DC to DC boost converter module XL6009.

Pressure Case End Cap Bottom View With Manifold, Pump, and Pinch Valve Plate

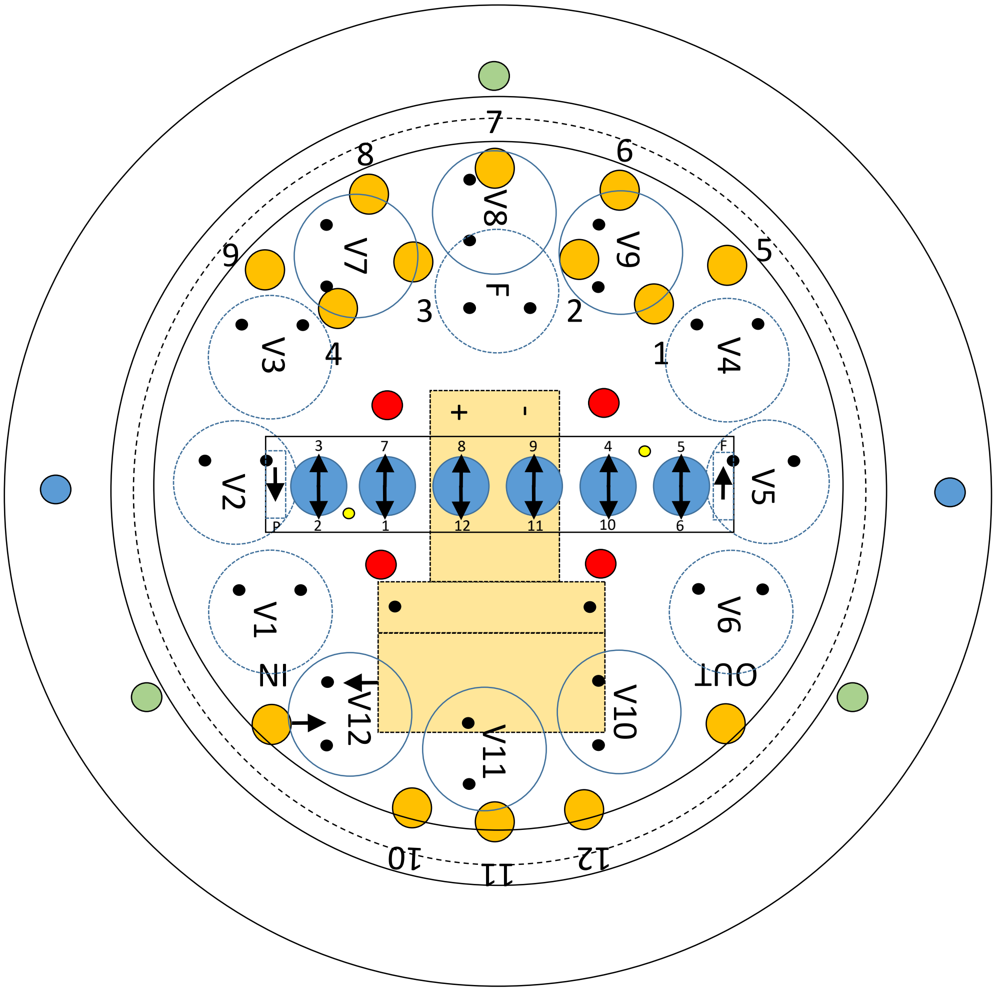

Illustration showing the orientation of the manifold/valve/pump plate mounted on to the pressure case housing end cap.

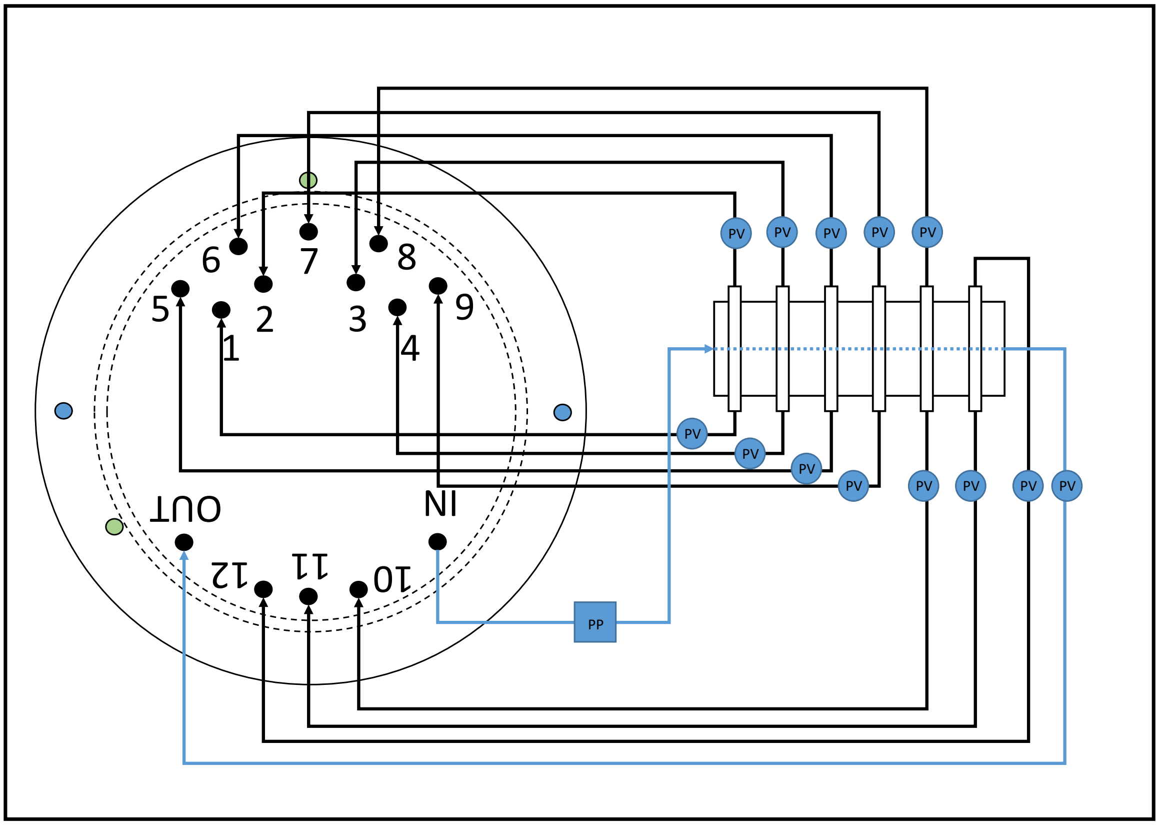

Illustration the pumping direction and connections between the peristaltic pump (PP), pinch valves (PV), manifold, and the pressure case housing end cap.

Section 3: AutoSampler Wiring Schematics

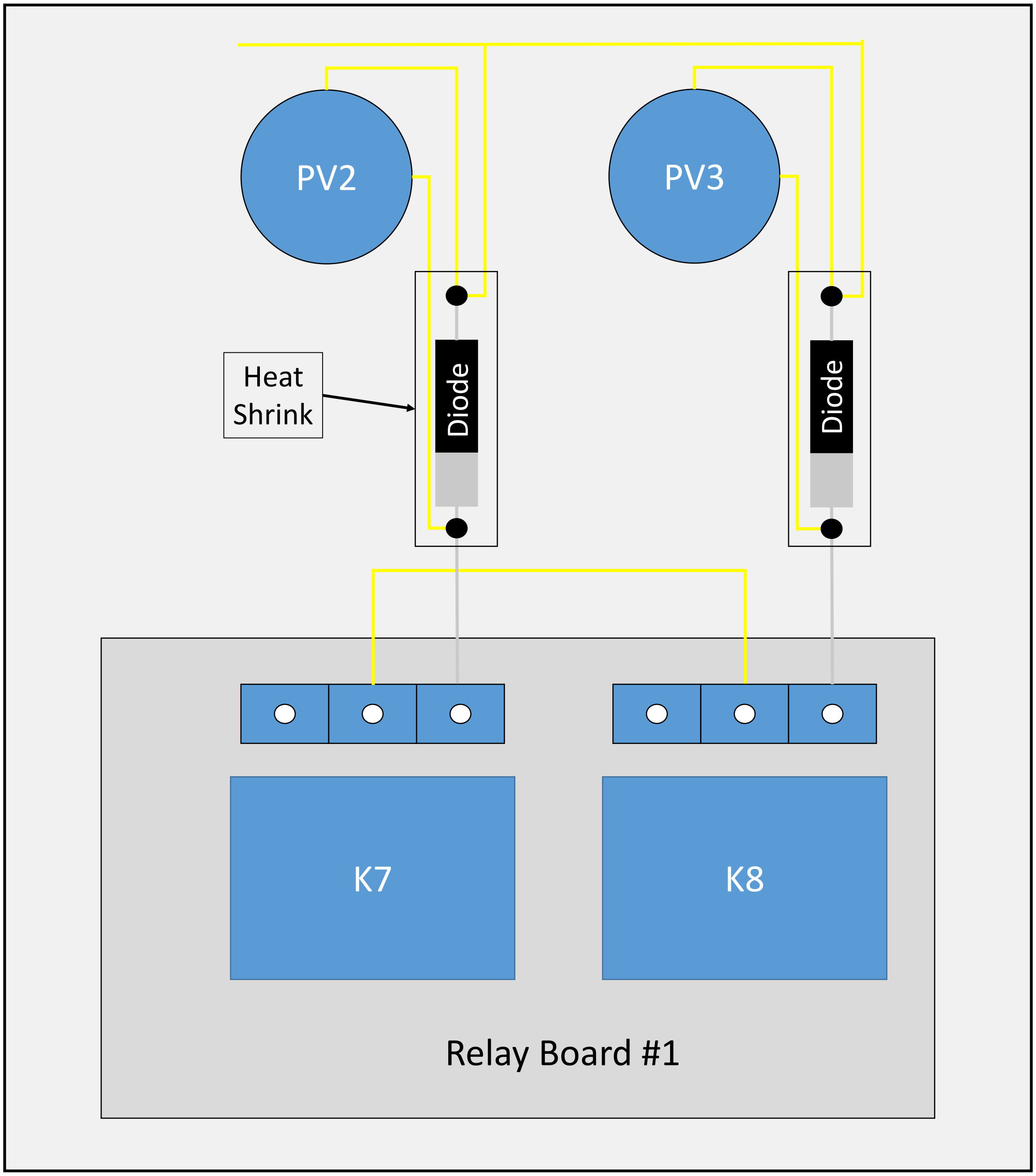

Close up illustration of how 2 of the 13 pinch valve are connected to the relay board using diodes.

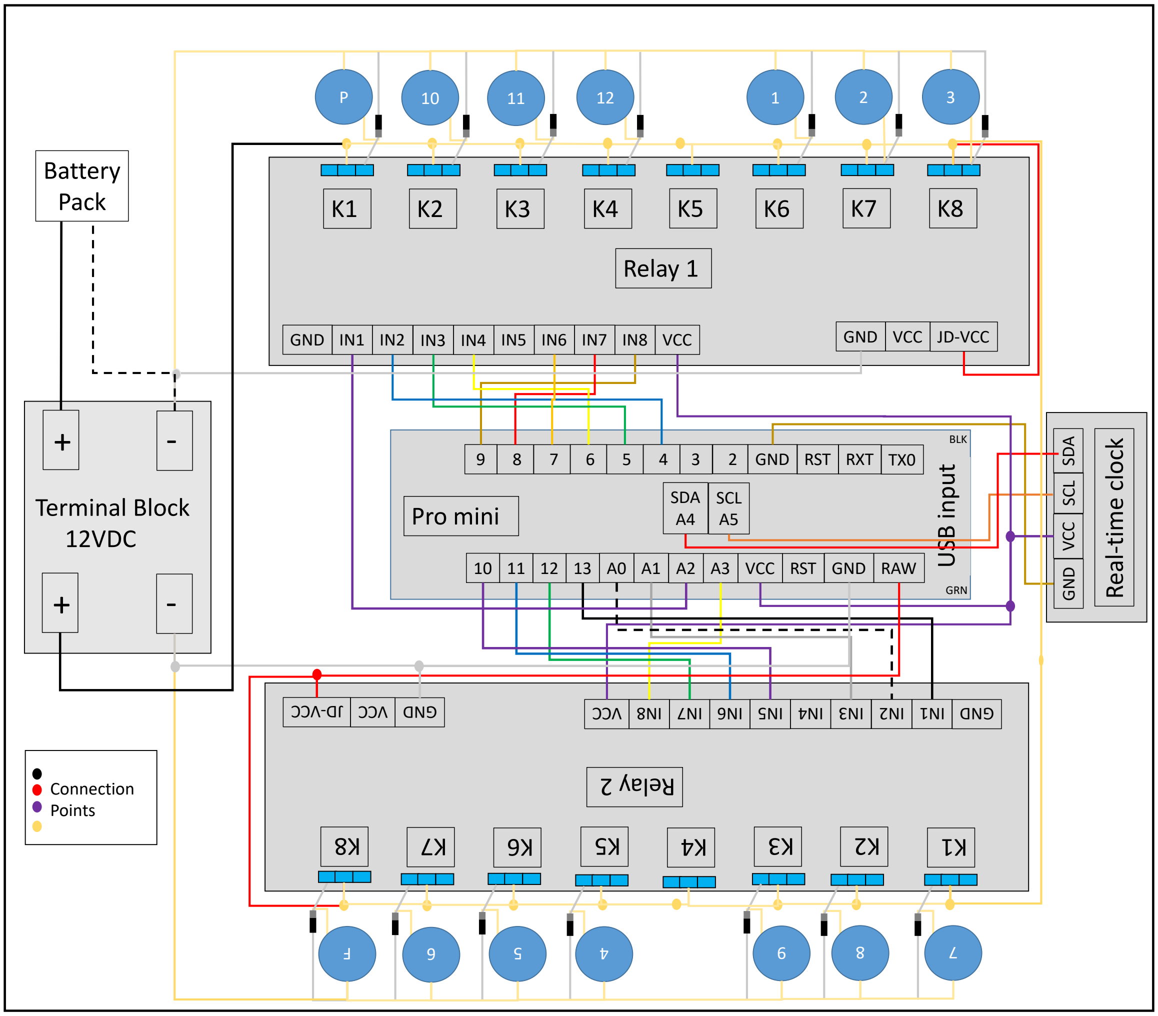

AutoSampler complete wiring diagram

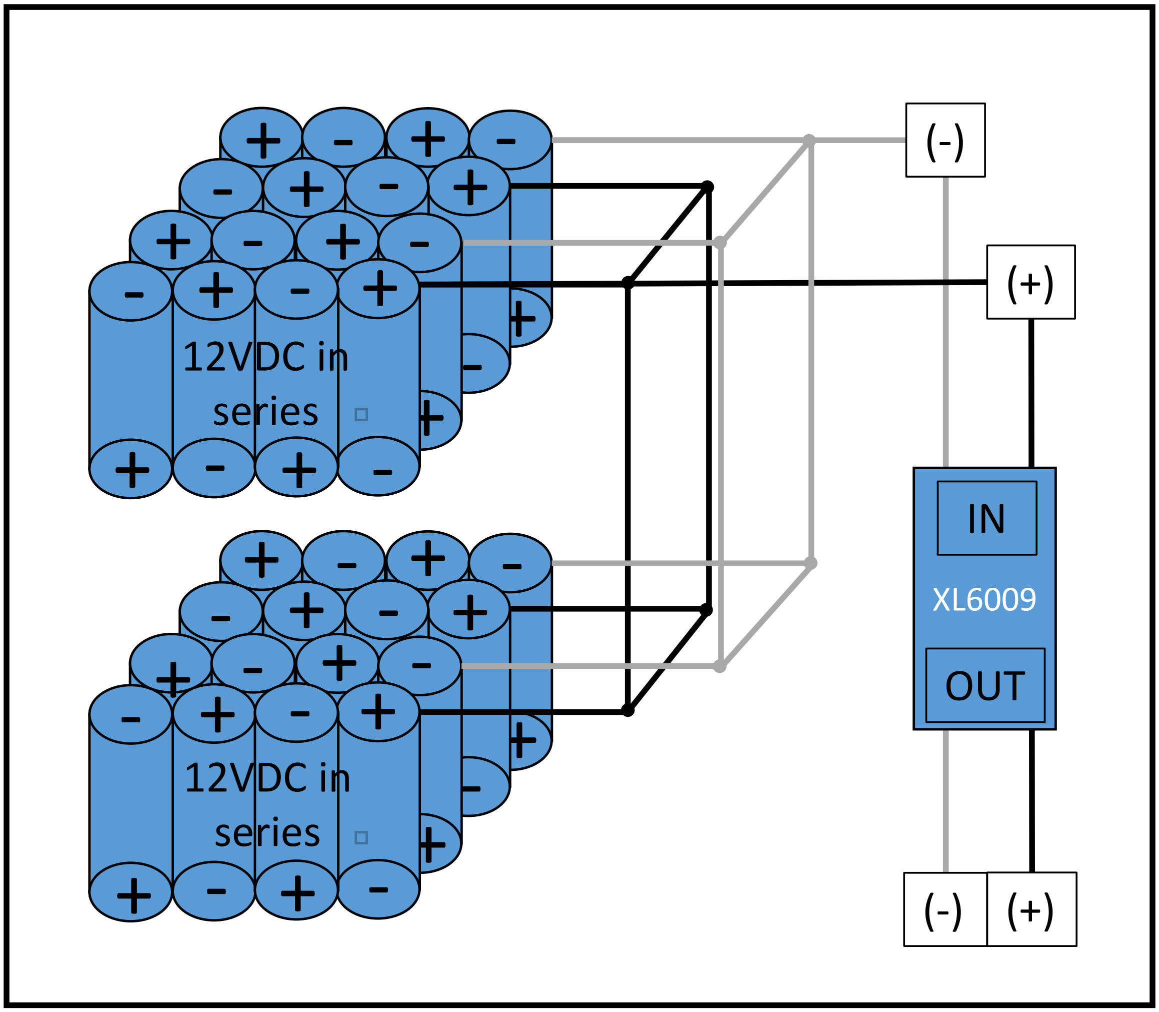

AutoSampler battery pack wiring diagram.

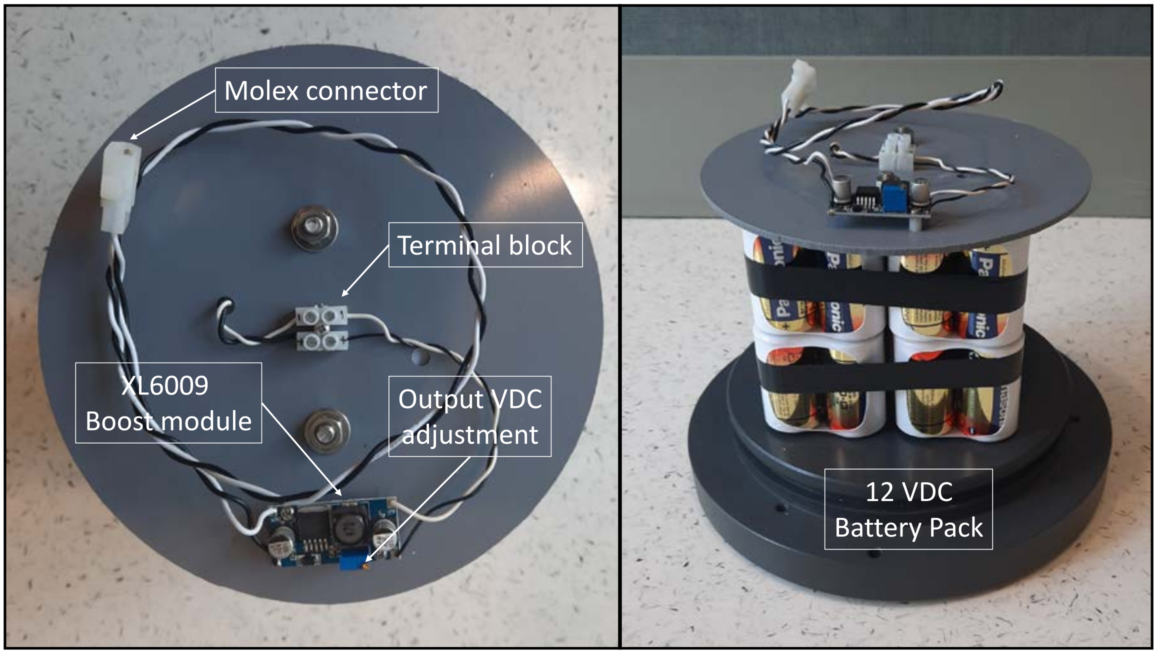

AutoSampler battery pack disc assembly, top view on the left and side view on the right.

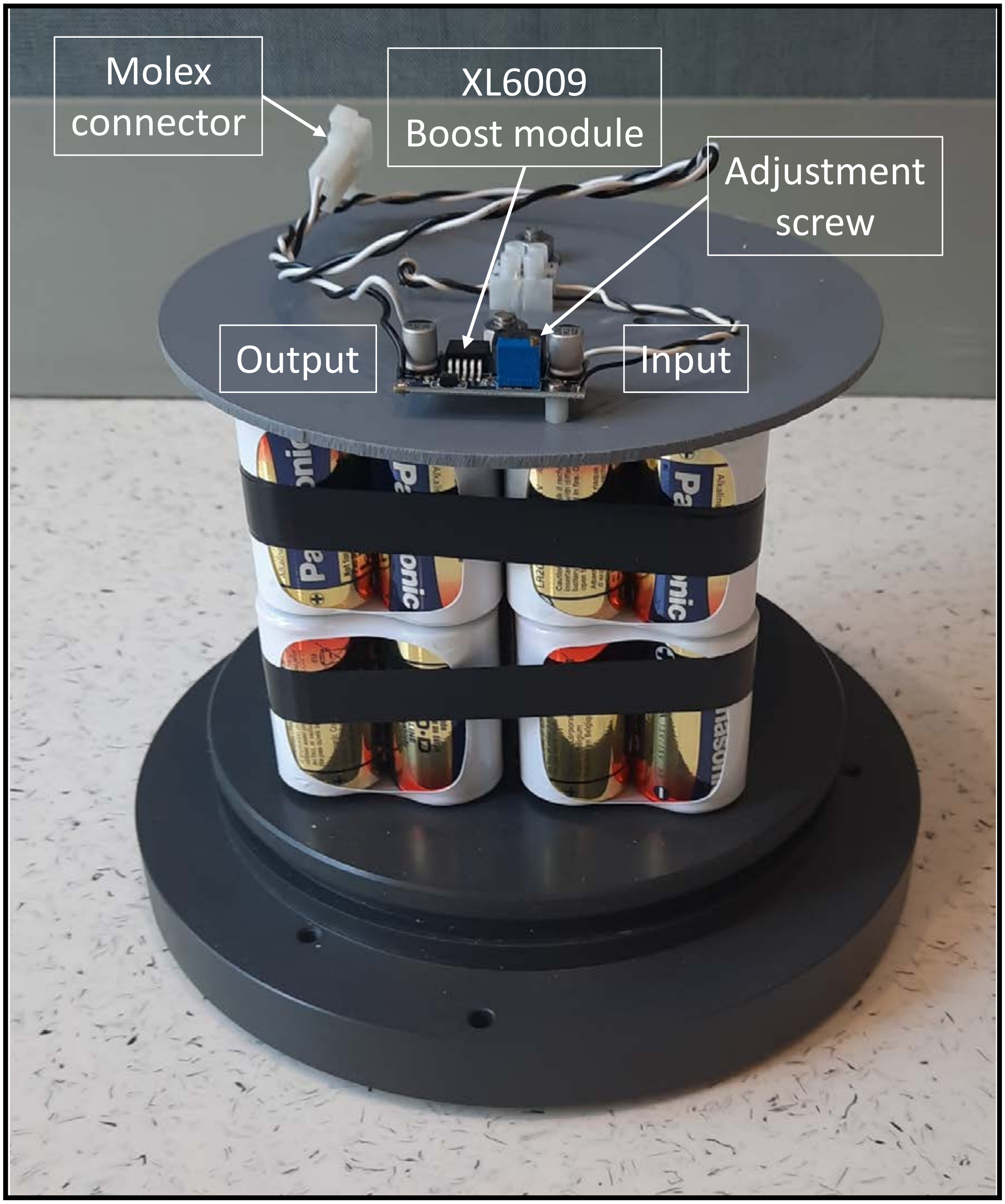

AutoSampler battery pack disc assembly with XL6009 boost module.
