# Supplementary material for: Autonomous submersible multiport water sampler
Source: HardwareX. 2021 Apr 22;9:e00197. doi: 10.1016/j.ohx.2021.e00197 (PMC9041238; doi:10.1016/j.ohx.2021.e00197)
Supplement: Supplementary data 5 [file mmc5.pdf]

Stanford Underwater Laboratory

MG1-V2

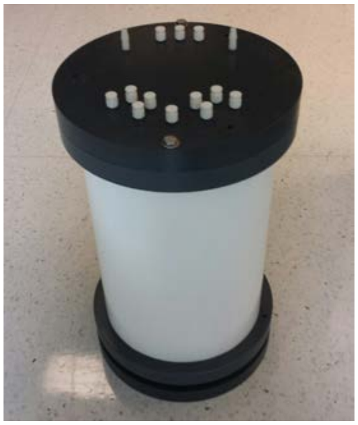

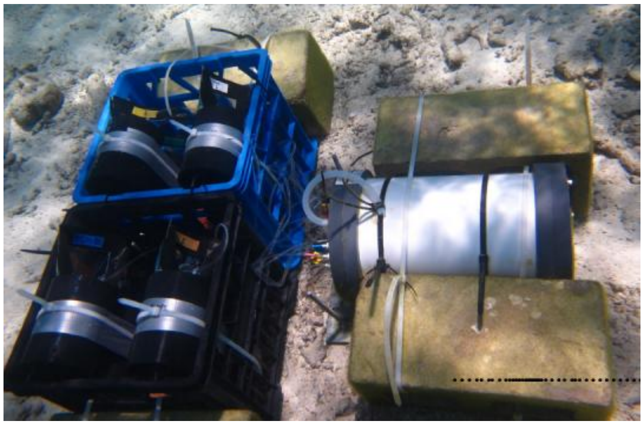

Autonomous Multiport Water Sampler (AutoSampler) User Manual

David A. Mucciarone, Hans B. DeJong, and Robert B. Dunbar (Stanford University), Yui Takeshita (Monterey Bay Aquarium Institute), Rebecca Albright (California Academy of Science), Keaton Mertz (Monterey Bay Aquarium Institute)

The Stanford Underwater Laboratory Multiport Water Sampler (AutoSampler), uses a peristaltic pump and a series of 13 solenoid valves to permit sampling up to 12 samples from a single source. The system is controlled by an Arduino Pro Mini interface, two 8-channel relay boards, and a real-time clock. The software is designed to delay deployment and can be programmed for up to 12 sampling cycles either in discrete mode or in continuous mode. Power is supplied by 12 VDC alkaline D-cell batteries. The battery compartment is designed to hold up to four 8-pack D-cells connected in parallel.

Software:

1. Download the Arduino 1.8.5 version of software from the Arduino website using this address https://www.arduino.cc/en/Main/Software
2. Locate the Arduino libraries folder. This is often in your My Documents folder.
3. Copy the following folders and their contents into the Arduino libraries folder
    a. Narcoleptic
    b. Sodaq_DS3231-1.2.2
4. Copy the UW auto-sampler directory to your hard drive. This directory contains four software routines to operate the auto-sampler
    a. AutoSampler_Continuous_RTC
    b. AutoSampler_Discrete_RTC
    c. time_set_manually
    d. current_time.

Communication:

1. Plug in USB cable connected to auto-sampler – **Caution**: *make sure the internal D-cell battery pack is not connected at the same time as the USB*.
2. Locate the Arduino program, usually accessible via Windows Start Button in All Programs. Click to start the program.
3. Select “Tools”, “Board” for the Board Manager and then select “Arduino Pro or Pro Mini”
4. Select “Tools”, “Processor” then select “ATmega328P(5V, 16MHz)”
5. Select “Port”, and then select the correct COM port if it doesn’t select it automatically (Baud rate should be 9600,N,8,1)
6. Select “File”, “Open” and open program “current_time” from the directory you set in the Software Section 4 above.
7. Either press the 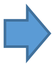 icon in the Arduino software or select “Sketch” then “Upload” to upload the program to the Pro mini. The blue status bar at the bottom will track the upload status. If successful it will display “Done uploading”. To check and see if the time is correct, click on the magnifying glass at the upper right. This will open a dialog box and display the current time on the interface. If the time is correct, close the window and you can proceed with loading any other programs. If it needs to be changed you will need to set the time manually. To do so, see step 8 below.

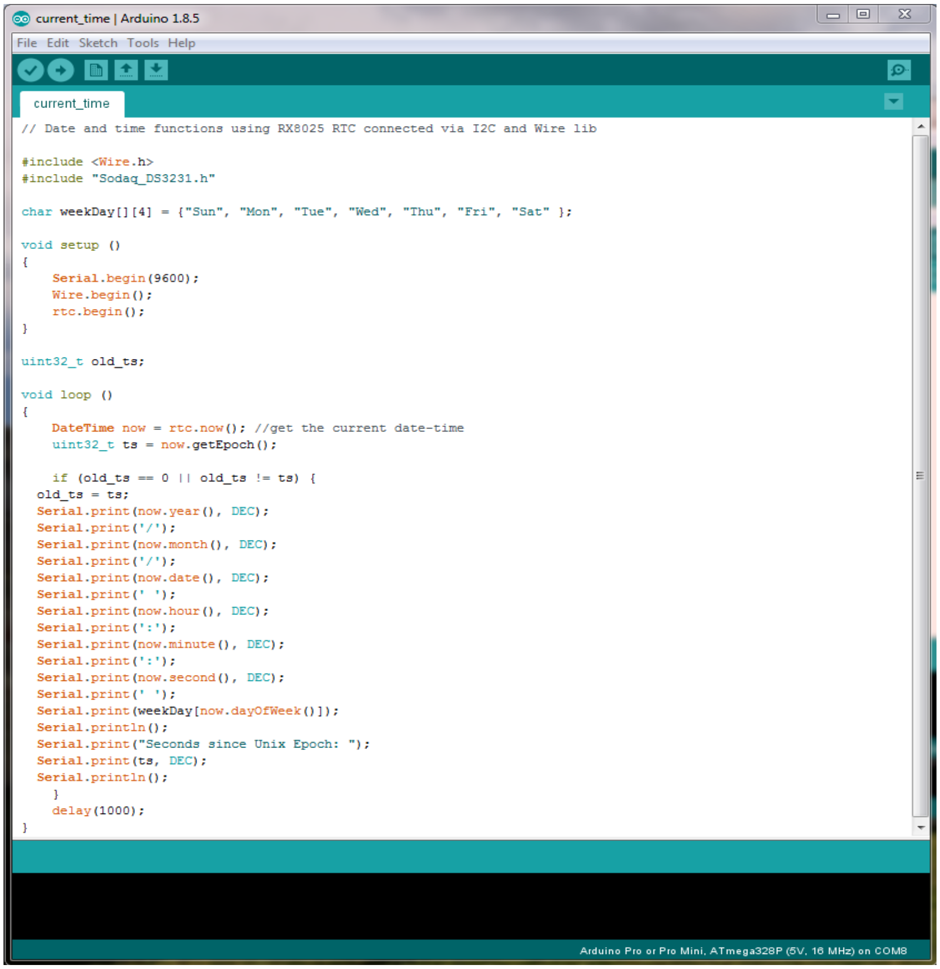

8. To set the time manually, open the program time_set_manually located in the directory you set in Software Section 4 above.

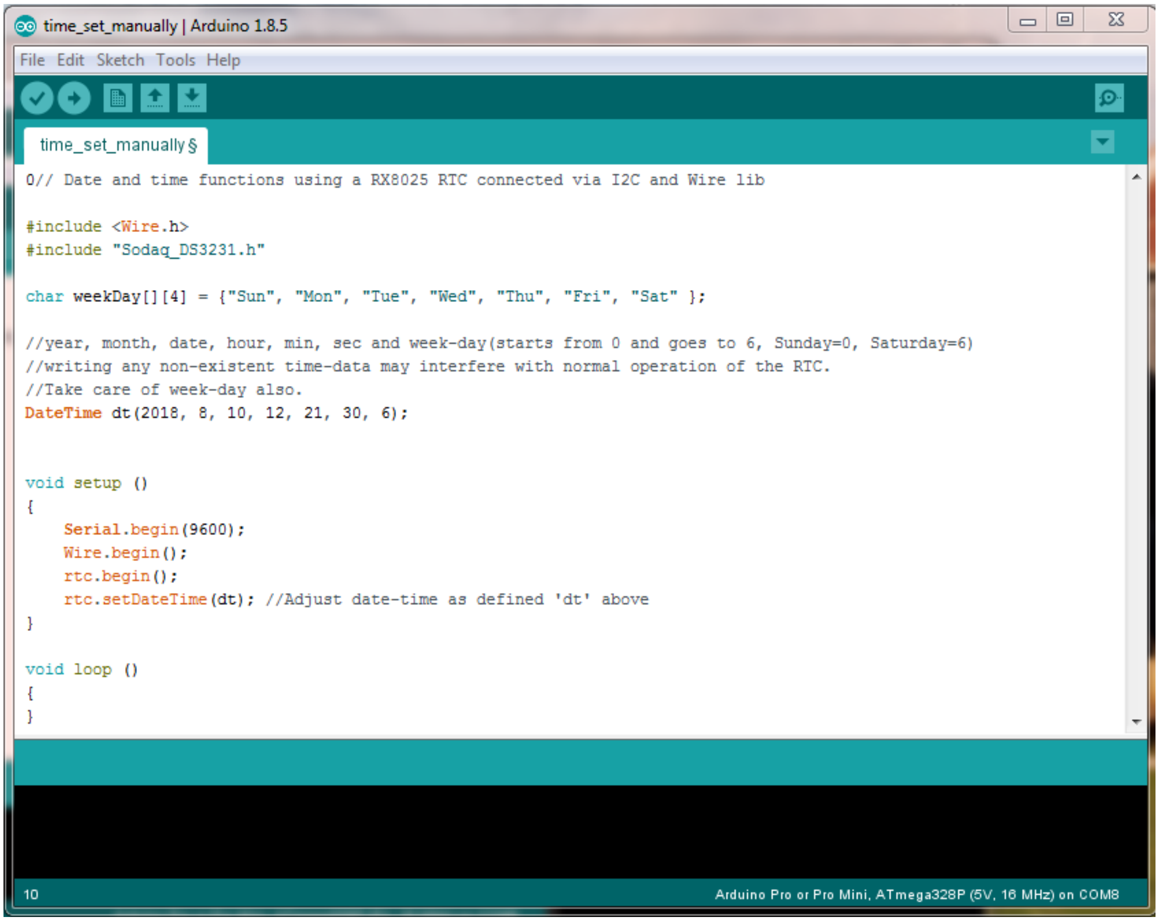

9. Scroll down to the command line that will look something like this

DateTime dt(2018, 8, 10, 12, 21, 30, 6);

Above this command line is a comment statement which begin with // that will tell you what you can enter.

//year, month, date, hour, min, sec and week-day (starts from 0 and goes to 6, Sunday=0, Saturday=6)
//writing any non-existent time-data may interfere with normal operation of the RTC.
//Take care of week-day also.

10. It takes approximately 30 seconds to upload this sketch therefore it is difficult to get the time exact. Plan ahead as you modify this line to take into account the upload delay. When ready, either press the 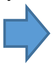 icon in the Arduino software or select “Sketch” then “Upload” to upload the program to the Pro mini. The blue status bar at the bottom will track the upload status. If successful it will display “Done uploading”. To check and see if the time is correct, upload the “current time” program as described above in Steps 6 and 7.

Once you are satisfied with the time on the real-time clock you can begin preparing the auto-sampler.

Software Preparation:

There are two modes to program the AutoSampler, Continuous Mode or Discrete Mode. Each mode requires different software.

1) **Continuous mode** is where the instrument is programmed to pump water to each port at a set time interval and will repeat after all 12 ports have been sampled. This requires the user to either change out sample containers before the next full cycle begins or recover the instrument and terminates the sequence. If sensors are attached to each port, then the system will run until the batteries are exhausted or the cycle is interrupted by the user. The program required to run the system in this mode is “AutoSampler_Continuous_RTC”.

    A. Select “File”, “Open” and open program “AutoSampler_Continuous_RTC” from the directory you set in the Software Section 4 above.

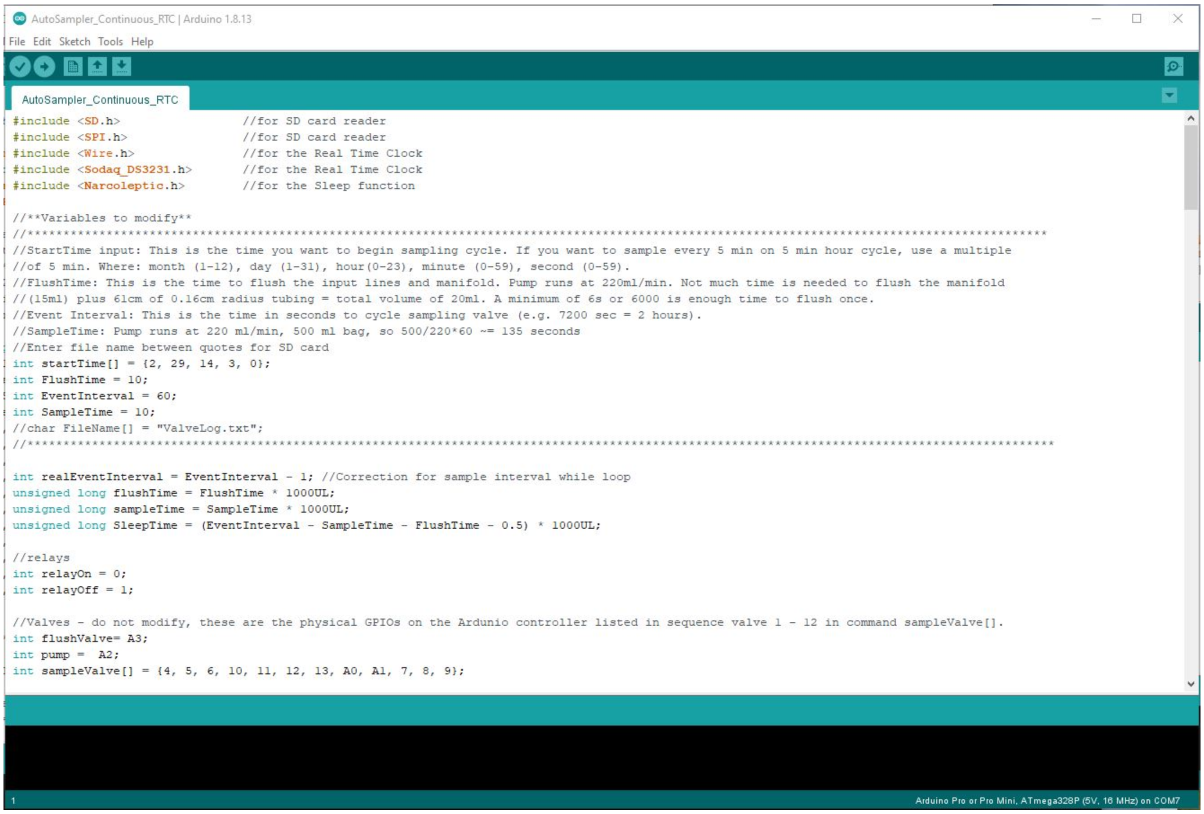

B. There are four command lines you can modify

1. startTime[] = {2, 29, 14, 3, 0};
   The variable startTime is the time and date you want the first sample to be taken. In this example 2/29 at 14:03:00 where the start year will come from the year set in the RTC will be used.
2. int FlushTime = 10;
   The variable FlushTime is how long the peristaltic pump and the flush valve will stay open, in this example, 10 seconds. The volume of the flush plumbing is ~20 ml so a minimum of 6 seconds is enough time to flush the system once. It is best to flush at least two times.
3. EventInterval = 60:
   This is the time in seconds to cycle the sampling valve. In this example it is 60 seconds. In a real deployment this interval will be much larger (e.g. 7200 seconds for a 2 hour cycle between ports for a 24 hour deployment using all 12 valve ports).
4. SampleTime = 10;
   This is the amount of time, in seconds, that the peristaltic pump will run while delivering water to each port. The pump runs at ~220 ml min^{-1} to fill a 1L Tedlar bag, the peristaltic pump needs to run for 1000/220*60 = 272 seconds. In order not to overfill the bag 260 seconds is a good safety margin.

C. When you upload this program using this example, the peristaltic pump will start at the date/time interval (startTime) by operating the peristaltic pump with the flush valve open for 10 seconds (FlushTime). After this time, the flush valve will close and the sample port will open and the peristaltic pump will continue to run for an additional 10 seconds (SampleTime), then stop and close the sample port valve. The next port will begin sampling 60 seconds later (EventInterval) and repeat the process until the system is interrupted by the user or the battery power is exhausted.

D. To upload "AutoSampler_Continuous_RTC" either press the 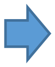 icon in the Arduino software or select "Sketch" then "Upload" to upload the program to the Pro mini. The blue status bar at the bottom will track the upload status. If successful it will display "Done uploading". When finished the sequencing will begin. Note that in this mode with only the USB cable connected to the computer, the relays will not activate the pump or the valves. Only the LEDs on the relay boards will illuminate. Allow this to go to completion to make sure the system is working.

E. Unplug the USB cable and then connect the Molex connector to the D-cell battery pack. The sequence will go through all valves and then stop. If you

want to rerun the sequence simply disconnect the Molex connector, wait a few seconds for the system to power down, and then reconnect.

F. You can also use this same sequence to flush and purge the auto-sampler. More info in the Recovery section below.

2) **Discrete mode** is where the instrument is programmed to pump water to a specific port, in sequence 1 to XX, at a specified date and time until all ports are sampled. The program required to run the system in this mode is "AutoSampler_Discrete_RTC".

A. Select "File", "Open" and open program "AutoSampler_Discrete_RTC" from the directory you set in the Software Section 4 above.

B. There are five command lines you can modify

1. sampleTime = 3000UL;
   This is the amount of time in milliseconds and the UL must be used. The pump runs at ~220 ml min^{-1}. In order to fill a 1L Tedlar bag, the pump must be on for 1000/220*60 = 272 seconds or 272000UL. In order not to overfill the bag 260 seconds is a good safety margin.
2. sleep = 1000;
   This is the amount of time for the system to sleep in between sampling intervals to save on battery consumption. It is in milliseconds.
3. flushTime = 4000;
   The variable flushTime is how long the peristaltic pump and the flush valve will stay open, in this example, 10 seconds. The volume of the flush plumbing is ~20 ml so a minimum of 6 seconds is enough time to flush the system once. It is best to flush at least two times.
4. numberOfSamples = 12;
   This is the total number of ports to be sampled. The maximum number is 12.
5. eventTime[] = {
   3, 3, 16, 15,
   3, 3, 16, 16,
   3, 3, 16, 17,
   3, 3, 16, 18,
   3, 3, 16, 19,
   3, 3, 16, 20,
   3, 3, 16, 21,
   3, 3, 16, 22,
   3, 3, 16, 23,
   3, 3, 16, 24,

3, 3, 16, 25,
3, 3, 16, 26
};

The variables used on each line above represents the Month, Day, Hour, and Minute. The year is determined based on the year used to program the RTC.

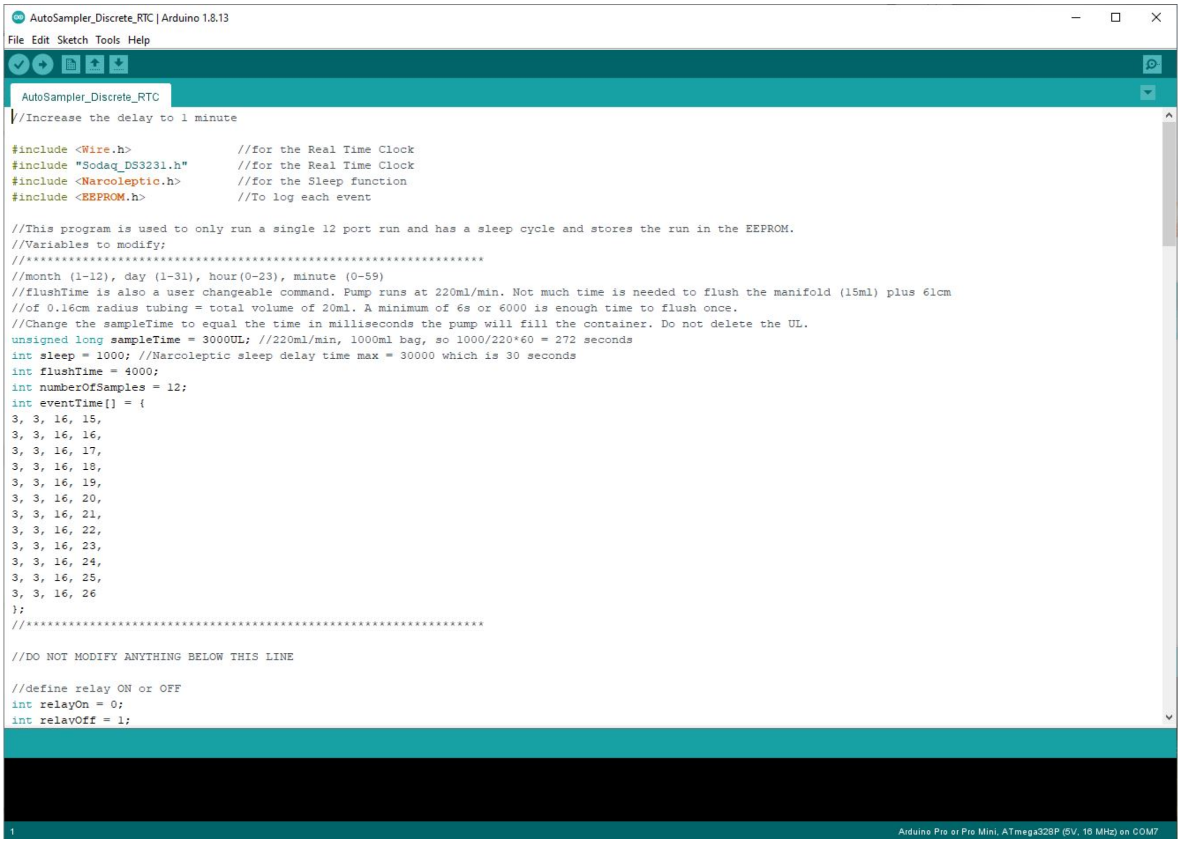

C. When you upload this program using this example, the peristaltic pump will start at the date/time interval (eventTime) by operating the peristaltic pump with the flush valve open for 4 seconds (flushTime). After this time, the flush valve will close and the sample port will open and the peristaltic pump will continue to run for an additional 3 seconds (sampleTime), then stop and close the sample port valve and sleep for 1 second (sleep). The next port will begin sampling at the date/time interval on the subsequent line and it will continue until all 12 valves are sequenced.

D. To upload “AutoSampler_Discrete_RTC” either press the 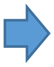 icon in the Arduino software or select “Sketch” then “Upload” to upload the program to the Pro mini. The blue status bar at the bottom will track the upload status. If successful it will display “Done uploading”. When finished the sequencing will begin. Note that in this mode, with only the USB cable connected to the computer, the relays

will not activate the pump or the valves. Only the LEDs on the relay boards will illuminate. Allow this to go to completion to make sure the system is working.

E. Unplug the USB cable and then connect the Molex connector to the battery leads on the D-cell battery pack. When you establish this connection, the sequence will begin again, this time with power going to all components. The sequence will go through all valves and then stop. If you want to rerun the sequence simply disconnect, wait a few seconds for the system to power down, and then reconnect.

F. You can also use this same sequence to flush and purge the auto-sampler. More info in the Recovery section.

Deployment:

Prior to each deployment make sure the output voltage from the battery pack is set at 12 VDC. Use a digital mulitmeter (DMM) to check the voltage of the battery pack. You can measure this voltage at two safe locations, the terminal block or on the input leads to the XL6009 booster module. A new battery pack should have slightly more than 12 VDC. A safe place to measure the output voltage is at the pins on the Molex connector or on the output leads on the XL6009 regulator/booster module. The output voltage should be set to 12 VDC, if adjustment is required, use the adjustment screw on top of the blue potentiometer on the XL6009 regulator/booster module.

If you are redeploying the system using the same/used battery pack, it is still good to check the input and output voltages to make sure the electronics and pump are seeing 12 VDC.

If you followed the steps above the USB cable is still connected to the computer and disconnected from the main battery pack. Once you have the real-time clock set and you have tested the auto-sampler you can begin the deployment process. Determine which run mode you want to use, continuous or discrete, program as outlined above, and then perform the following steps.

1. When you have completed the necessary software modifications you can save the changes using the same program name or make a new filename. Make sure to keep and original in the event you made a mistake when modifying the code. Make sure you allow enough time for deployment before the first valve sequence begins.

2. To upload your program either press the 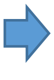 icon in the Arduino software or select “Sketch” then “Upload” to upload the program to the Pro mini. The blue status bar at the bottom will track the upload status. If successful it will display “Done uploading”. When finished, unplug the USB cable and carefully coil and store on top of the electronics.

3. With the **USB unplugged**, it is now safe to connect the Molex connector from the battery pack to the electronics.

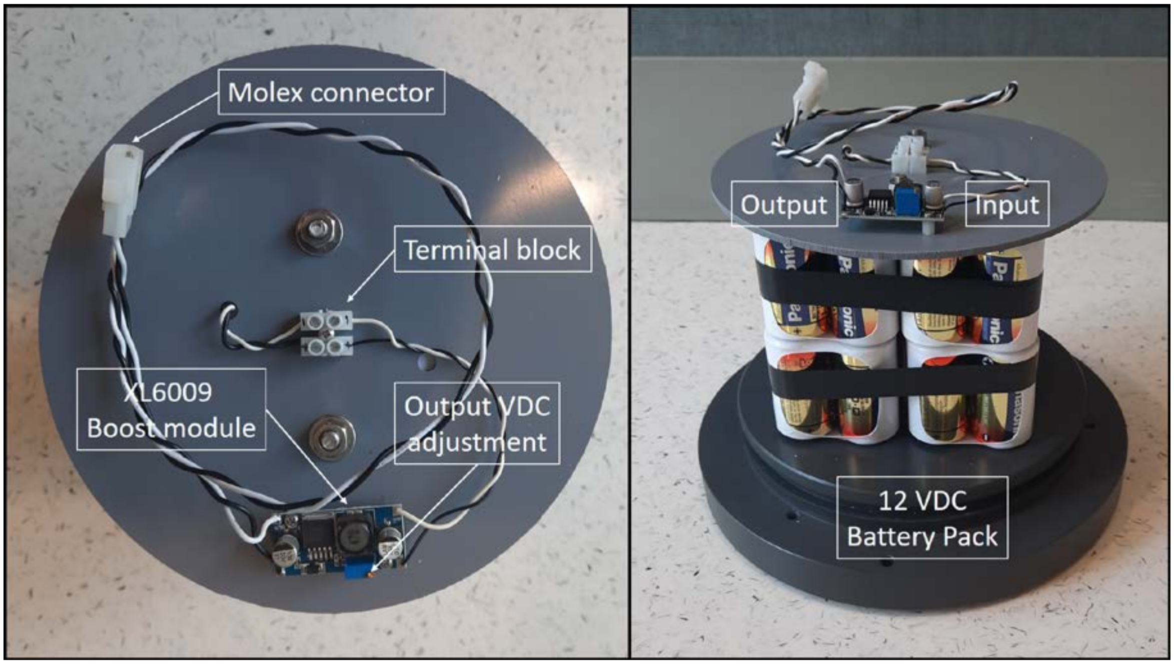

The figure above illustrates the location of the components on the battery pack disc which includes the terminal block, Molex connector, boost converter XL6009 module and the location of the output voltage adjustment screw.

4. With the power leads securely connected you can close up the pressure housings provided that the o-rings are properly greased and free of and debris.

Pressure Housing:

The pressure housing is made of PVC with cemented rings on both ends of the housing. The end caps are piston style and are designed to overlap onto the rings to secure the end cap for deployment using two 316 stainless steel 1.75” long ¼-20 hex-head bolts and flat washers. Do not over tighten these bolts since they are only needed to hold the end cap flush to the housing. The same 1.75” hex-head bolts can be used as “jack bolts” to remove the end caps. There are three threaded holes on both end caps. You will need to use all three holes and three jack bolts to evenly extend the end cap from the flange ring.

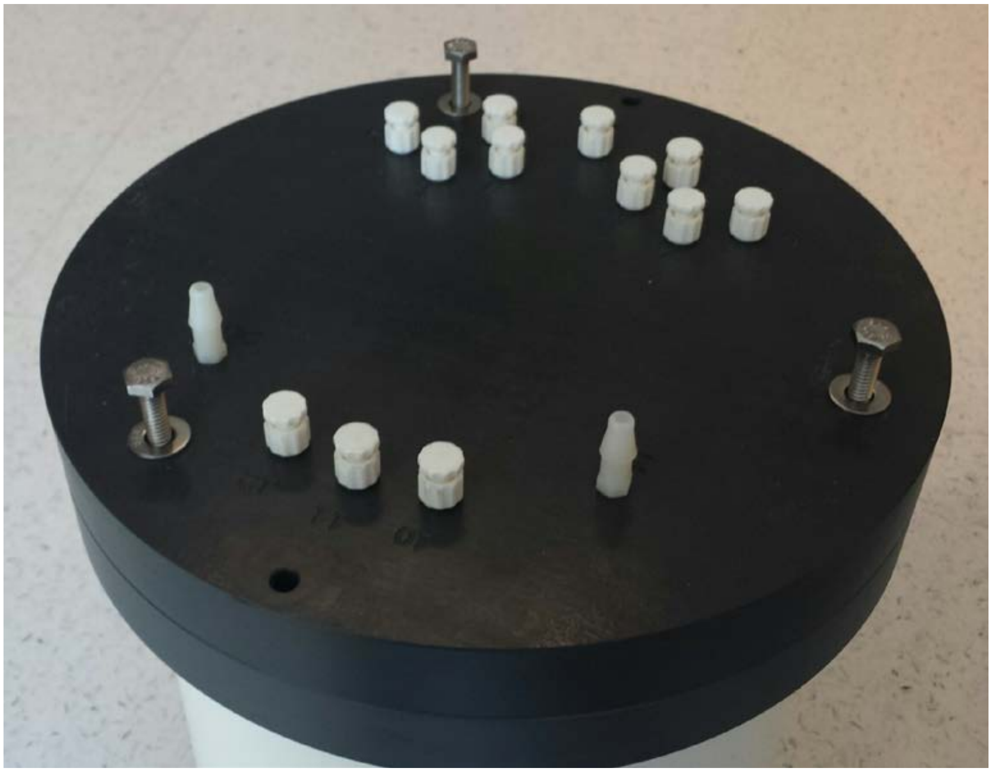

The figure above shows the location of the jack bolts that are used to remove the end caps. The two open holes are used to secure the end cap to the ring flange.

Specification:

Depth rating: No not exceed 20 m
Overall height: 40.64 cm
Overall diameter is 25.4 cm
Internal diameter: 20.3 cm
Internal height with end caps assembled: 30.48cm
Battery pack: 12 VDC - 4 D-cell packs (8 batteries in serial at 12 VDC/each) linked in parallel
Tubing: Silicone 0.25” OD x 0.125” ID x 12” long

Recovery and cleaning:

Upon recovery it is important to flush out the system. Use the “AutoSampler_Continuous_RTC” program for this procedure. To begin, remove the bolts securing the bottom end cap (with the battery pack) to the flange ring then remove the end cap using the jack bolt.

With the battery pack removed, disconnect both the Red and Black wires connected to the terminal block on the top of the battery pack. Now it is safe to plug in the USB cable to your computer. Before uploading the “AutoSampler_Continuous_RTC”

you should modify the program by changing the four parameters to use shorter intervals to facilitate the flushing of the system. This includes an early startTime (e.g. a few minutes into the future), FlushTime (e.g. 20 sec), EventInterval (e.g., 60 sec), and SampleTime (e.g. 30 sec).

Attach a tube to the inlet port and place the tube into either filtered seawater or freshwater and the exit ports (including the flush port) into a waste container. A minimum of 2 rinse cycles is recommended using water. A third or final rinse should be with no water. The peristaltic pump will pump air and will purge the system of most of the water in the lines. The benefit of using the continuous mode is that you can let it go as many cycles as you want before terminating.

Once you have made this change, upload the program to the Pro mini as outlined below.

1. Upload “AutoSampler_Continuous_RTC” with the shorter parameters and either press the 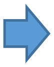 icon in the Arduino software or select “Sketch” then “Upload” to upload the program to the Pro mini. The blue status bar at the bottom will track the upload status. If successful it will display “Done uploading”. When finished the sequencing will begin, but in this mode, i.e., with only the USB cable connected to the computer the relays will not activate the pump or the valves. You can disconnect the USB and this will stop the sequence until it is restarted when later reconnected to the battery pack.

2. If you haven’t already, connect tubing to the in and out ports. Place the in tubing into a container of clean filtered seawater or freshwater and the out tubing to drain in a separate container. If you are flushing all valves you will need to connect tubes to each exit port and place in the container with the out tube.

3. Unplug the USB cable and then connect the Molex connector to the battery leads on the D-cell battery pack. When you establish this connection, the sequence will begin again, this time with power going to all components. The sequence will go through all valves and then stop. If you want to rerun the sequence simply disconnect, wait a few seconds for the system to power down, and then reconnect.

4. Since you are using the continuous mode the system will continue to flush until the user stops the sequence. For the last sequence, remove the inlet tube from the filtered water and let the system purge with air using the peristaltic pump.

5. To stop the sequence, open the case and disconnect the Molex connector.

Battery Life Calculation and Peristaltic tubing life:

The estimated power consumption of the electronics (Arduino Pro Mini, real-time clock, and relay boards) is ~110 mA, each Clippard pinch valve has a factory specification of 400 mA, and the Thomas 10/50 peristaltic pump has a factory specification of 540 mA for a total of 1040 mA. This amounts to ~0.017 Ah min^{-1} of operation. To calculate the total deployment Ah, the following equation can be used as an estimate.

Logic and electronics (controller, real-time clock, relays)	110 mA	
Clippard pinch valve	400 mA	
Thomas 10/50 peristaltic pump	540 mA	
Total energy consumption	1040 mA	(0.017 Ah min^{-1})

Deployment Ah = ((0.017 * sampling time in minutes) * (# of samples * # of cycles).

Deployment Example: A deployment using 2 minute pumping time, taking 12 samples day^{-1}, deployed for 15 days will have a total of 180 sample cycles consuming ~6 Ah or about 10% of the total 60 Ah battery pack capacity. This would amount to ~1.2 VDC in battery voltage. A DC to DC boost converter/regulator module (XL6009) can be used on the battery pack to provide a steady 12 VDC output to the electronics and pump as the battery pack power decreases. With this module the input voltage can drop to ~5 VDC and still maintain the set 12 VDC output allowing for 58% (~35 Ah of the total 60 Ah) of the battery pack available to power the AutoSampler.

Example: Deployment Ah = ((0.017 * 2 minutes) * (12 samples * 15 cycles).
Deployment Ah = 6.12

The Thomas 10/50 peristaltic pump specifications states that the Novoprene tubing has 500 hr. lifetime and the DC motor 1000 hr. This amounts to approximately 20 days of continuous operation before the pump tubing needs to be replaced but should be taken into account prior to deployment.

Biofouling and Clogging prevention:

1. To help keep the pressure case protected during deployment, PVC tape (McMaster PN: 6029T96) can be used to wrap the body and end caps. Make sure the tape will not interfere with the input, output, or sampling ports. The tape can also be used to place identification markings for the deployment. Upon recovery the tape can be removed along with any growth and the markings.

2. To minimize clogging of the pumping and valve system a filter mesh can be used over the input port as shown below. This can be made by cutting a piece of mesh

2x the diameter of the input tube and using cable ties to attach it to the barbed fitting on the input port and then use another cable tie to close off the top.

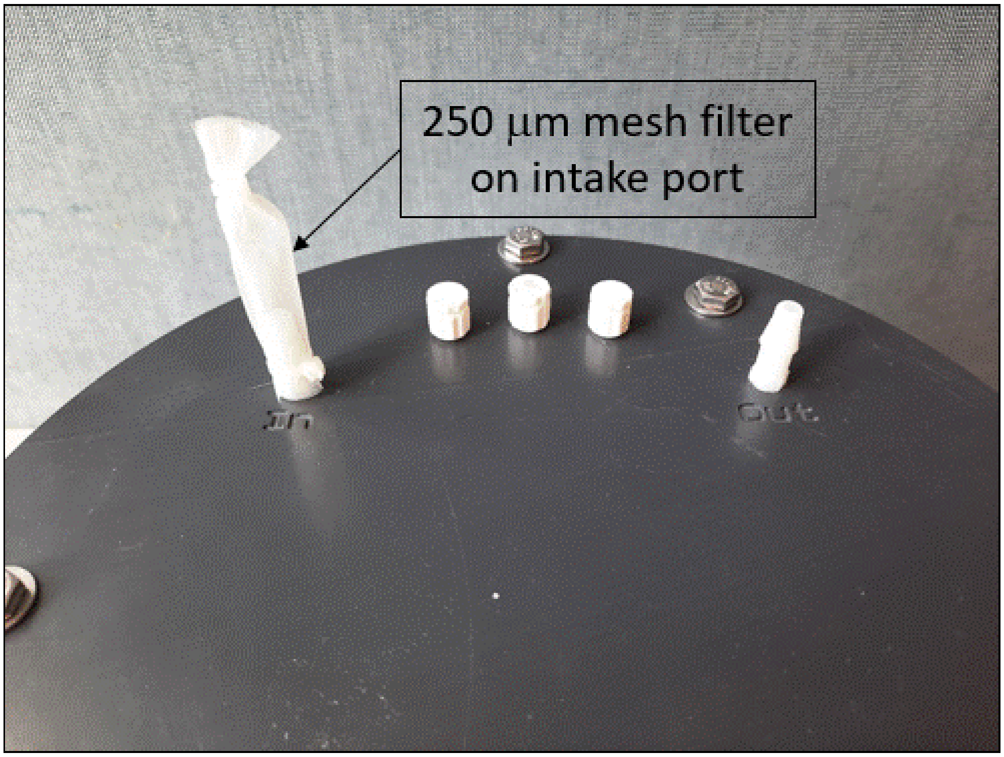

Wiring schematic:

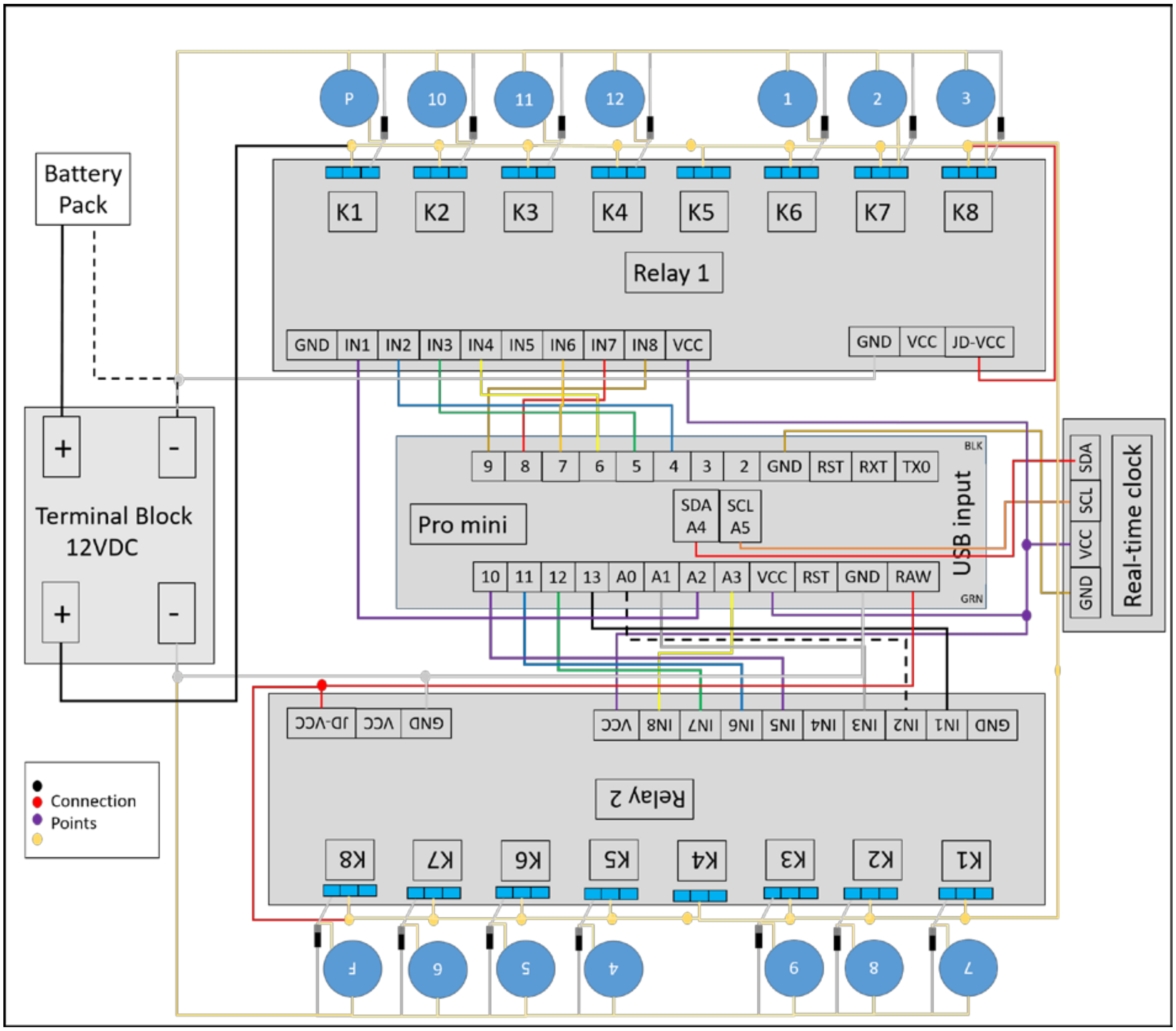

AutoSampler wiring schematic. The system consist of an Arduino Pro-mini, two 8-channel relay boards, a real-time clock, and 12VDC battery pack.
