# Supplementary material for: Autonomous submersible multiport water sampler
Source: HardwareX. 2021 Apr 22;9:e00197. doi: 10.1016/j.ohx.2021.e00197 (PMC9041238; doi:10.1016/j.ohx.2021.e00197)
Supplement: Supplementary data 6 [file mmc6.pdf]

```
#include <SD.h>                        //for SD card reader
#include <SPI.h>                       //for SD card reader
#include <Wire.h>                      //for the Real Time Clock
#include <Sodaq_DS3231.h>     //for the Real Time Clock
#include <Narcoleptic.h>          //for the Sleep function

//**Variables to modify**
//*********************************************************************************
//StartTime input: This is the time you want to begin sampling cycle. If you want to sample every 5 min
//on 5 min hour cycle, use a multiple
//of 5 min. Where: month (1-12), day (1-31), hour (0-23), minute (0-59), second (0-59).
//FlushTime: This is the time to flush the input lines and manifold. Pump runs at 220ml/min. Not much
//time is needed to flush the manifold
//(15ml) plus 61cm of 0.16cm radius tubing = total volume of 20ml. A minimum of 6s or 6000 is enough
//time to flush once.
//Event Interval: This is the time in seconds to cycle sampling valve (e.g. 7200 sec = 2 hours).
//SampleTime: Pump runs at 220 ml/min, 500 ml bag, so 500/220*60 ~= 135 seconds
//Enter file name between quotes for SD card
int startTime[] = {2, 29, 14, 3, 0};
int FlushTime = 10;
int EventInterval = 60;
int SampleTime = 10;
//char FileName[] = "ValveLog.txt";
//*********************************************************************************
int realEventInterval = EventInterval - 1; //Correction for sample interval while loop
unsigned long flushTime = FlushTime * 1000UL;
unsigned long sampleTime = SampleTime * 1000UL;
unsigned long SleepTime = (EventInterval - SampleTime - FlushTime - 0.5) * 1000UL;

//relays
int relayOn = 0;
int relayOff = 1;

//Valves - do not modify, these are the physical GPIOs on the Ardunio controller listed in sequence valve
1 - 12 in command sampleValve[].
int flushValve= A3;
int pump =  A2;
int sampleValve[] = {4, 5, 6, 10, 11, 12, 13, A0, A1, 7, 8, 9};

//index variables
int startTimeIndex = 0;
int addr = 0;             //for logging each autosampling event

//Date variables
```

```
DateTime now;
int RTCmonth;             //Real Time Clock
int RTCday;
int RTChour;
int RTCminute;
int RTCsecond;
int startMonth;           //declared month, day, hour, or minute to sample
int startDay;
int startHour;
int startMinute;
int startSecond;

//for SD card
//File myFile;
//int pinCS = 10; // Pin 10 on Arduino Uno

void setup()
{

  // Sets all the pins to output mode
  for(int i = 0; i <= 11; i++)
   {
    pinMode(sampleValve[i], OUTPUT);
   }

  for(int i = 0; i <= 11; i++)
   {
    digitalWrite(sampleValve[i], relayOff);
   }

  pinMode(pump, OUTPUT);
  pinMode(flushValve, OUTPUT);

  digitalWrite(pump, relayOff);
  digitalWrite(flushValve, relayOff);

  // for SD card
  // pinMode(pinCS, OUTPUT);

  // begin
  Serial.begin(9600);
  Wire.begin();
  rtc.begin();
  // SD.begin();
```

```
  Serial.println("********Parameter Header*********");
  Serial.print("Valve Event Interval= ");
  Serial.print(EventInterval);
  Serial.println(" seconds");
  Serial.print("Flush Valve Interval= ");
  Serial.print(FlushTime);
  Serial.println(" seconds");
  Serial.print("Sample Time= ");
  Serial.print(SampleTime);
  Serial.println(" seconds");
  delay (100);
  //get the current date-time
  now = rtc.now();
  Serial.print("Upload Time= ");
  Serial.print(now.year(), DEC);
  Serial.print('/');
  Serial.print(now.month(), DEC);
  Serial.print('/');
  Serial.print(now.date(), DEC);
  Serial.print(' ');
  Serial.print(now.hour(), DEC);
  Serial.print(':');
  Serial.print(now.minute(), DEC);
  Serial.print(':');
  Serial.println(now.second(), DEC);
  Serial.println("**********************************");
  delay (100);

startMonth=startTime[startTimeIndex];
    now = rtc.now();
    RTCmonth = now.month();
        while (RTCmonth!=startMonth){
          delay(100);
          now = rtc.now();
          RTCmonth = now.month();}
    startTimeIndex ++;
startDay=startTime[startTimeIndex];
    now = rtc.now();
    RTCday = now.date();
          while (RTCday!=startDay){
        delay(100);
        now = rtc.now();
        RTCday = now.date();}
```

```
    startTimeIndex ++;
 startHour=startTime[startTimeIndex];
    now = rtc.now();
    RTChour = now.hour();
       while (RTChour!=startHour){
         delay(100);
         now = rtc.now();
         RTChour = now.hour();}
    startTimeIndex ++;
 startMinute=startTime[startTimeIndex];
    now = rtc.now();
    RTCminute = now.minute();
        while (RTCminute < startMinute){
          delay(100);
          now = rtc.now();
          RTCminute = now.minute();}
 startSecond=startTime[startTimeIndex];
    now = rtc.now();
    RTCsecond = now.minute();
        while (RTCsecond < startSecond){
          delay(100);
          now = rtc.now();
          RTCsecond = now.second();}
    startTimeIndex ++;

} //End void setup loop

void loop()
{  //Begin infinite loop

 // Valve sequencing loop
 // for (int x = 0; x < 12; ++x){  // Begin for loop

    DateTime sampleStartTime = rtc.now();

    pinMode (sampleValve[x], OUTPUT);
    digitalWrite (sampleValve[x], relayOff);

    //get the current date-time for serial monitor
    now = rtc.now();
    Serial.print(now.year(), DEC);
    Serial.print('/');
    Serial.print(now.month(), DEC);
    Serial.print('/');
```

```
  Serial.print(now.date(), DEC);
  Serial.print(' ');
  Serial.print(now.hour(), DEC);
  Serial.print(':');
  Serial.print(now.minute(), DEC);
  Serial.print(':');
  Serial.print(now.second(), DEC);
  Serial.print(' ');
  Serial.print(x+1);
  Serial.println("_Sequence_begin");
  delay (100);

   if (rtc.now().get() - sampleStartTime.get() <= realEventInterval) {
     digitalWrite(flushValve, relayOn); // Open flush valve
     digitalWrite(pump, relayOn); // Start pump
     delay(flushTime);
     digitalWrite(flushValve, relayOff); // Close flush valve
     digitalWrite(sampleValve[x], relayOn); // OPEN VALVE
     delay(sampleTime);
     digitalWrite(sampleValve[x], relayOff); // CLOSE VALVE
     digitalWrite(pump, relayOff); // Stop pump

     now = rtc.now(); //get the current date-time to write to SD Card
     Serial.print(now.year(), DEC);
     Serial.print('/');
     Serial.print(now.month(), DEC);
     Serial.print('/');
     Serial.print(now.date(), DEC);
     Serial.print(' ');
     Serial.print(now.hour(), DEC);
     Serial.print(':');
     Serial.print(now.minute(), DEC);
     Serial.print(':');
     Serial.print(now.second(), DEC);
     Serial.print(' ');
     Serial.print(x+1);
     Serial.println("_Sequence_end");
     delay (100);

       while (rtc.now().get() - sampleStartTime.get() <= realEventInterval) {
         Narcoleptic.delay(SleepTime);
       }  //End while loop and continue
   } // End if
} // End for loop
```

```
} // End void loop
// GPIO for the RTC and SD CARD reader - these hardwired outputs cannot be modified.

//CLOCK required pinouts
//VCC -> Arduino 5V
//GND -> Arduino GND
//SCL -> SCL or A5
//SDA -> SDA or A4

//SD CARD required pinouts
//VCC -> Arduino 5V
//GND -> Arduino GND
//MISO -> 12
//MOSI -> 11
//SCK -> 13
//CS -> 10
//Code written by D. Mucciarone 5-17-19
```
