# Supplementary material for: Autonomous submersible multiport water sampler
Source: HardwareX. 2021 Apr 22;9:e00197. doi: 10.1016/j.ohx.2021.e00197 (PMC9041238; doi:10.1016/j.ohx.2021.e00197)
Supplement: Supplementary data 7 [file mmc7.pdf]

```
#include <Wire.h>                   //for the Real Time Clock
#include "Sodaq_DS3231.h"    //for the Real Time Clock
#include <Narcoleptic.h>        //for the Sleep function
#include <EEPROM.h>             //To log each event

//This program is used to only run a single 12 port run and has a sleep cycle and stores the run in the
//EEPROM.
//Variables to modify;
//*****************************************************************
//month (1-12), day (1-31), hour (0-23), minute (0-59)
//flushTime is also a user changeable command. Pump runs at 220ml/min. Not much time is needed to
//flush the manifold (15ml) plus 61cm
//of 0.16cm radius tubing = total volume of 20ml. A minimum of 6s or 6000 is enough time to flush once.
//Change the sampleTime to equal the time in milliseconds the pump will fill the container. Do not
//delete the UL.
unsigned long sampleTime = 3000UL; //220ml/min, 1000ml bag, so 1000/220*60 = 272 seconds
int sleep = 1000; //Narcoleptic sleep delay time max = 30000 which is 30 seconds
int flushTime = 4000;
int numberOfSamples = 12;
int eventTime[] = {
3, 3, 16, 15,
3, 3, 16, 16,
3, 3, 16, 17,
3, 3, 16, 18,
3, 3, 16, 19,
3, 3, 16, 20,
3, 3, 16, 21,
3, 3, 16, 22,
3, 3, 16, 23,
3, 3, 16, 24,
3, 3, 16, 25,
3, 3, 16, 26
};
//*****************************************************************

//DO NOT MODIFY ANYTHING BELOW THIS LINE

//define relay ON or OFF
int relayOn = 0;
int relayOff = 1;
//Pin1=V1
//Valves
int flushValve= A3;
int pump =  A2;
```

```
int sampleValve[] = {7, 8, 9, 10, 11, 12, 13, A0, A1, 4, 5, 6};//valve 1,2,3,4,5,6,7,8,9,10,11,12

//index variables
int sampleValveIndex;      //pointer to sampleValve to open
int eventTimeIndex = 0;    //pointer to declared month, day, hour, or minute
int addr = 0;              //for logging each autosampling event

//Date variables
DateTime now;
int RTCmonth;              //Real Time Clock
int RTCday;
int RTChour;
int RTCminute;
int eventMonth;            //declared month, day, hour, or minute to sample
int eventDay;
int eventHour;
int eventMinute;

void setup()
{

//Sets all the pins to output mode
  for(int i = 0; i <= 11; i++)
    {
     pinMode(sampleValve[i], OUTPUT);
    }

  for(int i = 0; i <= 11; i++)
    {
     digitalWrite(sampleValve[i], relayOff);
    }

pinMode(pump, OUTPUT);
pinMode(flushValve, OUTPUT);

digitalWrite(pump, relayOff);
digitalWrite(flushValve, relayOff);

//begin
Serial.begin(9600);
Wire.begin();
rtc.begin();
```

```
//Modify the lines below to write to SD card for nano, substitute Serial.print with myFile.print
now = rtc.now().get(); //get the current date-time to write to SD Card
   Serial.println("***********Parameter Header*****************");
   Serial.print(now.year(), DEC);
   Serial.print('/');
   Serial.print(now.month(), DEC);
   Serial.print('/');
   Serial.print(now.date(), DEC);
   Serial.print(' ');
   Serial.print(now.hour(), DEC);
   Serial.print(':');
   Serial.print(now.minute(), DEC);
   Serial.print(':');
   Serial.println(now.second(), DEC);
   Serial.print("Number of samples = ");
   Serial.println(numberOfSamples);
   Serial.print("Flush Time (sec) = ");
   Serial.println(flushTime/1000);
   Serial.print("Sample Pump time (sec) = ");
   Serial.println(sampleTime/1000);
   Serial.println("*******************************************");
   delay(100);

//loops through the samples to fill

for(sampleValveIndex = 0; sampleValveIndex <= (numberOfSamples - 1); sampleValveIndex++){
  eventMonth=eventTime[eventTimeIndex];
    now = rtc.now().get();
    RTCmonth = now.month();
      while (RTCmonth!=eventMonth){
        Narcoleptic.delay(sleep);
        now = rtc.now();
        RTCmonth = now.month();}
    eventTimeIndex ++;
  eventDay=eventTime[eventTimeIndex];
    now = rtc.now().get();
    RTCday = now.date();
      while (RTCday!=eventDay){
        Narcoleptic.delay(sleep);
        now = rtc.now();
        RTCday = now.date();}
    eventTimeIndex ++;
  eventHour=eventTime[eventTimeIndex];
    now = rtc.now().get();
```

```
   RTChour = now.hour();
     while (RTChour!=eventHour){
       Narcoleptic.delay(sleep);
       now = rtc.now();
       RTChour = now.hour();}
   eventTimeIndex ++;
 eventMinute=eventTime[eventTimeIndex];
   now = rtc.now().get();
   RTCminute = now.minute();
     while (RTCminute < eventMinute){
       Narcoleptic.delay(sleep);
       now = rtc.now();
       RTCminute = now.minute();}
   eventTimeIndex ++;

 digitalWrite(flushValve, relayOn);
 digitalWrite(pump, relayOn);
 delay(flushTime);
 digitalWrite(sampleValve[sampleValveIndex], relayOn);
 digitalWrite(flushValve, relayOff);
//Sample time in milliseconds
 delay(sampleTime);
 digitalWrite(pump, relayOff);
 digitalWrite(sampleValve[sampleValveIndex], relayOff);

//Modify the lines below to write to SD card for nano, substitute Serial.print with myFile.print
 now = rtc.now().get(); //get the current date-time to write to SD Card
  Serial.print(now.year(), DEC);
  Serial.print('/');
  Serial.print(now.month(), DEC);
  Serial.print('/');
  Serial.print(now.date(), DEC);
  Serial.print(' ');
  Serial.print(now.hour(), DEC);
  Serial.print(':');
  Serial.print(now.minute(), DEC);
  Serial.print(':');
  Serial.print(now.second(), DEC);
  Serial.print(' ');
  Serial.print(sampleValveIndex+1);
  Serial.println("_Sample collected");
  delay(100UL);
 }
```

```
}

void loop() {
  Narcoleptic.delay(sleep);
}

//CLOCK required pinouts
//VCC -> Arduino 5V
//GND -> Arduino GND
//SCL -> SCL or A5
//SDA -> SDA or A4

//SD CARD required pinouts
//VCC -> Arduino 5V
//GND -> Arduino GND
//MISO -> 12
//MOSI -> 11
//SCK -> 13
//CS -> 10
//Code written by D. Mucciarone 5-17-19
```
