# Supplementary material for: Autonomous submersible multiport water sampler
Source: HardwareX. 2021 Apr 22;9:e00197. doi: 10.1016/j.ohx.2021.e00197 (PMC9041238; doi:10.1016/j.ohx.2021.e00197)
Supplement: Supplementary data 8 [file mmc8.pdf]

```
// Date and time functions using RX8025 RTC connected via I2C and
Wire lib

#include <Wire.h>
#include "Sodaq_DS3231.h"

char weekDay[][4] = {"Sun", "Mon", "Tue", "Wed", "Thu", "Fri",
"Sat" };

void setup () {
    Serial.begin(9600);
    Wire.begin();
    rtc.begin();
}

uint32_t old_ts;

void loop () {
    DateTime now = rtc.now(); //get the current date-time
    uint32_t ts = now.getEpoch();

    if (old_ts == 0 || old_ts != ts) {
     old_ts = ts;
     Serial.print(now.year(), DEC);
     Serial.print('/');
     Serial.print(now.month(), DEC);
     Serial.print('/');
     Serial.print(now.date(), DEC);
     Serial.print(' ');
     Serial.print(now.hour(), DEC);
     Serial.print(':');
     Serial.print(now.minute(), DEC);
     Serial.print(':');
     Serial.print(now.second(), DEC);
     Serial.print(' ');
     Serial.print(weekDay[now.dayOfWeek()]);
     Serial.println();
     Serial.print("Seconds since Unix Epoch: ");
     Serial.print(ts, DEC);Serial.println();
    }
    delay(1000);
}
```
